# Ancient genomes of Sitka black-tailed deer show evidence for early postglacial dispersal along the Pacific Northwest Coast of North America

**DOI:** 10.1186/s13059-026-04233-5

**Published:** 2026-08-05

**Authors:** Flavio Augusto da Silva Coelho, Crystal M. Tomlin, Karlee K. Prince, Duncan McLaren, Daryl Fedje, Emily Latch, James R. Heffelfinger, James F. Baichtal, Sandra L. Talbot, Timothy H. Heaton, Charlotte Lindqvist

**Affiliations:** 1 Department of Biological Sciences, University at Buffalo, Buffalo, NY USA; 2 Biology Department, Trent University, Peterborough, Canada; 3 Department of Earth Sciences, University at Buffalo, Buffalo, NY USA; 4 Hakai Institute, Heriot Bay, British Columbia, Canada; 5 Department of Biological Sciences, University of Wisconsin, Milwaukee, WI USA; 6 Terrestrial Wildlife Branch, Arizona Game and Fish Department, Phoenix, AZ USA; 7 Blackpowder Consulting, Thorne Bay, AK USA; 8 Far Northwestern Institute of Art and Science, Anchorage, AK USA; 9 Department of Earth Sciences, University of South Dakota, Vermillion, SD USA

**Keywords:** Paleogenomics, Southeast Alaska, Last Glacial Maximum, Postglacial dispersal, *Odocoileus*, Sitka black-tailed deer

## Abstract

**Background:**

The Pacific Northwestern region of North America is inhabited by two distinct subspecies of black-tailed deer, Columbian (*Odocoileus hemionus columbianus*) and Sitka (*O. h. sitkensis*) black-tailed deer, which exhibit a complex evolutionary history shaped by survival in glacial refugia, postglacial expansion, incomplete lineage sorting, and recent introgression. These dynamics make them an ideal system for examining population responses to past climate change. However, limited temporal sampling in previous genetic studies has obscured the timing and mechanisms of postglacial dispersal, and the divergence between the two black-tailed deer subspecies remains unresolved.

**Results:**

We analyze genome-scale data from ancient and modern deer spanning the last 13,500 years to reconstruct the evolutionary history of Sitka black-tailed deer in Southeast Alaska. Fossil and genomic evidence supports a primary postglacial expansion from a southern refugium, with asynchronous deglaciation and fluctuating sea levels along the Pacific Northwest Coast influencing northward colonization. Early Southeast Alaska samples (~ 9.2–8.5 cal kyr B.P.) contain mule deer mitochondrial haplotypes, indicating past contact or incomplete lineage sorting, while later individuals (< 6 cal kyr B.P.) carry Sitka black-tailed deer haplotypes, suggesting lineage turnover following mid-Holocene climatic cooling.

**Conclusions:**

Our results reject the hypothesis of a distinct northern refugium for Sitka black-tailed deer and instead support postglacial divergence from a shared black-tailed deer refugium south of the Cordilleran Ice Sheet. The asynchronous retreat of ice and dynamic sea-level history fostered repeated dispersal pulses, localized isolation, and secondary contact, shaping the present-day genetic and morphological diversity of *O. hemionus*.

**Supplementary Information:**

The online version contains supplementary material available at https://doi.org/10.1186/s13059-026-04233-5.

## Background

During the Last Glacial Maximum (LGM; 26–17 thousands of years ago; ka), the Cordilleran and Laurentide Ice Sheets extensively covered North America, reshaping landscapes and constraining population connectivity [1, 2]. As the ice sheets retreated, new migration routes emerged, allowing recolonization of previously glaciated regions. An interior ice-free corridor connecting Beringia to southern North America became ecologically viable only after ~ 13.5 ka [3–5]. In contrast, geological evidence suggests that a Northwestern Pacific coastal route may have been available as early as ~ 18.5–16 ka, following the retreat of ice from the outer coastal regions of Alaska and British Columbia [1, 6–10]. However, due to substantially lower sea levels, portions of the outer coastal margin may have been ice-free during the LGM.

Although early studies proposed ice-free conditions in parts of the Alexander Archipelago, Southeast Alaska (SEAK) [11], recent cosmogenic exposure dating [1, 9, 10] demonstrates that areas above modern sea level were fully glaciated during a local LGM (~ 20–17 ka), implying that if such refugia existed, they were largely restricted to now-submerged continental shelves exposed by lower sea levels. Extensive Late Pleistocene mammalian remains from SEAK document pronounced faunal turnover across this interval [3, 12–16], with some taxa persisting through glaciation while others, including Sitka black-tailed deer (*Odocoileus hemionus sitkensis*), appear only following deglaciation [14]. As a highly mobile herbivore capable of rapid postglacial expansion [17], black-tailed deer serves as an ideal species for resolving the timing and routes of coastal recolonization and for assessing how Late Pleistocene climate change shaped population connectivity and demographic history in northwestern North America.

Today, *Odocoileus hemionus* occupies a broad range across western North America, extending from Mexico to Alaska [18], and comprises five subspecies grouped into two major lineages: mule deer (*O. h. hemionus*, *O. h. cerrosensis*, and *O. h. sheldoni*) and black-tailed deer (*O. h. columbianus* and *O. h. sitkensis*) [18]. Despite being conspecific, these two lineages differ by 5–8% at mitochondrial loci [19–22], whereas nuclear genomic data reveal substantially lower levels of differentiation [21, 23–25], consistent with recent divergence and ongoing or historical gene flow. In addition, *Odocoileus hemionus* shares mitochondrial haplotypes with the close relative, white-tailed deer (*O. virginianus*) [26], likely reflecting incomplete lineage sorting.

The black-tailed deer lineage comprises *O. h. columbianus*, distributed from northern California to central British Columbia, and *O. h. sitkensis*, which ranges from central British Columbia to Southeast Alaska, with documented 20th-century introductions to Haida Gwaii and Kodiak Island [27–29]. Although these two black-tailed deer subspecies are genetically and morphologically distinct, their evolutionary history remains incompletely understood. Previous studies based on mitochondrial DNA and microsatellite markers support a hypothesis in which both subspecies survived the LGM in southern coastal refugia along present-day Oregon and Washington [23, 30], followed by postglacial northward dispersal once conditions became favorable. However, these studies relied exclusively on modern individuals, which capture only a fraction of past genetic diversity. Because contemporary genetic patterns may result from multiple demographic scenarios, inferences based solely on modern data are inherently limited. For example, the low genetic diversity reported in Sitka black-tailed deer could reflect survival in small refugial populations, postglacial range expansion, or a combination of both [31].

## Results

**Fig. 1:**
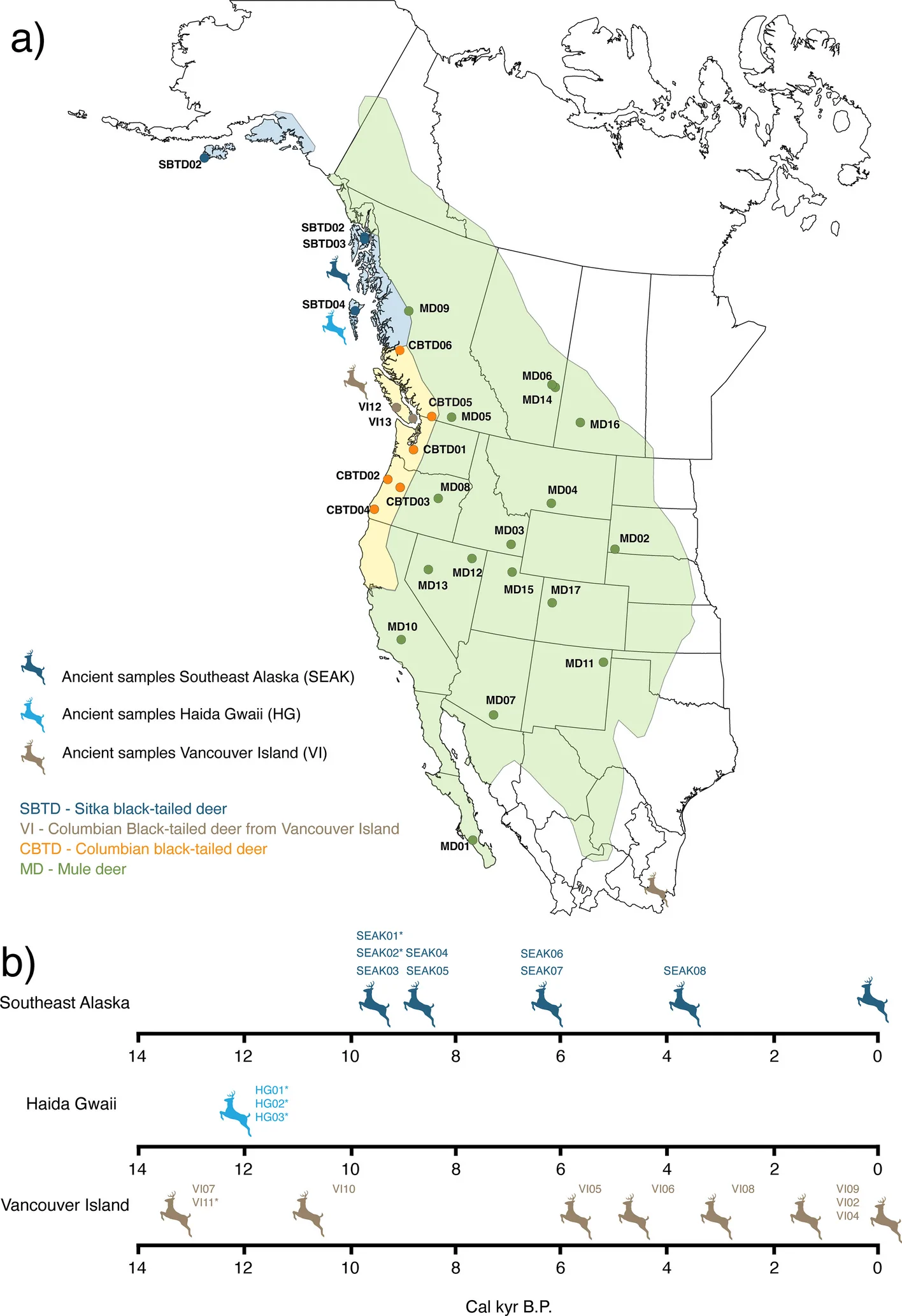
**a** Geographic location and sample acronyms of modern (circles) and ancient (deer silhouettes) samples. Circle colors indicate modern subspecies assignments and geographic locality: dark blue for Sitka black-tailed deer (SBTD), orange for Columbian black-tailed deer from Oregon, Washington, and British Columbia (CBTD), brown for Columbian black-tailed deer from Vancouver Island, and green for mule deer (MD). Ancient samples are represented by deer silhouettes at excavation locations, with dark blue representing Southeast Alaska (SEAK), light blue representing Haida Gwaii (HG), and brown representing Vancouver Island (VI). **b** Timeline showing the ages of ancient samples. In the text, the old-SEAK samples refer to Southeast Alaska samples SEAK01-SEAK05 (older than 8.5 cal kyr B.P), while the young-SEAK samples refer to SEAK06-SEAK08 (younger than 6 cal kyr B.P.). For samples marked with “*”, only mitogenome data were generated

We generated genomic data from 21 ancient black-tailed deer from Southeast Alaska (SEAK), Haida Gwaii (HG), and Vancouver Island (VI), in addition to 13 modern deer samples from throughout their distribution (Fig. 1, Additional file 1: Table S1). All ancient samples were radiocarbon-dated, with calibrated ages ranging from ~ 0.8 cal kyr B.P. to ~ 13.5 cal kyr B.P. The assembled ancient mitogenomes had an average depth of coverage ranging from 9.5 × to 432 × and were near-complete in width (> 90%; Additional file 1: Table S1). The ancient nuclear genome assemblies had an average read depth ranging from 0.01 × to 1.65 × and 1.6% to 71% of the genome covered, whereas modern samples had an average coverage of 3.8 × to 10x (depth) with 76% to 94% (width) of the genome covered (Table S2). Ancient nuclear and mitochondrial genomes showed the expected pattern of degraded ancient DNA, with an increased rate of cytosine deamination at the 5’-end of the reads, and an increased rate of guanine to adenine substitutions close to the 3'-end of the reads, confirming ancient DNA authenticity (Additional file 2: Figure S1 and Additional file 2: Figure S2). The increased rate of deamination was also observed in the error calculation performed in ANGSD [32] (Additional file 2: Figure S3). As a result, transitions were excluded and only transversion sites were included in subsequent analyses.

### Two divergent maternal lineages inhabited Southeast Alaska across the Holocene

**Fig. 2:**
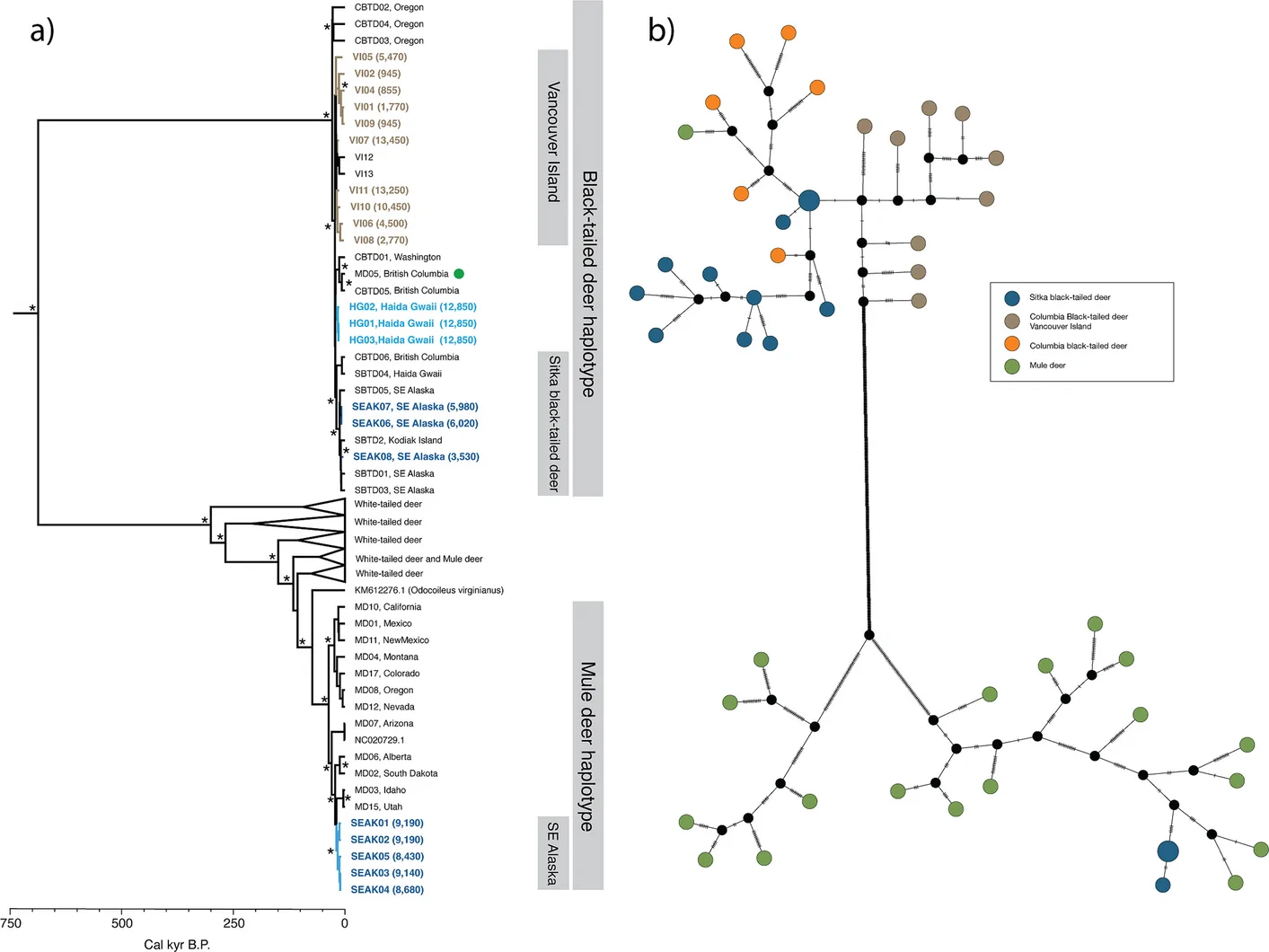
Phylogenetic relationships of *Odocoileus*. **a** A tip-calibrated Bayesian phylogenetic tree of *O. hemionus* and *O. virginianus* based on 99 near-complete (> 90% breadth coverage) mitogenomes. Colored branches and labels represent ancient deer: brown for Vancouver Island; light blue for Haida Gwaii; blue for ancient samples from SE Alaska. Stars indicate nodes supported in the Bayesian analyses (posterior probability > 0.95). A green dot is placed at MD5, a mule deer with a Columbian black-tailed deer haplotype. See Additional file 2: Figure S4 for the complete phylogenetic tree. **b** TCS haplotype network based on 49 *Odocoileus hemionus* mitogenomes. Circle sizes are proportional to the number of individuals within the haplotypes*.* The colors of individual samples follow subspecies assignment and geographic location (see Fig. 1)

Phylogenetic analyses of complete mitochondrial genomes confirmed previously reported groupings within modern *Odocoileus*: one clade comprising the black-tailed deer haplotype (*O. h. columbianus* and *O. h. sitkensis*), and another clade, where mule deer (*O. h. hemionus*) is nested within white-tailed deer (*O. virginianus*) haplotypes (Fig. 2a, see Additional file 2: Figure S4 for the complete tree). One sample, a mule deer collected from British Columbia (MD5), possessed the mitochondrial haplotype of Columbian black-tailed deer, although nuclear data confirmed its taxonomic identity as mule deer. The two major maternal lineages exhibited a deep split dating to ~ 670 cal kyr B.P. (95% HPD ~ 478–930 cal kyr B.P.). Notably, ancient SE Alaska samples (SEAK) older than 8.5 cal kyr B.P. (referred to as old-SEAK) coalesced ~ 16 cal kyr B.P. (95% HPD ~ 12–20 cal kyr B.P), and grouped with mule deer instead of black-tailed deer, while samples younger than 6 cal kyr B.P. (referred to as young-SEAK) grouped with modern Sitka black-tailed deer (acronym SBTD). This pattern suggests a shift in mitochondrial haplotype among Southeast Alaska deer between ~ 8.5 and 6 cal kyr B.P.

Within the black-tailed deer lineage, modern Columbian black-tailed deer (acronym CBTD) from Oregon (CBTD2, CBTD3, CBTD4) formed a sister group to a highly supported monophyletic group comprising samples from Washington, British Columbia, and Alaska (including most ancient samples) that diverged ~ 28 cal kyr B.P. (95% HPD ~ 19.5–38 cal kyr B.P.). The most recent common ancestor of the Washington, British Columbia, and Alaska clade was estimated at ~ 22 cal kyr B.P. (95% HPD ~ 16.5–29 cal kyr B.P.). This highly supported monophyletic clade suggests one main dispersal northwards when conditions became favorable. Despite the low genetic diversity inside this latter clade, revealed by short branches, two main groups emerged: one consisting of ancient and modern samples from Vancouver Island (VI), and the other comprising modern samples from Washington and mainland British Columbia (and the mule deer from British Columbia, MD5), as well as modern and ancient samples from Haida Gwaii and Alaska. Two samples, a Columbian black-tailed deer from Central British Columbia (CBTD6) and a modern Sitka black-tailed deer from Haida Gwaii (SBTD4), introduced from the British Columbia mainland, placed as sister group to a clade only encompassing Sitka black-tailed deer from Alaska (modern and ancient samples younger than 6 cal kyr B.P.). Those two clades shared a last common ancestor with Sitka black-tailed deer from Alaska ~ 18.4 kyr B.P. (95% HPD ~ 12.6–26.2 cal kyr B.P.). All samples from Southeast Alaska with a black-tailed deer haplotype (including those translocated to Kodiak Island) shared a last common ancestor ~ 11.6 cal kyr B.P. (95% HPD ~ 8–16 cal kyr B.P.).

The haplotype network revealed that the black-tailed deer and mule deer haplogroups differed by 624 substitutions, which correspond to a genetic difference of ~ 4.3% (Fig. 2b). Overall, the haplotype network captured similar relationships to the ones observed in the phylogenetic trees for *O. hemionus*, the main difference being the position of mainland Columbian black-tailed deer (CBTD) and samples from Vancouver Island (VI). The southern Columbian black-tailed deer (CBTD1-5) now clustered together and were nested inside black-tailed deer haplotype diversity*.* The Southeast Alaska group was separated from the ancient samples from the Haida Gwaii cluster by at least 14 mutations and from the samples from the central coastal British Columbia by at least 13 mutations.

### Postglacial differentiation of Columbian and Sitka black-tailed deer through isolation by distance

**Fig. 3:**
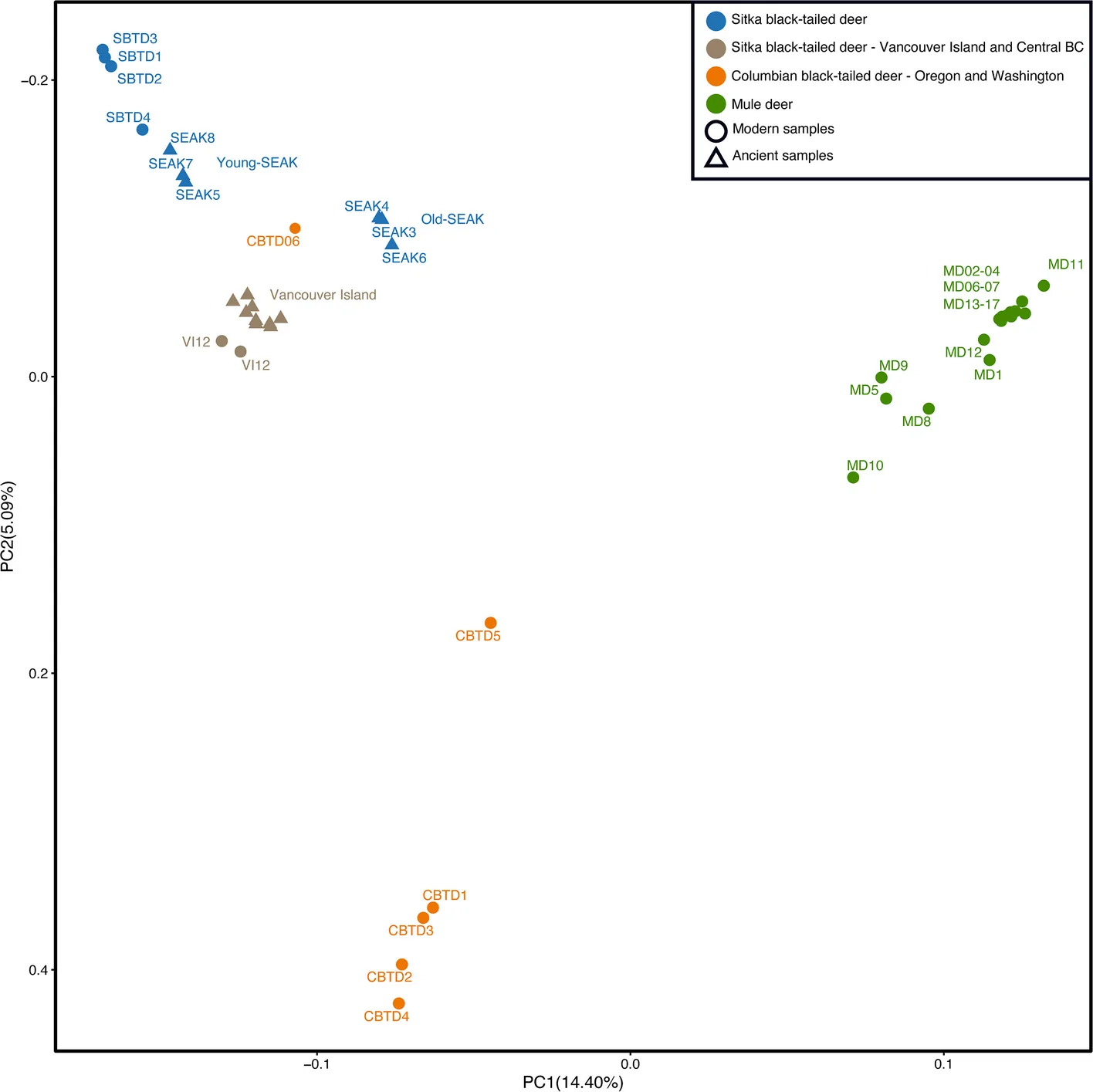
Population structure in *Odocoileus hemionus*. Principal component analysis (PCA) performed based on modern (shown with circles) and ancient (shown with triangles) individuals. The colors of individual samples follow subspecies assignment and geographic location (see Fig. 1)

To explore population structure from genome-wide deer data, pseudohaploid genotypes from both modern and ancient samples were called using ANGSD (-doHaploCall 1) [32]. Performing principal component analysis (PCA) using only transversions, placed modern deer samples into four main clusters: mule deer, Columbian black-tailed deer from mainland British Columbia and Vancouver Island, and Sitka black-tailed deer (Fig. 3). The only exceptions were two outlier Columbian black-tailed deer from southern and central British Columbia (CBTD5 and CBTD6), that placed in between these clusters and may be products of recent hybridization events. The majority of mule deer placed into a tight cluster, suggesting limited genetic diversity within the subspecies. However, four samples (MD05, MD08, MD09, MD10) placed closer to the Columbian black-tailed deer cluster along both PC1 and PC2, indicating potential historical and/or contemporary gene flow in areas where the subspecies come into contact [33].

Black-tailed deer displayed a non-perfect cline along PC1 and PC2, with modern Sitka black-tailed deer from SE Alaska at one extreme and Columbian black-tailed deer from Oregon at the other. In between these two clusters were samples from Haida Gwaii, central British Columbia, Vancouver Island, and southern coastal British Columbia, in this order. Hence, the cline mirrors the geographic distribution of black-tailed deer along the northwestern Pacific Coast, suggesting that the differentiation of the two black-tailed deer subspecies may be a consequence of isolation by distance.

### The two divergent Southeast Alaska maternal lineages are genetically Sitka black-tailed deer and closely related to Vancouver Island deer

All ancient samples from Southeast Alaska clustered with modern Sitka black-tailed deer in the principal component analysis (PCA), confirming their taxonomic identity. However, when compared to samples younger than 6 cal kyr B.P., ancient samples from the old-SEAK group (older than 8.5 cal kyr B.P.) were closer to the mule deer samples along PC1, supporting their mule deer mitochondrial haplotype. Ancient samples from Vancouver Island clustered close to modern individuals from the same region and positioned between the two black-tailed deer subspecies, although they placed closer to Sitka black-tailed deer than Columbian black-tailed deer along PC2.

### Results from exploring signatures of gene flow in further detail

**Fig. 4:**
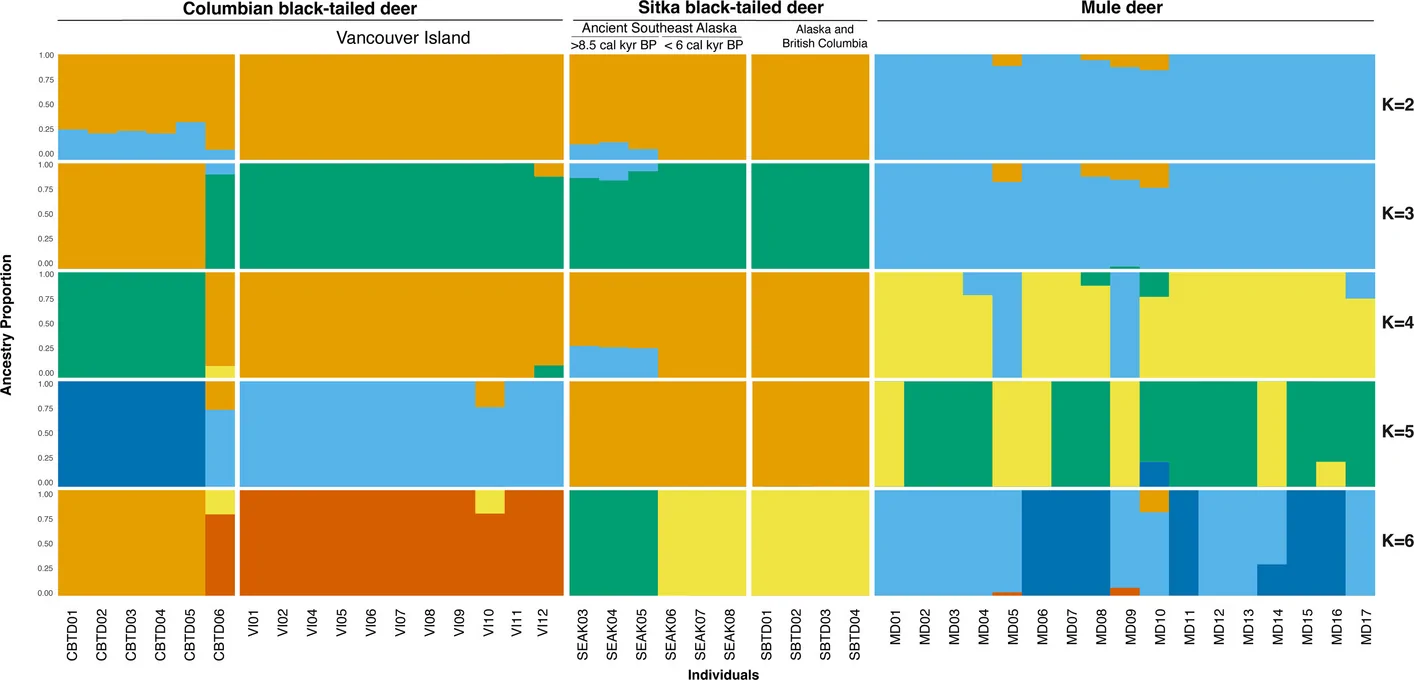
Ancestry proportions with 2–6 ancestral population clusters. K = 2 had the lowest cross-validation values. For all cross*-*validation values see Additional file 2: Figure S5 and the standard deviation of the bootstraps see Additional file 1: Table S3

To estimate ancestry in the studied samples, we applied the ADMIXTURE [34] method. Based on autosome transversion sites only, the cross-validation (–cv = 10) supported K = 2 (Additional file 2: Figure S5), distinguishing the mule deer and black-tailed deer subspecies (Fig. 4). All samples with pure black-tailed deer ancestry included deer from Vancouver Island, Haida Gwaii, and Alaska. Of the Columbian black-tailed deer that exhibited mixed ancestry, 72% (ranging from 64 to 75%) of the ancestry was shared with other black tailed deer populations (Southeast Alaska and Vancouver Island). All Southeast Alaska deer samples younger than 6 cal kyr B.P showed pure Sitka black-tailed deer ancestry, while the SEAK-old samples carried ~ 14% (ranging from 10 to 16%) mule deer ancestry.

The second-best model, K = 3, separated black-tailed deer into two ancestral groups: (1) southern Columbian black-tailed deer (Oregon and Washington) and (2) northern Columbian (British Columbia), Vancouver Island and Sitka black-tailed deer. This same pattern persisted until K = 5, where Sitka black-tailed deer separated from the Columbian black-tailed deer ancestral group, and mule deer showed additional internal structure. At K = 6, despite of a higher cross-validation value (0.48) when compared to previous K values, Sitka black-tailed deer divided further into two distinct ancestral groups: old-SEAK and young-SEAK, the latter including modern Sitka black-tailed deer.

**Fig. 5:**
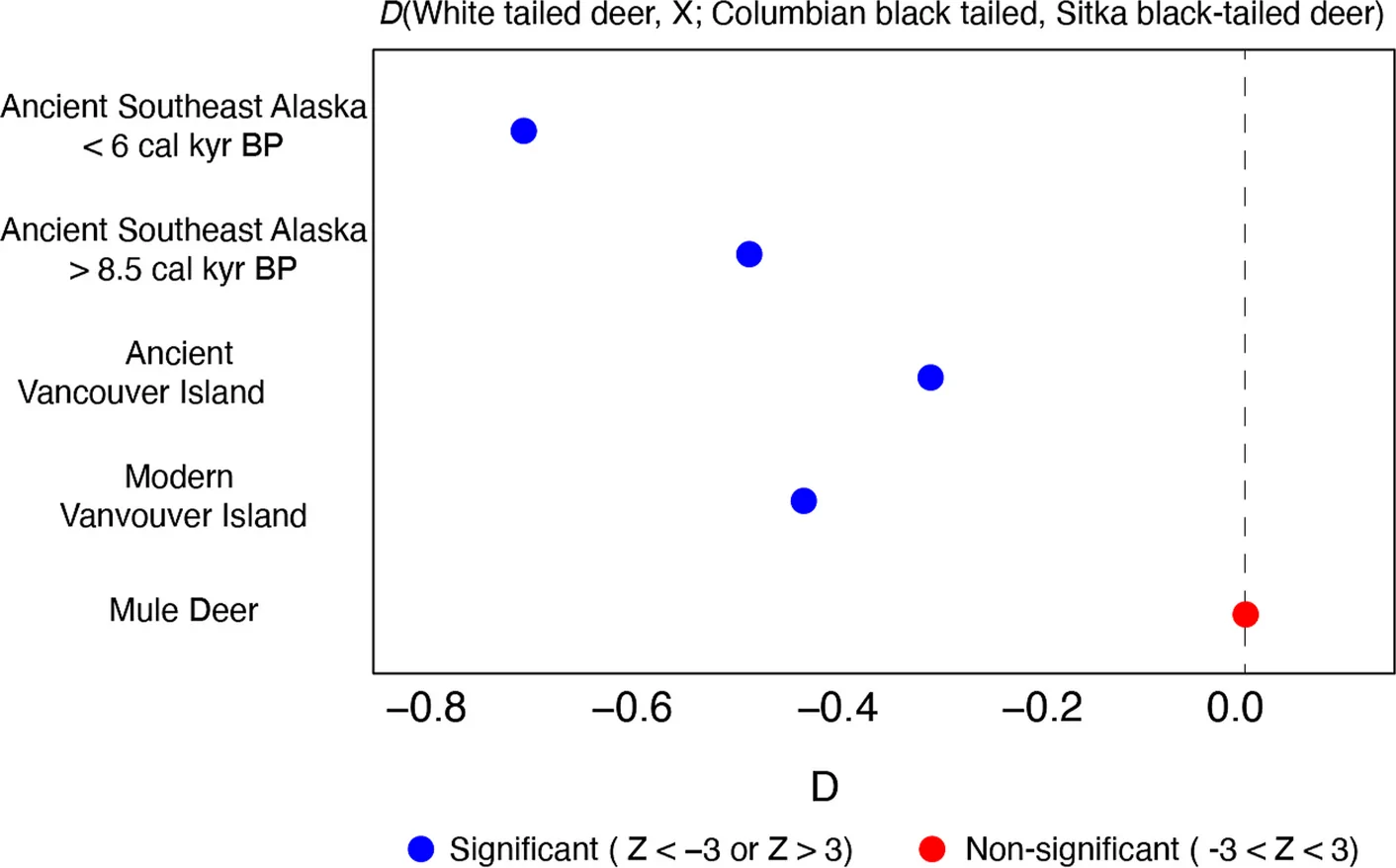
*D* statistics showing *D* values and their 95% confidence intervals from the combinations D(WTD, X; CBTD, SBTD), where X samples are listed along the y-axis: Ancient Southeast Alaska < 6 cal kyr BP (SEAK06, SEAK07, SEAK08), ancient Southeast Alaska > 8.5 cal kyr BP (SEAK03, SEAK04, SEAK05), ancient Vancouver Island (VI01, VI02, VI04, VI05, VI06, VI07, VI08, VI09, VI10), modern Vancouver Island (VI12, VI13), and mule deer (MD01-MD17)

We next evaluated the extent of drift shared among modern and ancient black-tailed deer populations along the Northwestern Pacific Coast using *D*-statistics, computing *D*(WTD, X; CBTD, SBTD), where *X* included samples from Vancouver Island (modern and ancient), Southeast Alaska (the two ancient groups, old and young), and mule deer. All tested combinations produced significantly negative *D* scores, indicating excess allele sharing between the tested samples and Sitka black-tailed deer, except in the case of mule deer, where *D* was not significantly different from zero (Fig. 5). Thus, both Vancouver Island and Southeast Alaska samples shared more alleles with Sitka than with Columbian black-tailed deer. Among ancient samples, *D* values decreased gradually from Vancouver Island to Southeast Alaska: young-SEAK shared the most alleles with Sitka black-tailed deer, Vancouver Island the least, while old-SEAK showed intermediate values, comparable to modern Vancouver Island deer.

To test for definitive evidence of admixture, we performed the *f*3 statistic (Additional file 2: Figure S6). Individuals were grouped into four source populations according to the results from principal component analyses: CBTD, VI (ancient and modern), SBTD, MD, and ancient individuals fom Southeast Alaska (young- and old-SEAK). Among the modern populations, only the southern Columbian black-tailed deer (CBTD) yielded significant values (Z < −3), when modern and young-SEAK Sitka black-tailed deer were sources with mule deer. This might indicate that Columbian black-tailed deer from its southern range originated via admixture of ancestral black-tailed deer and mule deer after the onset of deglaciation when populations of mule and Columbian black-tailed deer inhabiting opposite sides of the Cascade Range may have met. Ancient Vancouver Island samples, spanning several millennia, yielded highly negative values in most instances when Sitka or modern Columbian black-tailed deer were sources. This likely reflects strong identity by descent suggesting that the ancient Vancouver Island individuals represent an early coastal lineage ancestral to later black-tailed deer populations. However, these results should be interpreted with caution given the overall low coverage of this group. Interestingly, ancient samples from Southeast Alaska do not show evidence of admixture, indicating that the pattern observed for ancient Vancouver Island samples is not solely driven by their low coverage.

## Discussion

Our genomic analyses of ancient and modern black-tailed deer illuminate the dynamic postglacial history of northwestern North America, revealing how climate-driven landscape changes shaped population connectivity and lineage turnover. The identification of two divergent maternal lineages in Southeast Alaska, coupled with evidence of a turnover between ~ 8.5 and 6 cal kyr B.P., suggests that deglaciation facilitated northward colonization along the Pacific Coast, while isolation by distance drove subsequent differentiation between Sitka and Columbian black-tailed deer. Ancient samples from Vancouver Island and Southeast Alaska indicate that early coastal populations contributed substantially to the genetic makeup of modern Sitka black-tailed deer, highlighting the role of ice-age refugia and postglacial dispersal in shaping modern population structure. These findings provide a genomic framework linking glacial history, refugial dynamics, and contemporary population structure in a highly vagile herbivore.

### Deer in coastal British Columbia

In British Columbia, the fossil record reveals a gap in deer occurrence between approximately 13.5 and 5.5 cal kyr B.P., with only a single specimen recovered from this interval (VI10; ~ 10.4 cal kyr B.P.). Genetic data interpreted across this gap, however, indicate that deer likely persisted locally but remain unsampled. This pattern implies colonization of Vancouver Island by at least 13.5 cal kyr B.P. and survival through the Younger Dryas, a cooling period spanning ~ 12.8–11.5 cal kyr B.P. [35]. All sampled individuals from Vancouver Island and coastal British Columbia cluster closely with Sitka black-tailed deer from southeastern Alaska and share greater allele affinity with this northern lineage than with Columbian black-tailed deer from nearby Oregon and Washington. In contrast, southern Columbian black-tailed deer form a distinct genetic cluster, with only a single individual (CBTD05) showing intermediate ancestry consistent with its position at the northern edge of the southern distribution. This pattern is consistent with a north–south genetic gradient along the Pacific coast, likely shaped by rapid postglacial expansion and gene surfing along the leading edge of colonization [36]. Under this scenario, populations along the British Columbia coast and Vancouver Island represent part of a continuous northern coastal lineage closely related to Sitka black-tailed deer, whereas southern Columbian black-tailed deer reflect a more differentiated southern lineage.

Although Vancouver Island deer are traditionally classified as Columbian black-tailed deer based on morphology and historical taxonomy, their genomic affinity suggests closer evolutionary continuity with Sitka black-tailed deer populations. This discrepancy highlights the need for integrative taxonomic reassessment combining morphology, fossils, and genomic data to resolve coastal population structure in *Odocoileus hemionus*.

**Fig. 6:**
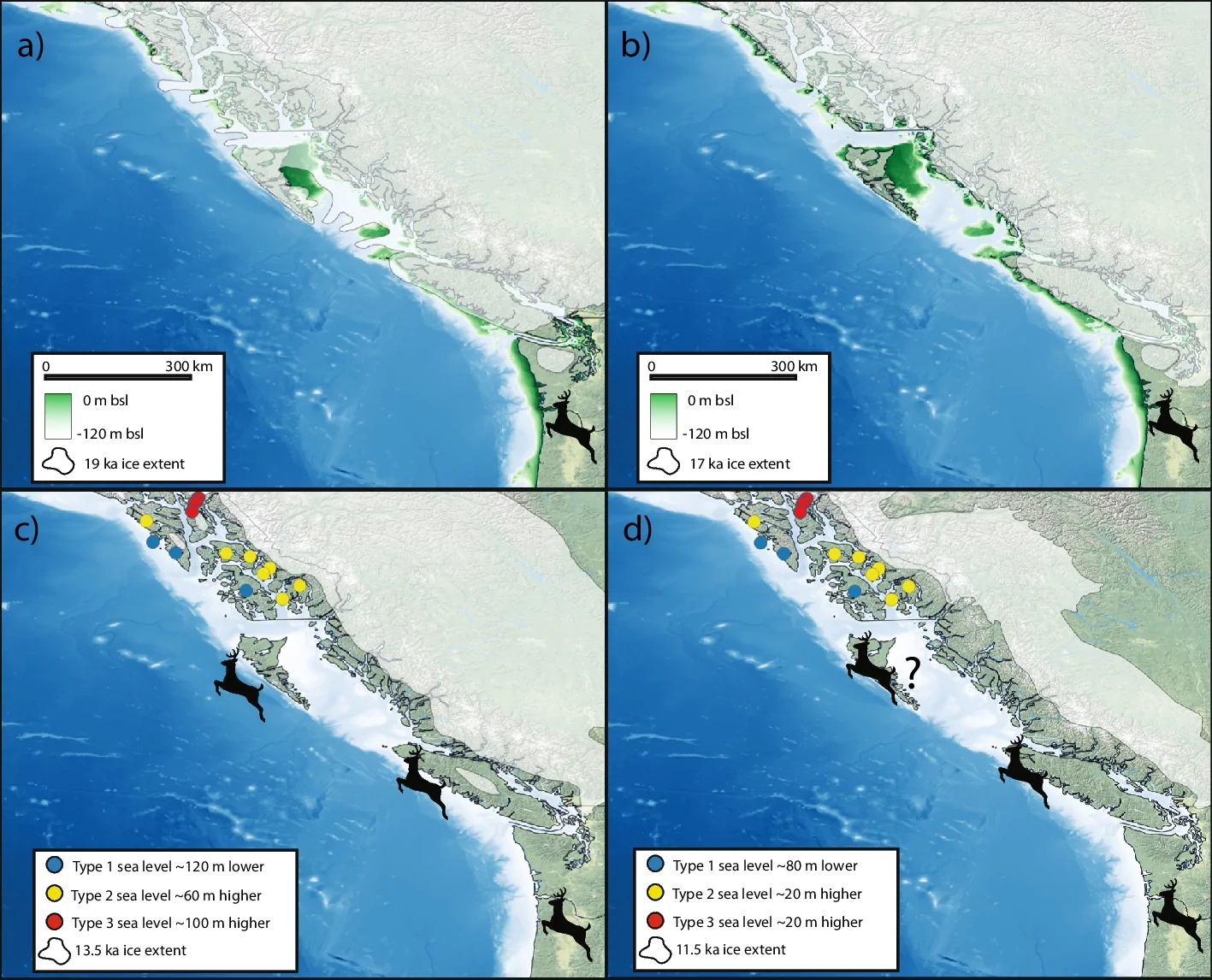
Maps of the Northwest Pacific Coast showing the extent of the Cordilleran Ice Sheet and deglaciation over time (based on data from Dalton et al. [38]): **a** 19 thousand year ago (ka), **b** 17 ka, **c** 13.5 ka, and **d** 11.5 ka. Bathymetric data from Jakobsson et al. [39] and Baichtal et al. [40]. Deer silhouettes in Oregon and Washington in the time period 19–17 ka indicate survival in refugia south of the ice sheet, and deer along the coast in later time intervals indicate their postglacial appearance in the fossil record. Deer silhouettes with question marks in the Alexander Archipelago indicate uncertainty about the timing of their arrival in the region

Building on this regional pattern, ancient samples from Haida Gwaii offer additional insights into early coastal colonization. Although no nuclear data were available, mitochondrial genomes from these specimens carried black-tailed deer haplotypes, forming a monophyletic clade sister to modern populations from Haida Gwaii, central British Columbia, and Alaska (ancient and modern), consistent with a single major northward migration wave. The fossil record indicates only a brief occupation of deer in Haida Gwaii, with lower sea levels around 13.5 cal kyr B.P. likely facilitating initial colonization across the exposed Hecate Strait (Fig. 6) [37, 38]. Deer disappeared from the Haida Gwaii fossil record ~ 12.8 cal kyr B.P., coinciding with the onset of the Younger Dryas period and the contemporaneous extirpation of brown bears (*Ursus arctos*), and the islands remained unoccupied by black-tailed deer until the early twentieth century, when a population was introduced from the British Columbia mainland [16, 27, 37]. This disappearance marks the last fossil evidence of black-tailed deer along the mid-coast before their eventual establishment farther north, setting the stage for their later arrival in Southeast Alaska.

### The arrival of deer in Southeast Alaska

Southeast Alaska marks the northernmost native distribution of Sitka black-tailed deer, with individuals from the Alexander Archipelago later introduced to the Kodiak Archipelago in the early twentieth century [28, 30]. All samples from Southeast Alaska younger than 6 cal kyr B.P., including the extant population, shared a last common matrilineal ancestor ~ 12 cal kyr B.P., suggesting colonization followed by isolation in the Alexander Archipelago. However, older Southeast Alaska samples (~ 9.2–8.5 cal kyr B.P.) carried a mule deer mitochondrial haplotype, indicating this timing of initial deer colonization may be underestimated.

Paleoclimate evidence suggests that this time interval coincided with a period when the Northwest Pacific Coast provided a viable human migration corridor between Beringia and regions south of the ice sheets [41]. Considering the sea-level history (Fig. 6), subfossil record, and molecular divergence time estimates, the interval between 13 and 11.7 cal kyr B.P.) likely offered the most favorable conditions for northward expansion and colonization of the Alexander Archipelago. Subsequent sea-level rise, reaching up to 20 m above present levels along the outer coast [40], may have later restricted dispersal and contributed to population isolation.

Two distinct matrilines of Sitka black-tailed deer are evident in southeastern Alaska. Ancient specimens dated to ~ 9.2–8.5 cal kyr B.P. carry mule deer mitochondrial haplotypes, whereas Sitka black-tailed deer haplotypes are only detected in younger samples (< 6 cal kyr B.P.). This temporal pattern suggests a transient phase of mitochondrial introgression in early postglacial populations, followed by replacement or dilution of mule deer maternal lineages. The timing and geographic context of this introgression remain uncertain, but it may reflect secondary contact during northward postglacial range expansion. By the time Sitka black-tailed deer became established in Southeast Alaska (~ 13–11.7 cal kyr B.P.), the ice-free corridor between the Cordilleran and Laurentide ice sheets was ecologically viable [4, 5], potentially facilitating dispersal of mule deer into northern regions prior to or concurrent with Sitka black-tailed deer expansion. However, direct evidence for their presence in the Alexander Archipelago remains lacking. Similar patterns of mitochondrial capture and lineage replacement have been documented in other mammals occupying glacially dynamic landscapes, including brown bears in the Alexander Archipelago [42].

A gap in the Southeast Alaska deer fossil record between ~ 8.5 and 6 cal kyr B.P. coincides with the apparent matrilineal turnover in which Sitka black-tailed deer haplotypes replaced mule deer haplotypes. Although Sitka black-tailed deer haplotypes are not observed prior to 6 cal kyr B.P., the limited sample size and incomplete fossil record leave open the possibility that both haplotypes co-occurred within the Alexander Archipelago. One of the oldest specimens (SEAK3) exhibits unusual antler and mandibular morphology [14], suggesting that the early Southeast Alaska deer population may have been morphologically distinct and potentially localized. Importantly, fossils of other mammals, including American black bear (*Ursus americanus*) and otter (*Lontra canadensis*), are present during this interval [14], indicating that the absence of deer fossils is unlikely to reflect a general preservation bias. Brown bears also disappeared from the southern islands during approximately the same period [14, 16], suggesting broader faunal change during the middle Holocene.

The apparent deer decline and subsequent haplotype turnover occurred during a period of climatic and environmental reorganization in Southeast Alaska. Regional paleoclimate records indicate relatively warm early-Holocene conditions, followed by Neoglacial cooling, glacier expansion, and the establishment of modern vegetation communities after ~ 5–6 ka [43, 44]. However, the timing and magnitude of Holocene climate change in Southeast Alaska remain debated, with recent speleothem studies suggesting a more complex temperature history than previously recognized [45, 46]. Although the ecological impacts of these climatic transitions remain poorly understood, the temporal coincidence of deer population decline, brown bear disappearance, and major environmental change suggests that climatic and ecological reorganization may have contributed to the local extirpation of early deer populations, followed by recolonization by populations carrying Sitka black-tailed deer haplotypes.

### Asynchronous deglaciation and northward postglacial dispersal of black-tailed deer

The combined fossil, morphological, and genomic evidence from Southeast Alaska and coastal British Columbia points to a dynamic postglacial history characterized by range shifts, local extirpation, lineage turnover, and repeated colonization events. To place these regional patterns in a broader evolutionary context, we interpret our genetic findings alongside paleoclimatic and geological reconstructions to assess how asynchronous deglaciation along the Northwest Pacific Coast shaped the postglacial expansion of black-tailed deer.

Previous analyses of nuclear microsatellites and mitochondrial DNA sequence data showed that modern black-tailed deer exhibit their highest genetic diversity in Oregon and Washington, comparable to mule deer levels, whereas the lowest diversity occurs in the Alexander Archipelago [19, 23]. This gradient has been interpreted as evidence of survival in a common glacial refugium during the Last Glacial Maximum, followed by postglacial northward expansion and loss of genetic diversity at the range margins. Consistent with this scenario, our paleogenomic results indicate a strong genetic affinity between Vancouver Island, British Columbia and Southeast Alaska populations dating back at least 13.5 cal kyr B.P., supporting a primary northward dispersal from a southern refugium. Based on mitochondrial DNA divergence estimates, the Canadian and Alaskan group coalesced ~ 18 cal kyr B.P. and shared a last common ancestor with samples from Oregon ~ 22 cal kyr B.P. These timelines suggest that initial northward dispersal and population divergence could have begun during the terminal phases of the LGM, also overlapping with periods where the Northwest Pacific coastal migration corridor was viable [41]. However, presence of the Cordilleran Ice Sheet along the coast could have impacted such northward dispersal.

The Cordilleran Ice Sheet reached its maximum southwestern extent in the Puget Lowland around 17 cal kyr B.P., followed by a rapid retreat [47, 48]. In contrast, deglaciation occurred earlier along the outer coast, with Vancouver Island and the Haida Gwaii Archipelago becoming ice-free as early as 18.5–17 cal kyr B.P. (Fig. 6) [7, 8, 37], whereas Southeast Alaska remained glaciated until ~ 16–15 cal kyr B.P. [1, 9]. This asynchronous deglaciation along the Northwest Pacific Coast may have delayed a postglacial expansion into northern regions, creating transient coastal refugia that may have served as stepping stones for northward migration once ice-free conditions expanded. Lowered sea levels, beginning by at least 14.5 cal kyr B.P [40], could have exposed broader coastal plains and ecologically favorable coastal habitats, offering temporary ice-free refuges surrounded by an unsuitable glacial landscape. Our paleogenetic evidence is consistent with such asynchronous northward postglacial dispersal of black-tailed deer by the end of the Last Glacial Maximum. The observed allele sharing between Columbian and Sitka black-tailed deer suggests transient isolation of coastal populations during this period through founder effect or gene surfing as populations advanced northward into Southeast Alaska and the Alexander Archipelago. Consistent with this interpretation, *f*_3_ statistics suggest the presence of an early lineage on Vancouver Island at the end of the Pleistocene, which may reflect retained ancestral variation or early postglacial connectivity among coastal populations.

While the temporal correspondence between major climatic transitions and inferred demographic changes is consistent with a climate-mediated model of postglacial expansion, we acknowledge that causality cannot be established directly from the available data. Climatic change, geographic barriers, and isolation-by-distance processes likely acted in concert to shape population structure across the region, and some observed genetic patterns may be explained by multiple, non-mutually exclusive demographic scenarios. Although our findings support a primary role for postglacial habitat availability and coastal dispersal opportunities in driving recolonization, alternative explanations involving stochastic founder effects, localized population persistence, and subsequent gene flow cannot be entirely excluded. Future analyses incorporating larger temporal and geographic sampling, as well as formal demographic model testing, will help further disentangle the relative contributions of these processes.

## Conclusions

By integrating ancient genomic and fossil evidence, we reveal new insights into the divergence and postglacial history of Columbian (*O. h. columbianus*) and Sitka (*O. h. sitkensis*) black-tailed deer, illuminating evolutionary processes that have long remained uncertain. The combined evidence suggests that a separate northern refugium for black-tailed deer during the Last Glacial Maximum (LGM) is unlikely. Instead, genetic patterns suggest that both subspecies persisted in a common refugium south of the Cordilleran Ice Sheet, and divergence of Sitka black-tailed deer from southern populations began near the end of the LGM. Black-tailed and mule deer appear to have been isolated in separate refugia on opposite sides of the Cascade Range [23], and following deglaciation, northward expansion from these distinct southern refugia likely resulted in secondary contact between diverging lineages. Such hybrid zones, observed today [33], may explain the presence of mule deer haplotypes among early colonizing populations in Southeast Alaska. The asynchronous retreat of the Cordilleran Ice Sheet and fluctuating sea levels along the Northwest Pacific Coast likely facilitated a series of dispersal pulses and localized isolations, shaping the present-day genetic and morphological diversity of *O. hemionus*.

At present, the geographic range of Columbian black-tailed deer extends north to central British Columbia (Fig. 1) [49]. However, given the genetic similarity of individuals from Vancouver Island, and potentially central British Columbia, with Sitka black-tailed deer, our results indicate that the current subspecies boundaries and distribution of black-tailed deer warrant further evaluation.

Taken together, these findings illustrate how glacial cycles and asynchronous deglaciation along the Northwest Pacific Coast shaped not only the distribution and genetic structure of black-tailed deer but also broader patterns of diversification among coastal species. By integrating ancient DNA with paleontological and paleoenvironmental data, this study highlights the power of interdisciplinary approaches to disentangle complex postglacial histories and refine our understanding of refugial dynamics, gene flow, and adaptation in northern ecosystems.

## Methods

### Sampling and radiocarbon dating

Twenty-one subfossil deer samples from the Alexander Archipelago, Haida Gwaii, and Vancouver Island were analyzed (Additional file 1: Table S1; Fig. 1). All subfossils from Southeast Alaska had been previously identified based on morphology, with most of the samples classified as deer without a subspecies assignment, and two samples identified as either deer or caribou [14]. Of the three samples from Haida Gwaii, two were morphologically identified as ungulate, and one as deer [37]. All Vancouver Island samples had been morphologically identified as deer [37]. We also generated whole-genome data from six modern black-tailed deer from Alaska, British Columbia, and Oregon, and seven modern mule deer from different parts of their North American distribution (Additional file 1:Table S2; Fig. 1). In addition to the newly generated data, we downloaded complete mitogenome data and whole genome data available in NCBI (Additional file 1: Table S2).

All ancient samples analyzed in this study had been previously ^14^C radiocarbon-dated based on bone collagen [8, 14, 37]. The radiocarbon dates were calibrated using the IntCal20 calibration curve in OcCal v4.3 [50, 51], reporting 2-sigma (Additional file 1: Table S1).

### DNA extraction, PCR amplification, mitochondrial enrichment, and shotgun sequencing

DNA from subfossils was extracted in a dedicated cleanroom facility at the University at Buffalo, separated from any processing of modern samples. Ancient DNA extractions followed the protocol described in [52] and the modifications in [15]. DNA from modern samples was extracted using either a salt extraction procedure [53, 54] or a DNeasy Blood and Tissue kit (Qiagen).

To obtain an initial genetic-based taxonomic identification of the samples, PCR reactions were performed, either adding 21μL H_2_O, 0.4 μM of each forward and reverse primer, and 2 μL of extracted genomic DNA to each GE Illustra PuReTaq Ready-To-Go PCR bead (GE Healthcare) or by adding 2.5 µl of 10 × PCR buffer (Applied Biosystems, USA), 2.5 mM of each dNTP (Applied Biosystems), 25 mM of MgCl_2_ (Applied Biosystems), 5–10 U µl^−1^ of AmpliTaq Gold DNA polymerase (Applied Biosystems), 10 µM each of the forward and reverse primers, 2 µl of the genomic DNA and 17.4 µl of H_2_O. Because all the samples had been identified as deer/ungulate, we designed taxon-specific primers and amplified two regions of mitochondrial DNA *cytochrome b* (using primer pairs UB285F 5’-ATCACAAATCTCCTCTCAGC-3’/UB286R 5’-TGGACTATAGCRAGTGCTGCGA-3’ and UB287F 5’-CCAGCAAACCCACTCAAYAC-3’/UB288R 5’-ACTCCTTAGTTTATTTGGA-3’, respectively), and one region of the control region (UB289F 5’-TACCATCCCTGAAACCA-3’/UB290R 5’-TGGCCCTGAAGAAAGAACCAG-3’, respectively). PCR products were purified using a MinElute PCR Purification kit (Qiagen), and Sanger sequenced directly using the same primers as in the PCR reaction.

To assemble complete mitochondrial genomes of ancient samples, library preparation and mitochondrial DNA target enrichment were performed by Arbor Daicel Biosciences (http://www.arborbiosci.com). DNA from twelve ancient samples was target-enriched using a bait panel developed from white-tailed deer (NCBI accession NC-015247). Four of the sample libraries followed the standard myBaits v. 3.0 protocol, with equal masses of libraries pooled, bead-templated, and sequenced on the Ion Proton platform. Following sequencing, reads were demultiplexed, quality-trimmed, and filtered using the default settings on the Ion Torrent suite v. 4.4.3. Following sequencing, reads were demultiplexed, quality-trimmed and filtered using the default settings on the Ion Torrent suite v. 4.4.3. For the remaining eight samples, TruSeq dual-barcoded libraries were prepared without sonication, using the blunt-end ligation module from the NEBNext Fast DNA library preparation kit (New England BioLabs) with an extended double-time treatment and blunt-end adapters synthesized by Arbor Biosciences and paired-end sequenced on an Illumina HiSeqX platform.

Additionally, low-depth Illumina shotgun sequencing was performed for 15 ancient deer samples (six samples from Southeast Alaska and nine from Vancouver Island), in addition to 13 modern *O. hemionus* individuals. Single-stranded libraries from ancient samples were prepared using ssDNA2 [55, 56], and double-stranded libraries from modern samples were prepared using the NEBNext Fast DNA library preparation kit (New England BioLabs). All libraries were sequenced on an Illumina HiSeqX platform. One sample (SBTD5) was sequenced on an Illumina MiSeq following the manufacturer's recommended protocols, conducted at the U.S. Geological Survey's Alaska Science Center, Anchorage, Alaska.

### Genome mapping, assembly, and DNA degradation assessment

Due to the substantial mitochondrial divergence between mule and black-tailed deer, coupled with the absence of publicly available Sitka black-tailed deer mitogenomes when initiating this study, we assembled a de novo mitochondrial genome from a Sitka black-tailed deer harvested from Southeast Alaska (SBTD5). This assembly was achieved using NOVOPlasty [57], a tool designed for the de novo assembly of organellar genomes. To assemble mitogenomes from ancient samples, Illumina adapters were initially trimmed with AdapterRemoval v. 2.3.2 [58], reads shorter than 30 bp were discarded, and paired-end reads with a minimum overlap of 11 base pairs were merged; two base pairs were trimmed from the 5’ and 3’ ends. Ancient samples were aligned separately against reference mitogenomes (our own black-tailed deer mitogenome SBTD05) and mule deer NC_020729.1). Reads were aligned to the reference using the bwa-aln algorithm v. 0.7.17 [59], with maximum edited distance (-n) set to 0.01, the maximum number of gap open (-o) to 2, and the seed (-l) to 16,500. PCR duplicates were removed using the MarkDuplicates tool in Picard v. 2.25.0 [60] using the lenient validation stringency. The consensus was called using samtools mpileup [61] and the default settings. Mapping statistics were calculated with BEDTools v. 2.30.0 [62] Reads from modern samples were mapped together using BWA-mem [63] under the default settings. PCR duplicate removal, consensus calling, and mapping statistics were performed following the same pipeline described above. To assemble the nuclear genome, the same pipeline as above was used, with the reads aligned against the white-tailed deer reference genome Ovir.te_1.0 [GCF_002102435.1].

To analyze the damage pattern and assess the DNA authenticity of ancient samples, we repeated the same mapping pipeline for ancient samples as described above; however, we omitted the trimming step of two base pairs from the 5’ and 3’ ends. We used mapDamage2 v. 2.0.8 [64], which uses an approximate Bayesian estimation of the damage patterns, to assess DNA degradation patterns of the reference-mapped assemblies (mitochondrial and nuclear genomes) of each ancient deer individual (Additional file 2: Figure S1 and Additional file 2: Figure S2). We calculated the error rate of the ancient genomes using ANGSD [32], doerror 1 (Additional file 2: Figure S3).

### Mitochondrial genome analyses

In addition to the 34 newly assembled mitogenomes for this study, we downloaded complete mitogenomes of modern deer species from the tribe Odocoileini: *Odocoileus virginianus* (8), *O. hemionus hemionus* (2) from the National Center for Biotechnology Information (NCBI) GenBank database (Additional file 2: Figure S3). Reads from an additional two *O. h. sitkensis*, four *O. h. columbianus*, ten *O. h. hemionus*, and 18 *O. virginianus* were downloaded from the NCBI Sequence Reads Archive (SRA; Additional file 1: Table S2), and the mitogenomes were assembled following the same pipeline described above for modern samples. The total 68 sequences were aligned in MAFFT [65], with a manual inspection in Geneious v. 2023.0.4 to remove tandem repeats from the control region. To exclude unaligned regions, we performed Gblocks in SeaView v. 5.0.5 [66]. To obtain estimated divergence data, BEAST v. 2.7.1 [67], the subsitution model was average across the mitogenome by bmodeltest [67] and constant-size coalescent model. Trees were sampled every 1,000 states across two independent runs (70 million states each) and combined using LogCombiner. Convergence was assessed with Tracer (ESS > 200) [68]. Age calibration of divergence time estimation was performed by adding tip dates using calibrated radiocarbon dates from ancient samples. An additional dataset composed only of *Odocoileus hemionus* was used to generate a TCS parsimony network using PopART [69].

### Whole genome analyses

In addition to the genome data from 15 ancient and 13 modern samples generated in this study, 32 genomes, including those from *O. virginianus*, were downloaded from public repositories (Additional file 1: Table S2). SNP variation was calculated using ANGSD [32] via pseudo-haploidization to remove low-confidence sites (-baq 0 -remove_bads 1 -uniqueOnly 1 -minMapQ 25 -minQ 30 -rmTrans 1 -rmTriallelic 1e-4 -SNP_pval 1e-16 -C 50 -baq 0 -setminind 1 -maf 0.05) -dohaplo 1, only keeping sites present in 25% of the individuals, and only autosomes were used. The dataset was converted to PLINK format [70] using HaploToPlink in ANGSD [32]. All subsequent analyses were performed based on pseudohaploid data and transversions only. Two distinct datasets were created for downstream genomic analyses (Additional file 1: Table S3): the first, DS1 (*N* = 61), included all modern and ancient individuals, and white-tailed deer as an outgroup (total 16,122,168 transversion SNPs), and the second, DS2 (*N* = 44), composed only of modern and ancient *O. hemionus* (total 3,956,247 transversion SNPs with 25% missing data allowed).

To visualize population structure among modern and ancient *O. hemionus* (DS2), we performed principal component analyses (PCA) using *smartpca *[71] with the inbreed, auto-shrink option.

Unsupervised admixture proportions were estimated using ADMIXTURE [34] on DS2 testing K values from 2 to 6. Confidence intervals for ancestry proportions were calculated based on 100 pseudo-replicate bootstraps (-B100; see Additional file 1: Table S3 for standard deviation).

To test for more definitive evidence of admixture, we next performed the *f*_3_ statistic on the dataset DS1 using AdmixTools v. 7.0.1 with the inbreed and useallsnps on [72]. We computed *f*_3_(C; A, B), where C is the individual being tested (target), and A and B are the sources. A *Z* value below −3 indicated that the target is admixed or has a close genetic relationship (population sharing) with one of the sources due to the presence of segments that are identical by descent [42]. We calculated *f*_3_ values from the following populations Ancient Southeast Alaska < 6 kya BP (SEAK06, SEAK07, SEAK08), Ancient Southeast Alaska > 8.5 cal kyr BP (SEAK03, SEAK04, SEAK05), Ancient Vancouver Island (VI01, VI02, VI04, VI05, VI06, VI07, VI08, VI09, VI10), Modern Vancouver Island (VI12, VI13), and Mule deer (MD01-MD17). *Z* values were converted into *p*-values and corrected using p.adjust and FDR methods [73, 74] and then reconverted to *Z* values.

To further estimate the extent of gene flow, we performed a four-sample test of admixture, *D-*statistic (ABBA-BABA), from dataset DS1, using AdmixTools [75] with inbreed and useallsnps on. The same populations defined above were used. Unadmixed white-tailed deer was used as an outgroup, based on [25]*.* To test whether the ancient samples and modern samples from British Columbia were more closely related to Sitka or Columbian black-tailed deer, we computed *D* (CBTD, SBTD; X, WTD) for individuals, where CBTD represents a Columbian black-tailed deer individual from Oregon, Washington, and southern British Columbia, SBTD a modern Sitka black-tailed deer, WTD a white-tailed deer individual, and X the sample that is being tested. A negative result indicated gene flow between the sample tested and Sitka black-tailed deer, while a negative result indicated gene flow with Columbian black-tailed deer.

### Deglaciation and exposure of continental shelves along the Northwest Pacific Coast

To determine periods during the Late Pleistocene that could have been viable for deer migration based on ice sheet retreat, bathymetric data, and sea level fluctuation, we downloaded the North American Deglaciation Isochrones (NADI-1) database from 19, 17, 13.5, and 11.5 cal kyr B.P. from [38], and incorporated bathymetric data from the Northwest Pacific Coast [39] and sea level history from the Alexander Archipelago [40].

## Supplementary Information

Additional file 1: Supplementary Files.

Additional file 2: Supplementary Figures.

Additional file 3.

## Data Availability

The mitochondrial genome sequences generated in this study are available in File S1. Raw reads for ancient and modern deer are deposited in the NCBI Sequence Read Archive (SRA) with the BioProject no. PRJNA1358933 [76]. Previous published raw reads are available at SRA: PRJNA830519 [25], SRS3444430 [77], SRS10021489 [78], and SRS2795074 [79]. All custom analysis scripts used in this study are publicly available on GitHub (https://github.com/flavioascoelho/SitkaBlackTailedDeerAnalyses) [80] and achieved on Zenodo (https://doi.org/10.5281/zenodo.21461009) [81].
