# Supplementary material for: Ancient genomes of Sitka black-tailed deer show evidence for early postglacial dispersal along the Pacific Northwest Coast of North America
Source: Genome Biol. 2026 Aug 5;27:341. doi: 10.1186/s13059-026-04233-5 (PMC13726212; doi:10.1186/s13059-026-04233-5)
Supplement: Supplementary file 1 [file 13059_2026_4233_MOESM2_ESM.pdf]

Ancient genomes of Sitka black-tailed deer show evidence for early postglacial dispersal along the Pacific Northwest Coast of North America

Flavio Augusto da Silva Coelho[1,2*], Crystal M. Tomlin[1], Karlee K. Prince[3], Duncan McLaren[4], Daryl Fedje[4], Emily Latch[5], James R. Heffelfinger[6], James F. Baichtal[7], Sandra L. Talbot[8], Timothy H. Heaton[9], Charlotte Lindqvist[1*]

[1] Department of Biological Sciences, University at Buffalo, Buffalo, New York, USA
[2] Biology Department, Trent University, Peterborough, Canada
[3]Department of Earth Sciences, University at Buffalo, Buffalo, New York, USA
[4] Hakai Institute, Heriot Bay, British Columbia, Canada
[5] Department of Biological Sciences, University of Wisconsin – Milwaukee, Milwaukee, Wisconsin, USA
[6] Terrestrial Wildlife Branch, Arizona Game and Fish Department, Phoenix, AZ, USA.
[7] Blackpowder Consulting, Alaska, USA
[8] Far Northwestern Institute of Art and Science, Anchorage, Alaska, USA
[9] Department of Earth Sciences, University of South Dakota, Vermillion, South Dakota, USA

[*]Correspondance:
flaviodasilvacoelho@trentu.ca;
cl243@buffalo.edu

**Additional File 2:Figure S1** DNA damage profiles showing patterns of fragmentation and misincorporation for the ancient mitogenomes, generated using mapDamage2	**3**
**Additional File 2: Figure S2** DNA damage profiles showing patterns of fragmentation and misincorporation for the ancient whole-genomes, generated using mapDamage2	25
**Additional File 2: Figure S3**. Error rates of ancient samples estimated with ANGSD	39
**Additional File 2: Figure S4**. A tip calibrated Bayesian phylogenetic tree constructed with BEAST2, showing the complete tree Figure 2.	40
**Additional File 2: Figure S5**. Admixture cross validation values (--cv=10) from K2 to K6.	41
**Additional File 2: Figure S6** *f3*-statistics plots showing all possible combinations for modern and ancient *Odocoileus hemionus* populations	42

Additional Files:

Additional File 1: Table S1. Voucher information and sequencing statistics for deer subfossil samples analyzed in this study.

Additional File 1:Table S2. Sample information for modern deer assembled for this study.

Additional File 1: TableS3: Standard deviation values from ADMIXTURE bootstrap replicates (-B 100).

Additional File 2: Figure S1. DNA damage profiles showing patterns of fragmentation and the ancient mitogenomes, generated using mapDamage2[61]]. All samples showed the expected ancient DNA damage pattern with an increased rate of cytosine deamination at the 5'-end of the reads and an increased rate of guanine to adenine substitution close to the 3'-end of the reads, as expected for damage patterns of ancient DNA. The four upper plots represent the frequency of each base substitutions. The two bottom plots show all possible misincorporations, red represent C to T; blue G to A; grey all other substitutions; orange shows soft-clipped bases; green indicates deletions relative to the reference, and purple indicates insertions. X-axes indicate its nucleotide position relative to 5' or 3' (left shows substitutions from the 5', and right from the 3').

Additional File 3: Figure S2. DNA damage profiles showing patterns of fragmentation and the ancient whole-genomes, generated using mapDamage2 [61]. All samples showed the expected ancient DNA damage pattern with an increased rate of cytosine deamination at the 5'-end of the reads and an increased rate of guanine to adenine substitution close to the 3'-end of the reads, as expected for damage patterns of ancient DNA. The four upper plots represent the frequency of each base substitutions. The two bottom plots show all possible misincorporations, red represent C to T; blue G to A; grey all other substitutions; orange shows soft-clipped bases; green indicates deletions relative to the reference, and purple indicates insertions. X-axes indicate its nucleotide position relative to 5' or 3' (left shows substitutions from the 5', and right from the 3').

Additional File 4: Figure S3. Error rates of ancient samples estimated with ANGSD (-doerror 1).

Additional File 5: Figure S4. A tip calibrated Bayesian phylogenetic tree constructed with BEAST2, showing the complete tree Figure 2. Nodes values represent posterior probability. Colored branches indicate ancient samples. Teal represents ancient black-tailed deer from Vancouver Island, orange from Haida Gwaii, light blue shows ancient deer from Southeast with a mule deer haplotype (old-group), and blue ancient samples from Southeast Alaska with a Sitka black-tailed deer haplotype.

Additional File 6: Figure S5. Admixture cross validation values (--cv=10) from K2 to K6.

Additional File 7: Figure S6. *f3*-statistics plots showing all possible combinations for modern and ancient *Odocoileus hemionus* populations

VI–10

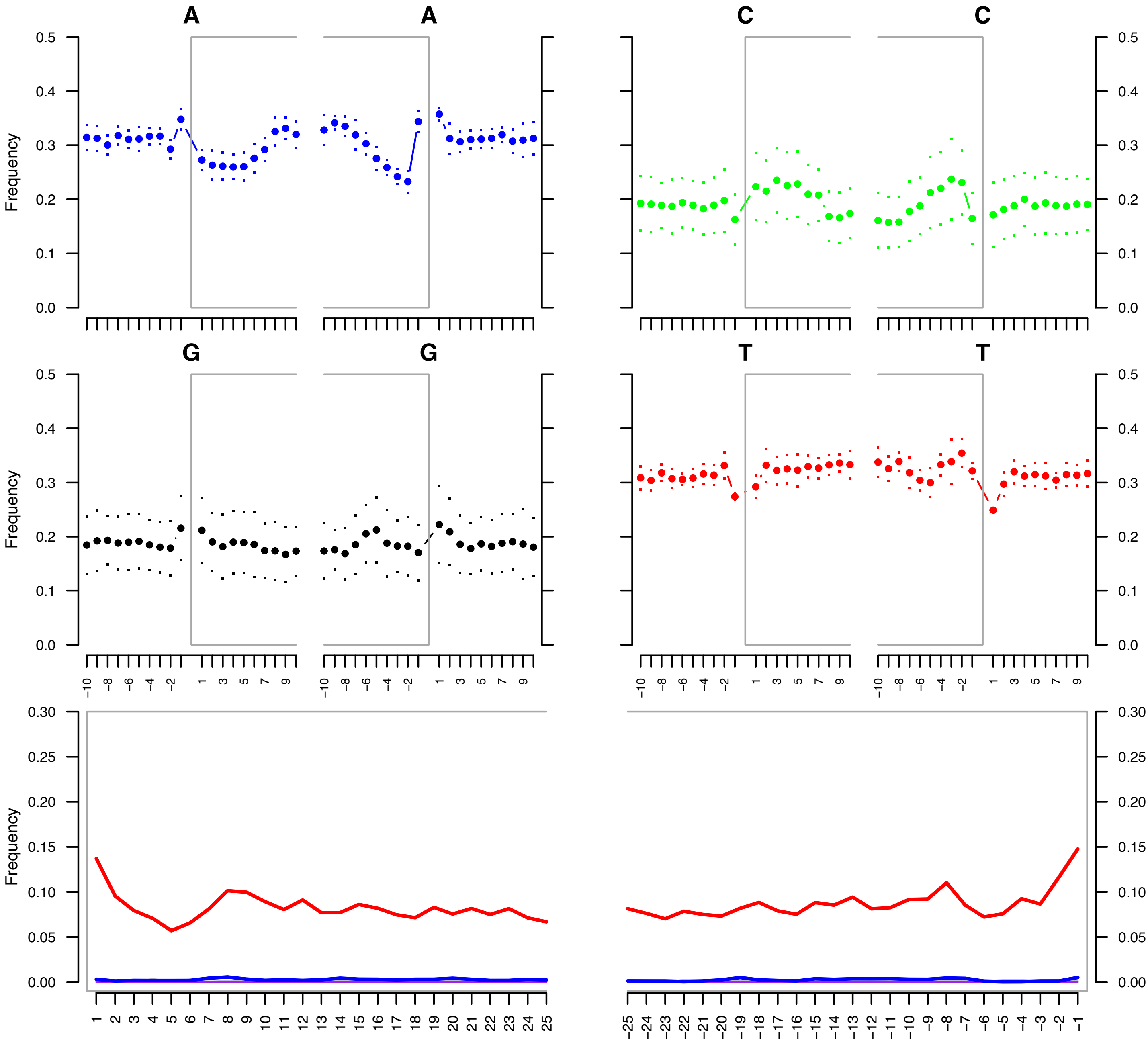

SEAK5

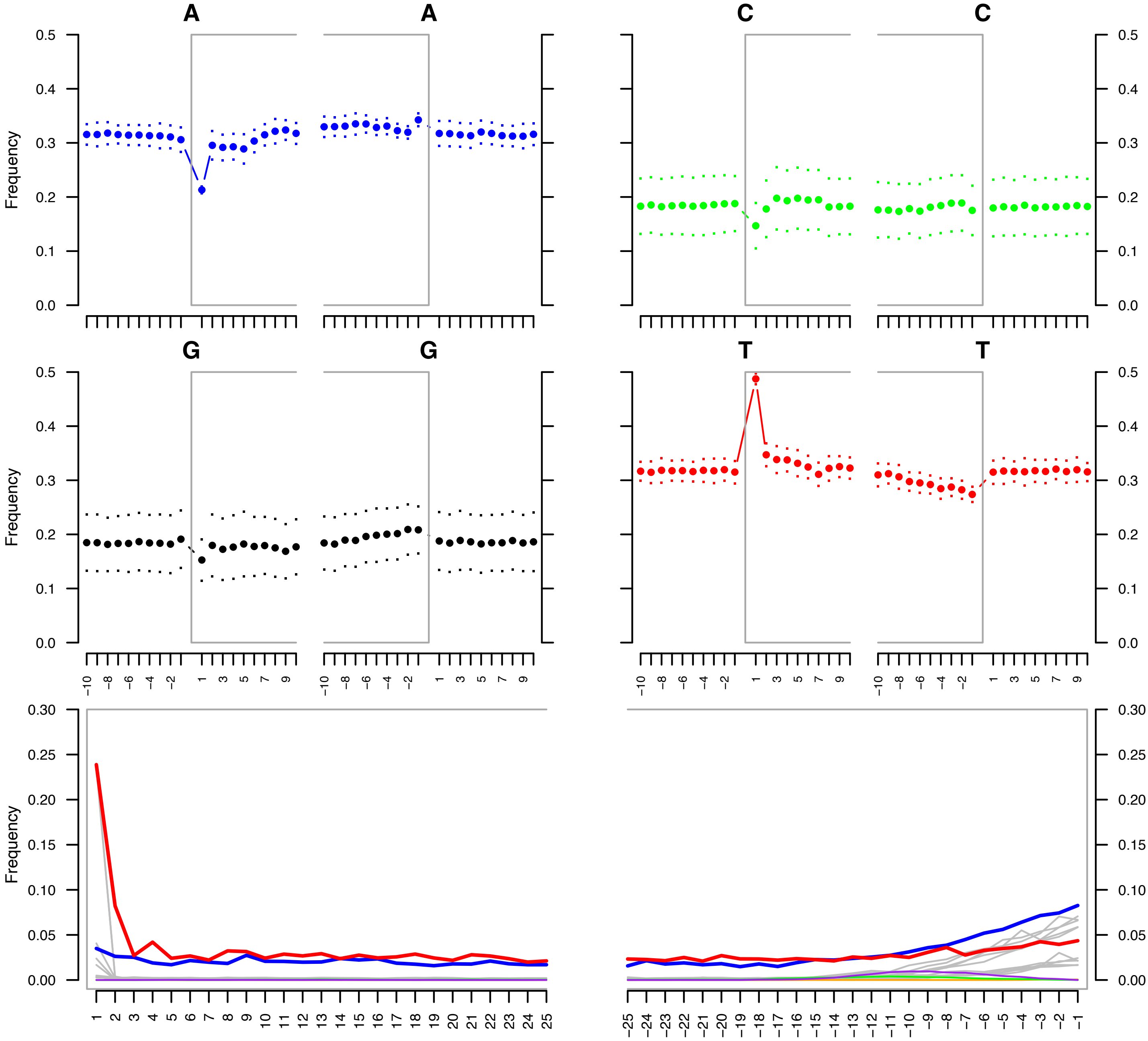

SEAK6

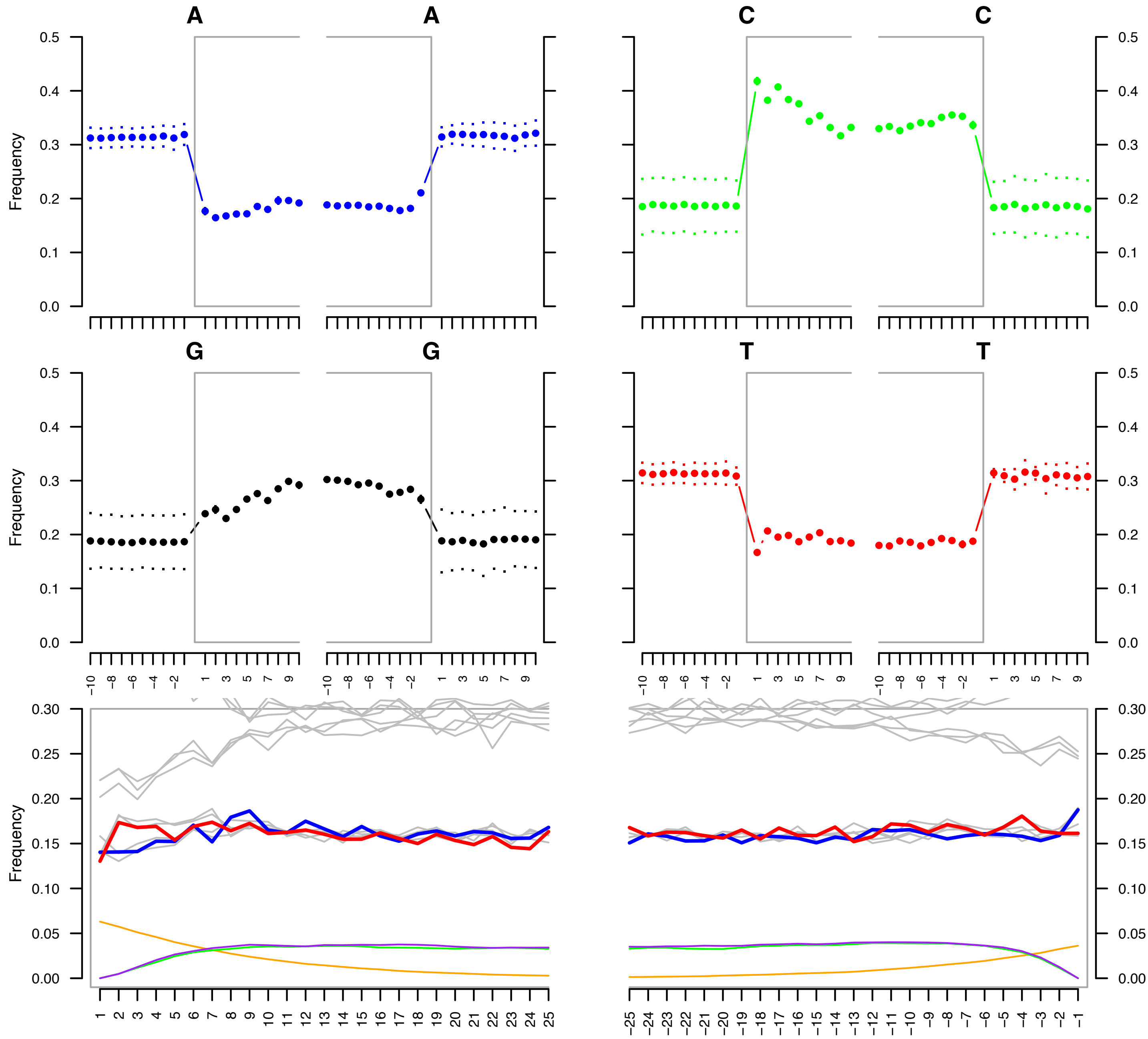

SEAK1

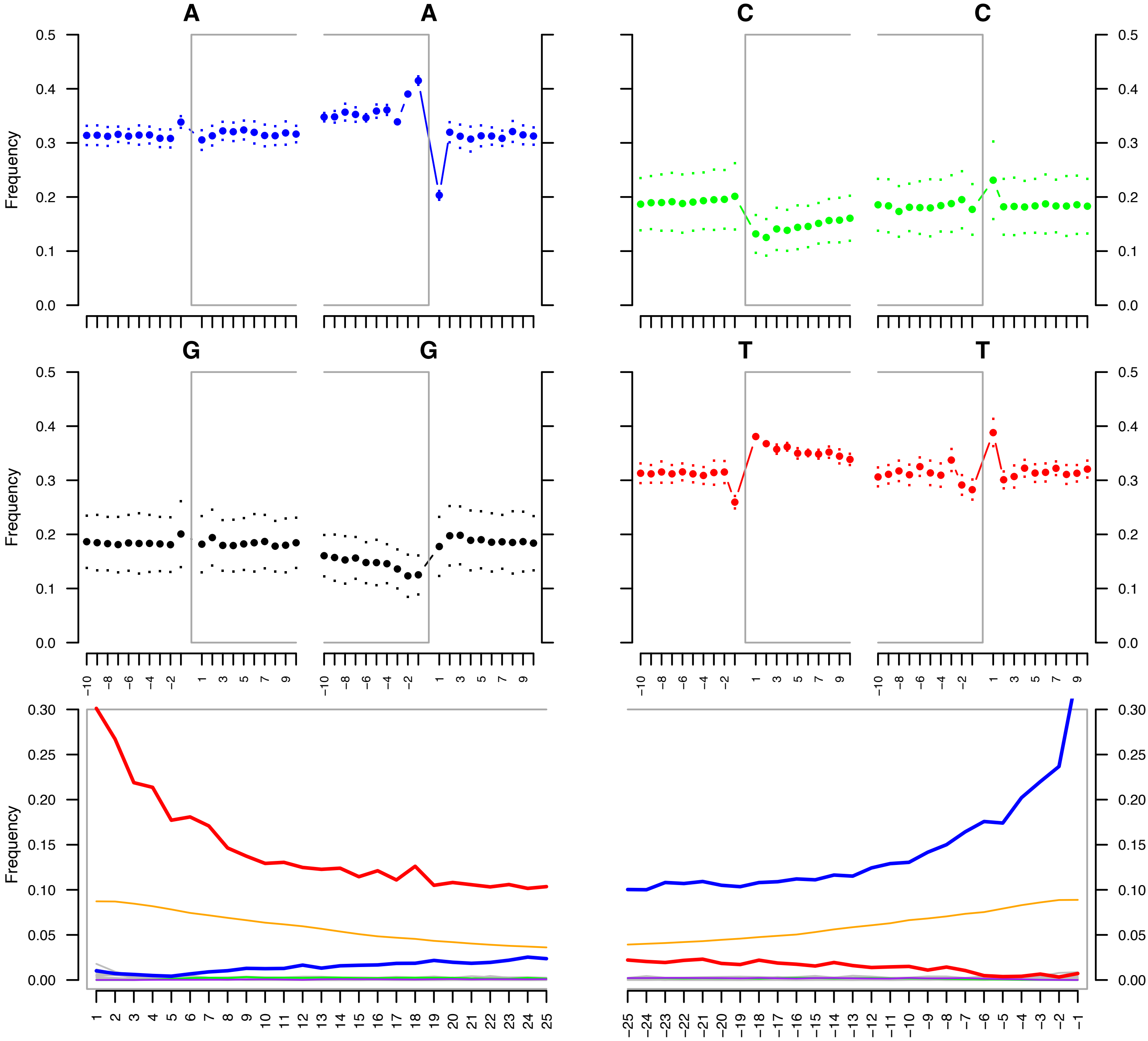

A
A
C
C
G
G
T
T
Frequency
0.0
0.1
0.2
0.3
0.4
0.5
0.00
0.05
0.10
0.15
0.20
0.25
0.30

SEAK4

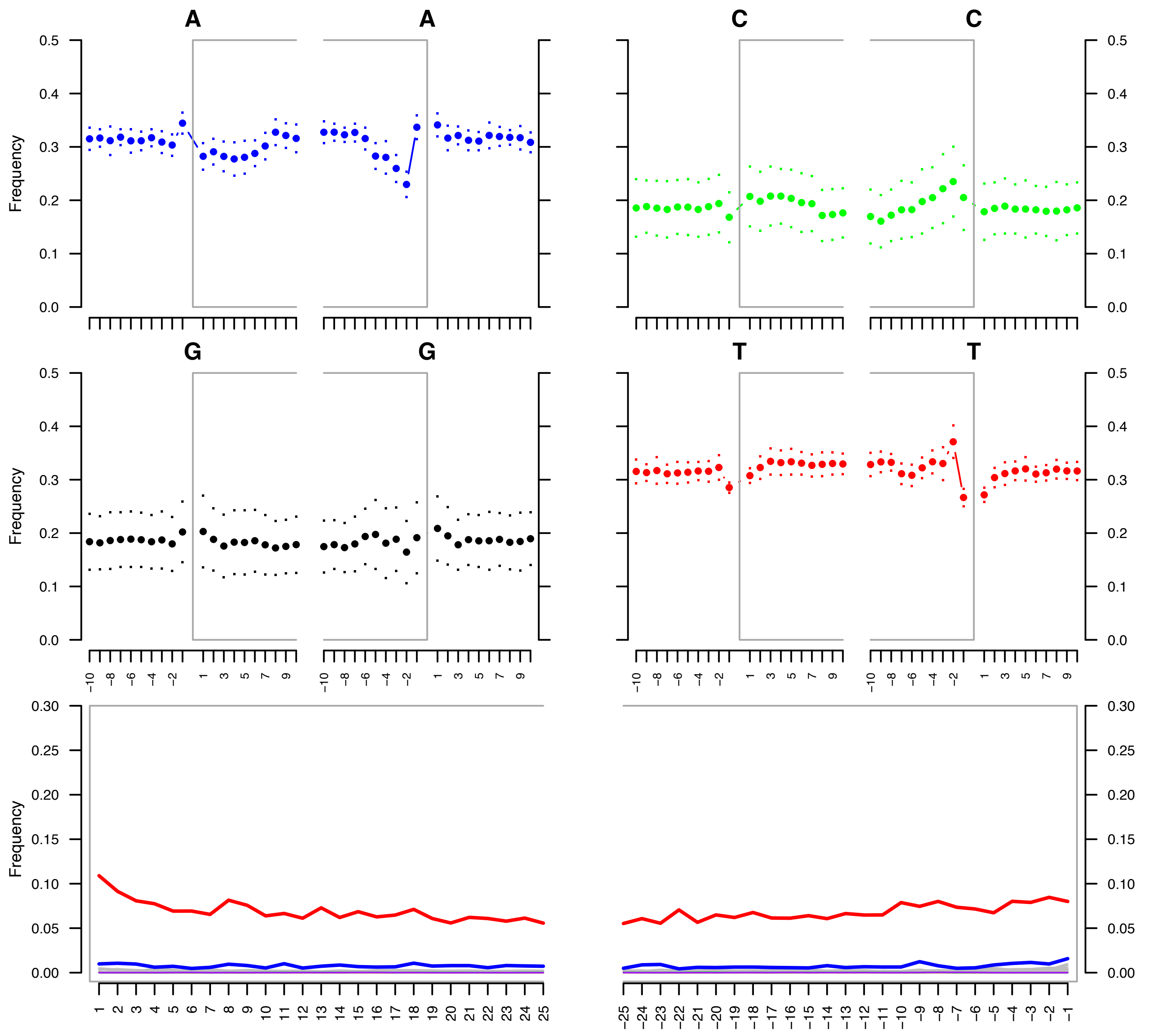

A
A
C
C
G
G
T
T
Frequency
Frequency
Frequency
0.0
0.1
0.2
0.3
0.4
0.5
0.00
0.05
0.10
0.15
0.20
0.25
0.30

VI–9

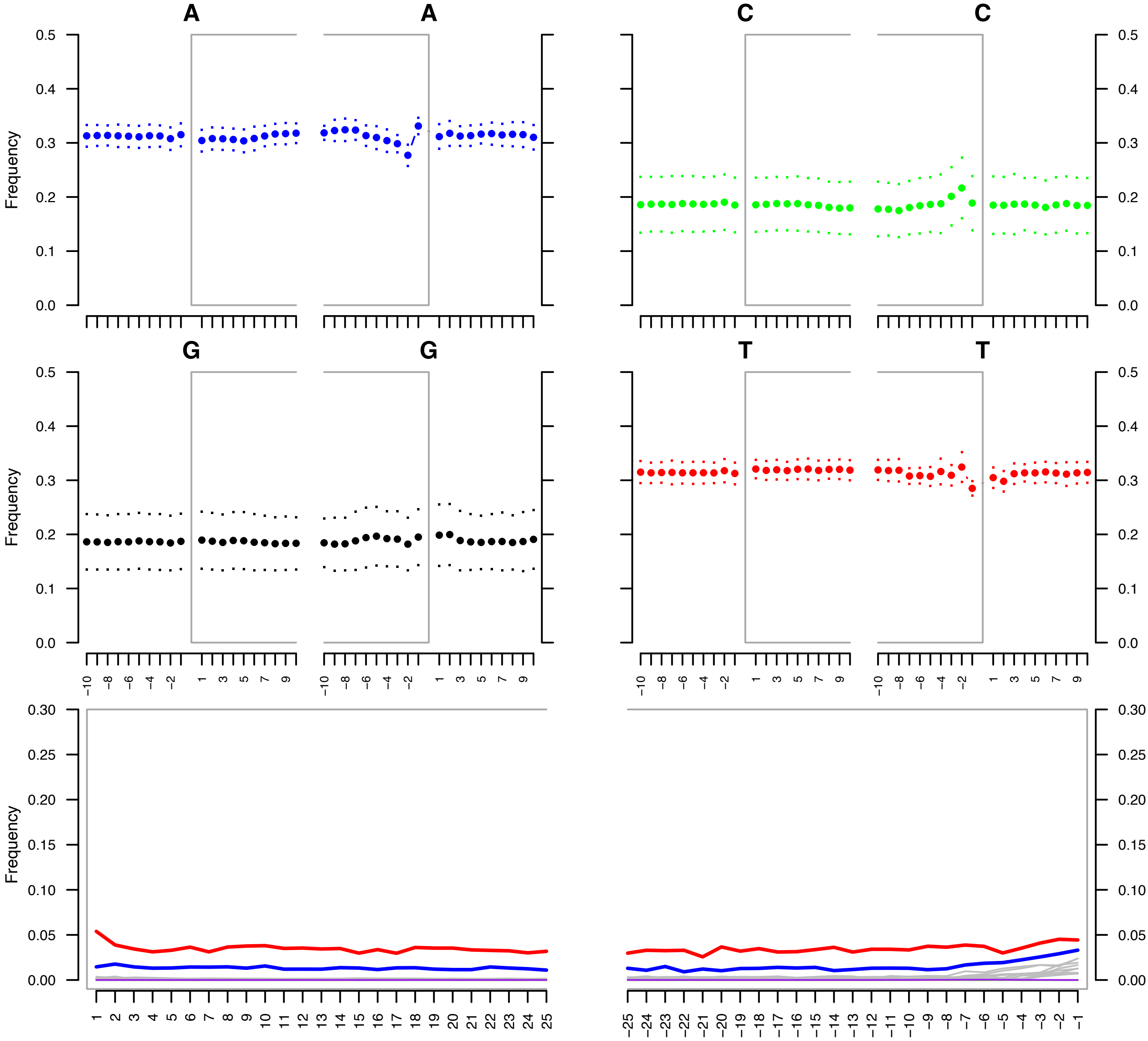
A
A
C
C
G
G
T
T
Frequency
Frequency
Frequency
0.0
0.1
0.2
0.3
0.4
0.5
0.00
0.05
0.10
0.15
0.20
0.25
0.30

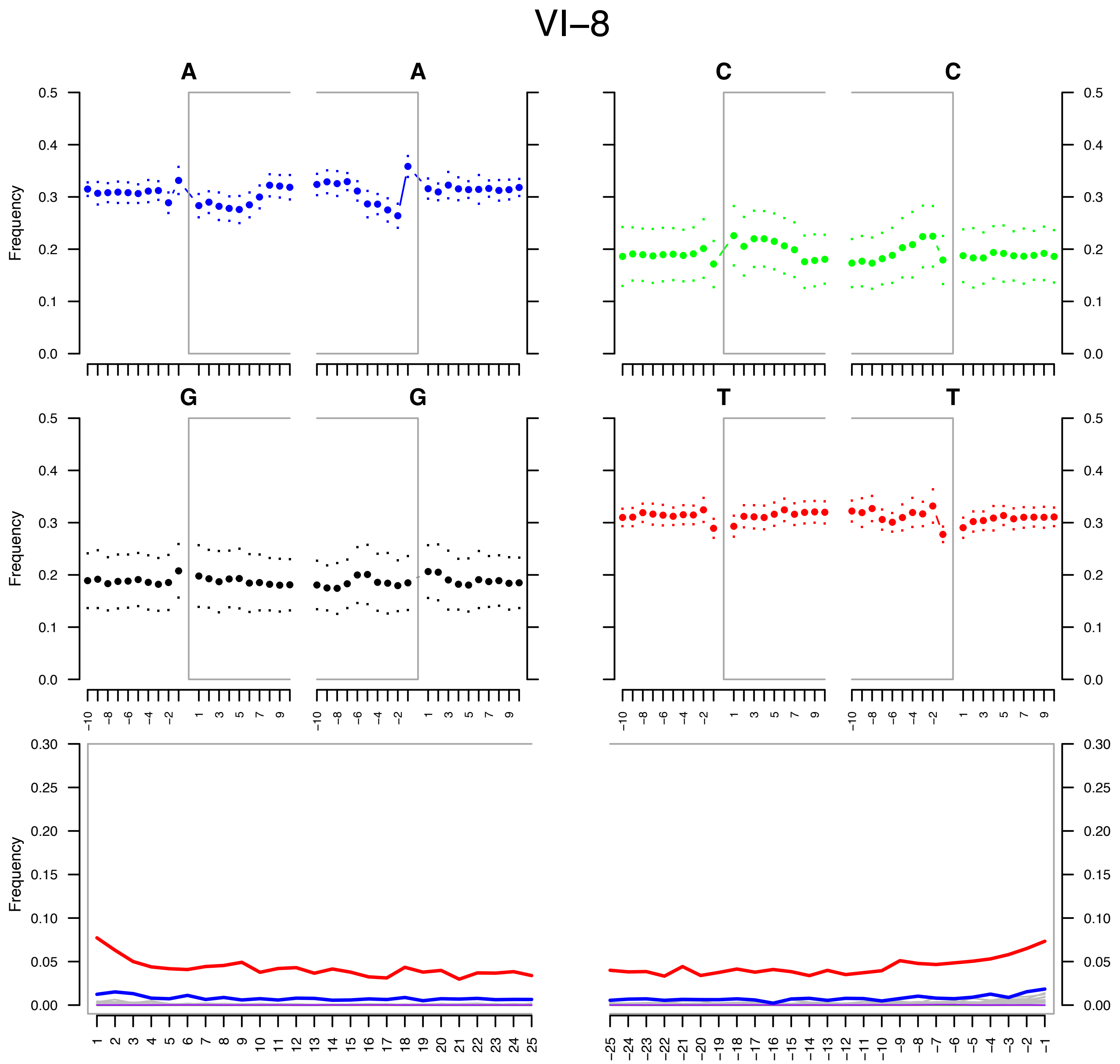
VI–8
A
A
C
C
G
G
T
T
Frequency
0.0
0.1
0.2
0.3
0.4
0.5
0.00
0.05
0.10
0.15
0.20
0.25
0.30
−10 −8 −6 −4 −2 1 3 5 7 9
1 2 3 4 5 6 7 8 9 10 11 12 13 14 15 16 17 18 19 20 21 22 23 24 25
−25 −24 −23 −22 −21 −20 −19 −18 −17 −16 −15 −14 −13 −12 −11 −10 −9 −8 −7 −6 −5 −4 −3 −2 −1

VI–7

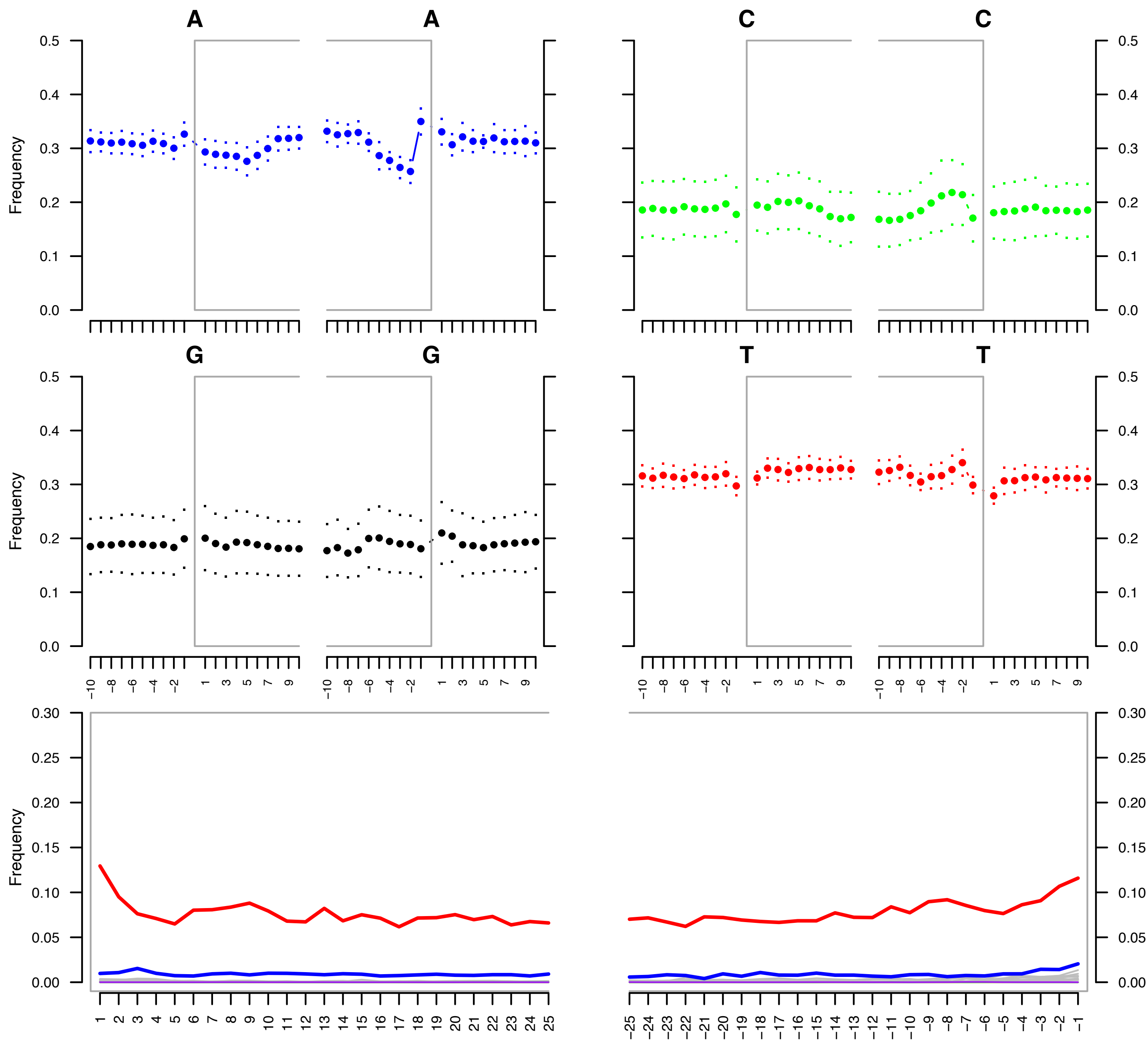
A
A
C
C
G
G
T
T
Frequency
0.0
0.1
0.2
0.3
0.4
0.5
0.00
0.05
0.10
0.15
0.20
0.25
0.30

VI–6

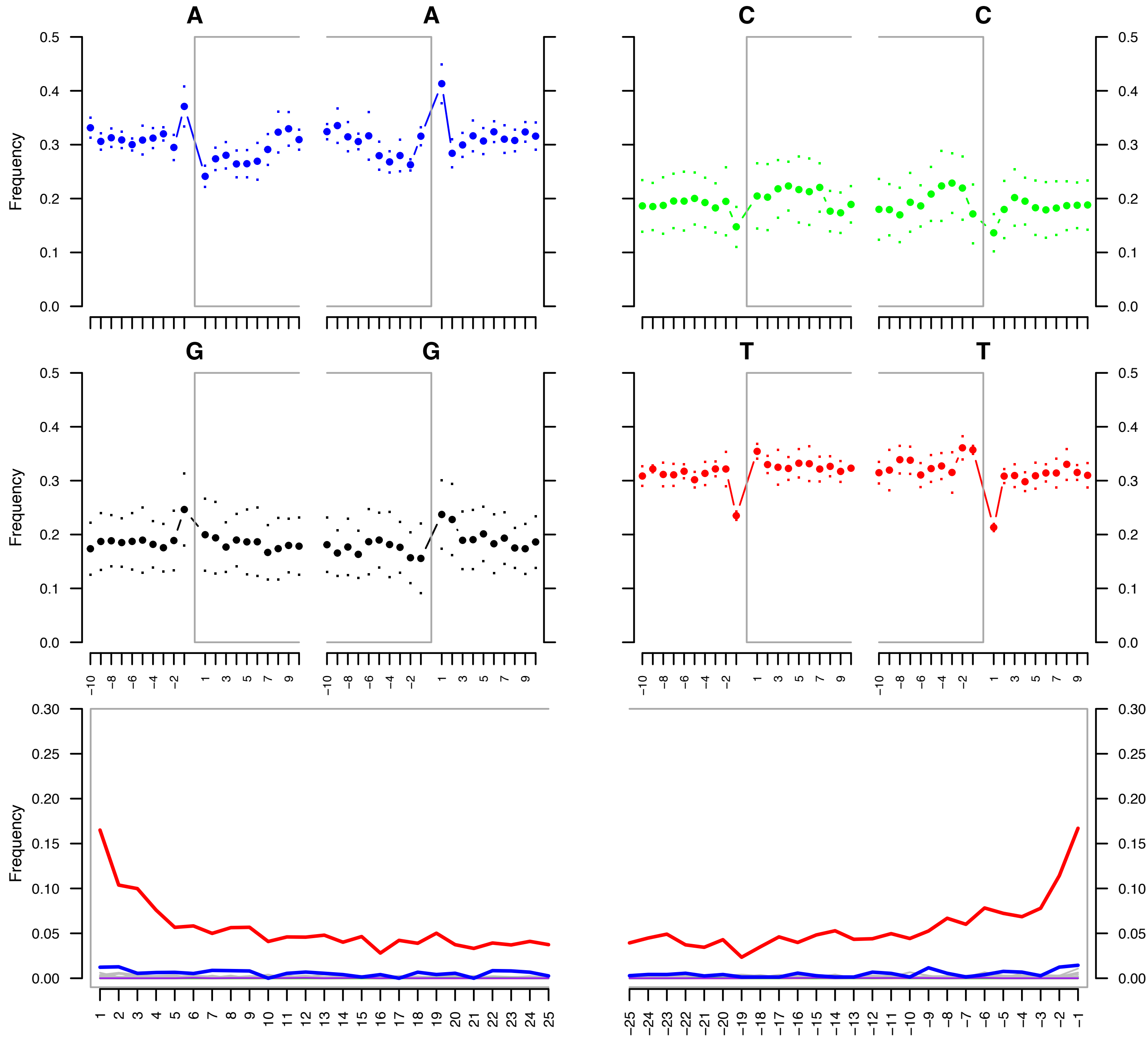

VI–5

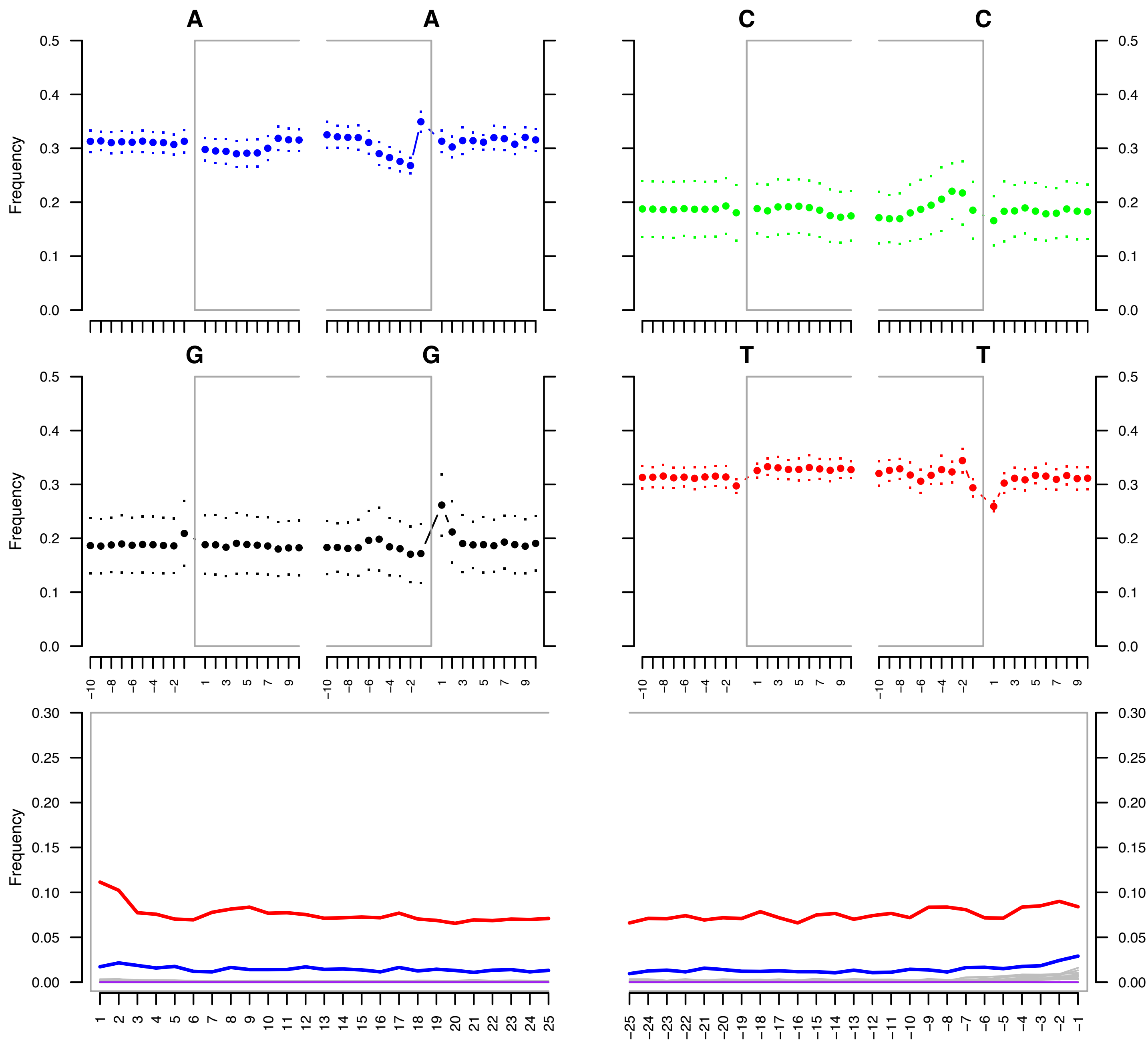

VI–4

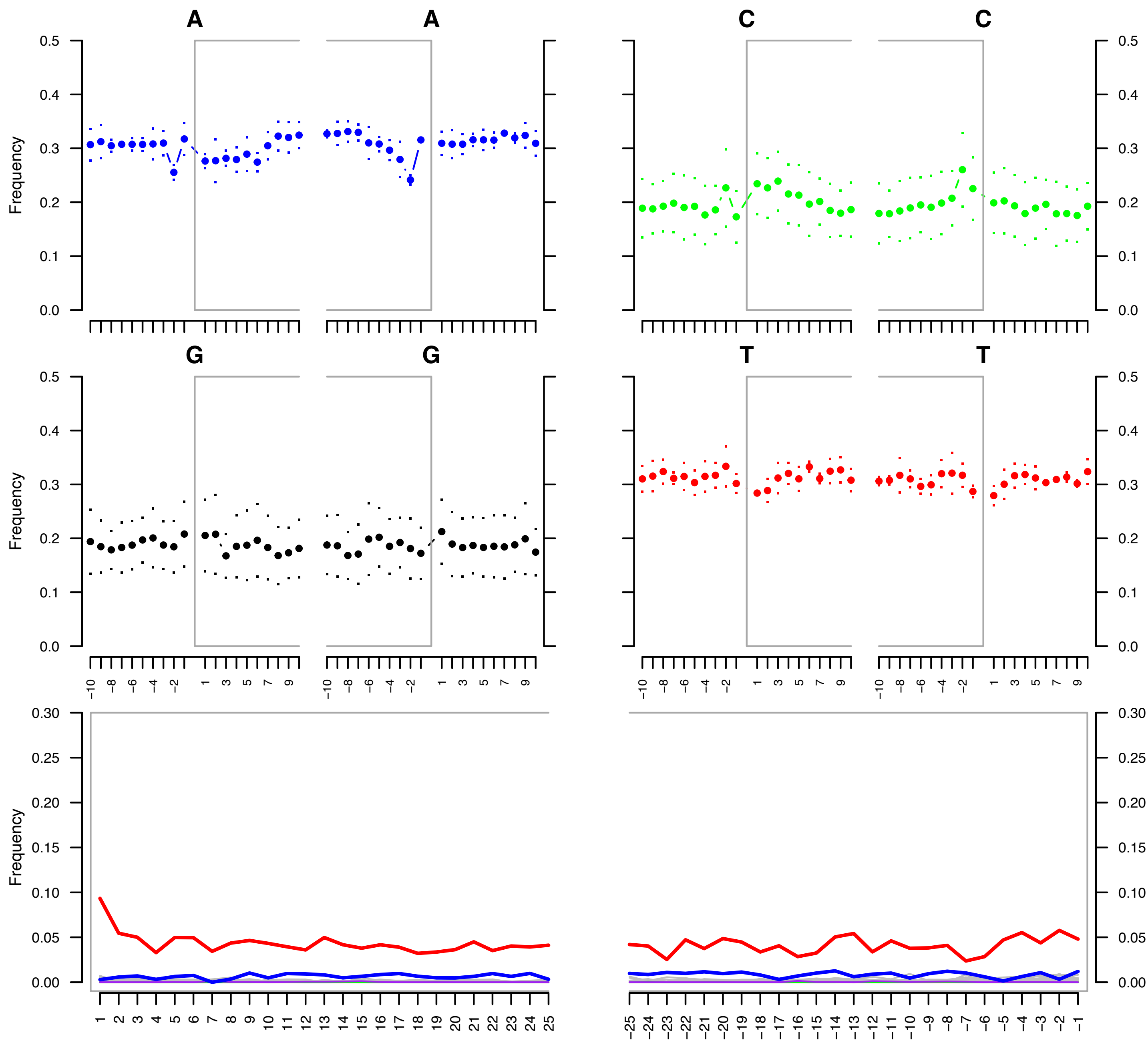

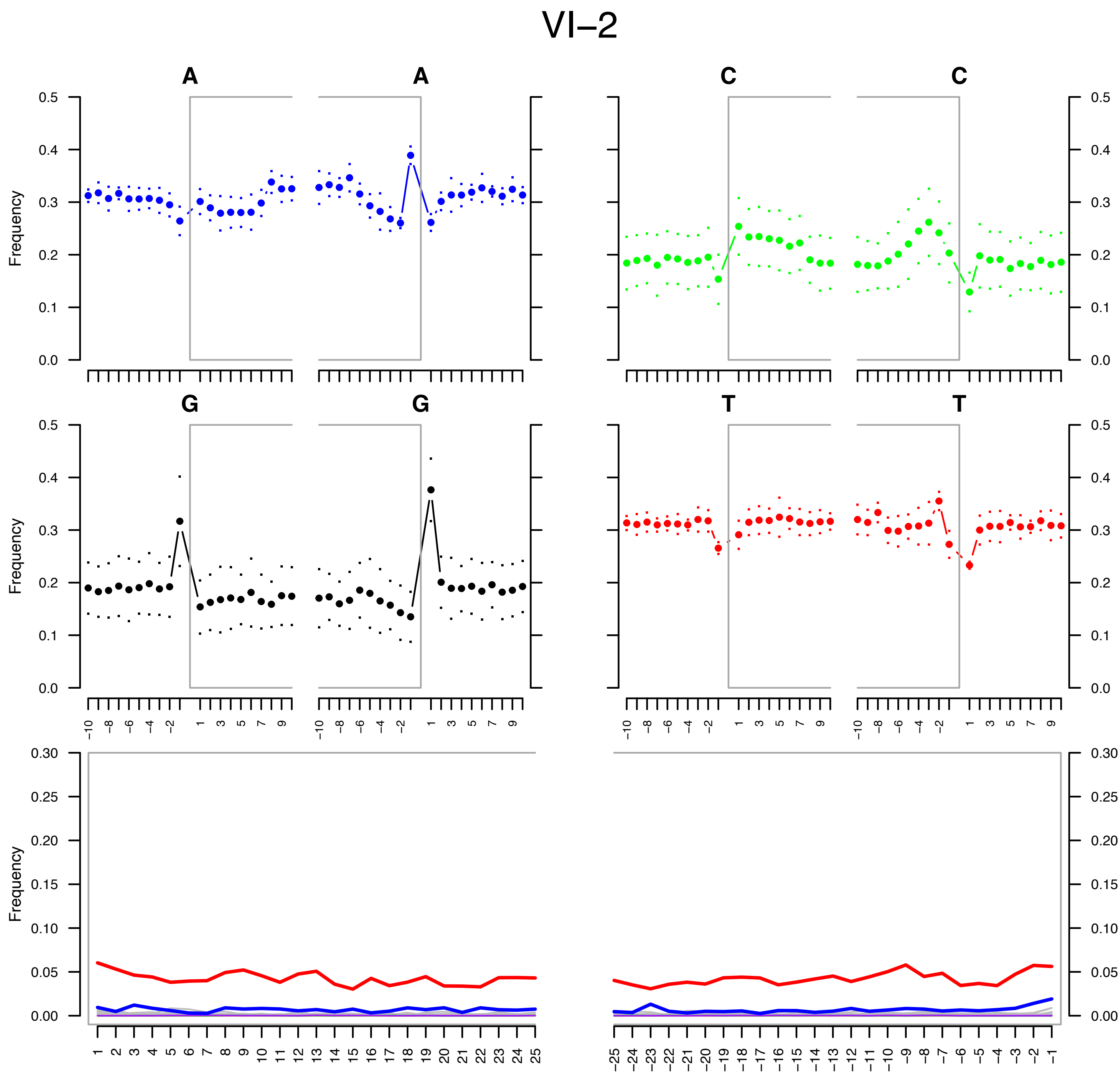
VI–2
A
A
C
C
G
G
T
T
Frequency
Frequency
Frequency
0.0
0.1
0.2
0.3
0.4
0.5
0.00
0.05
0.10
0.15
0.20
0.25
0.30

VI–1

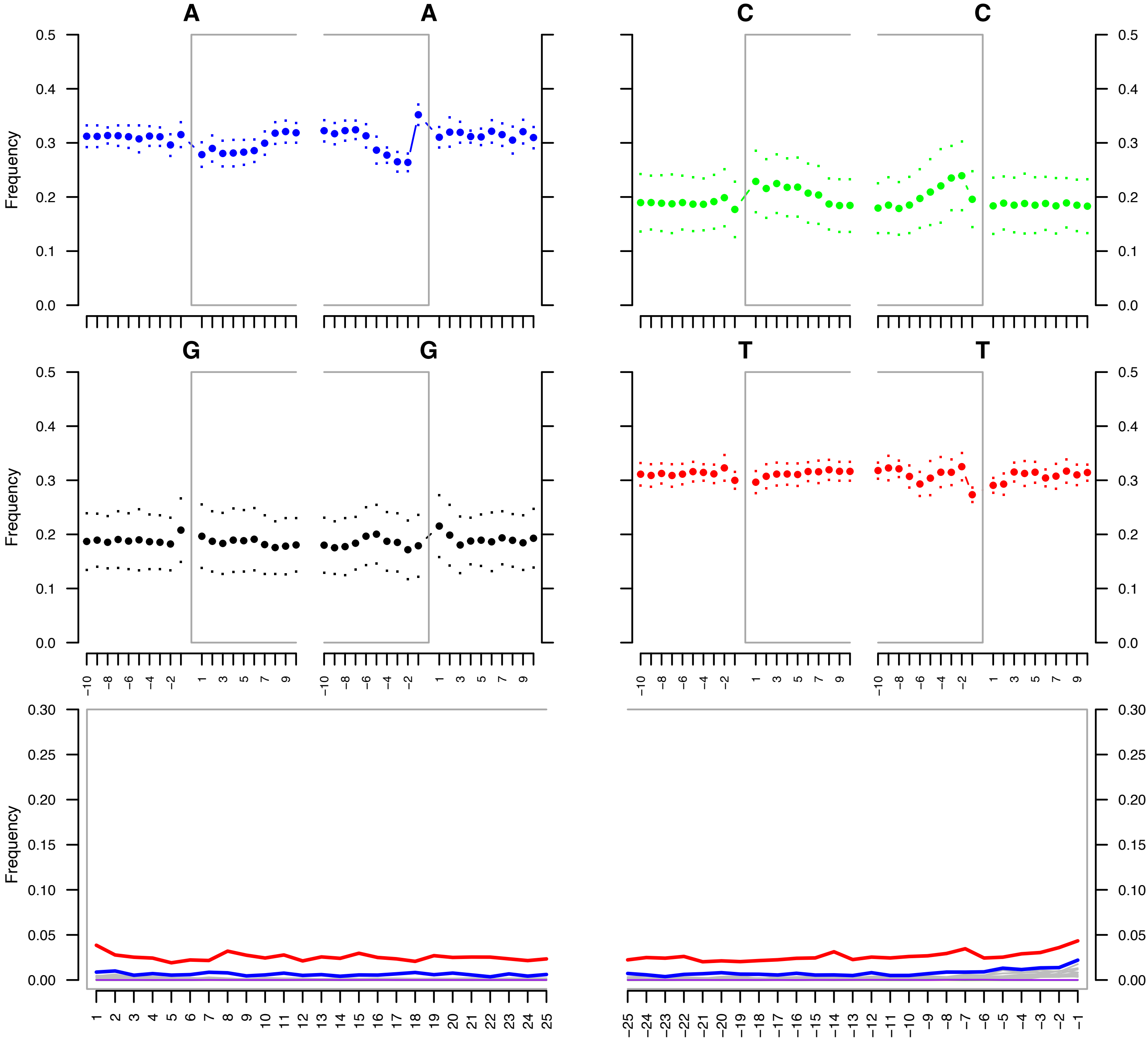

SEAK7

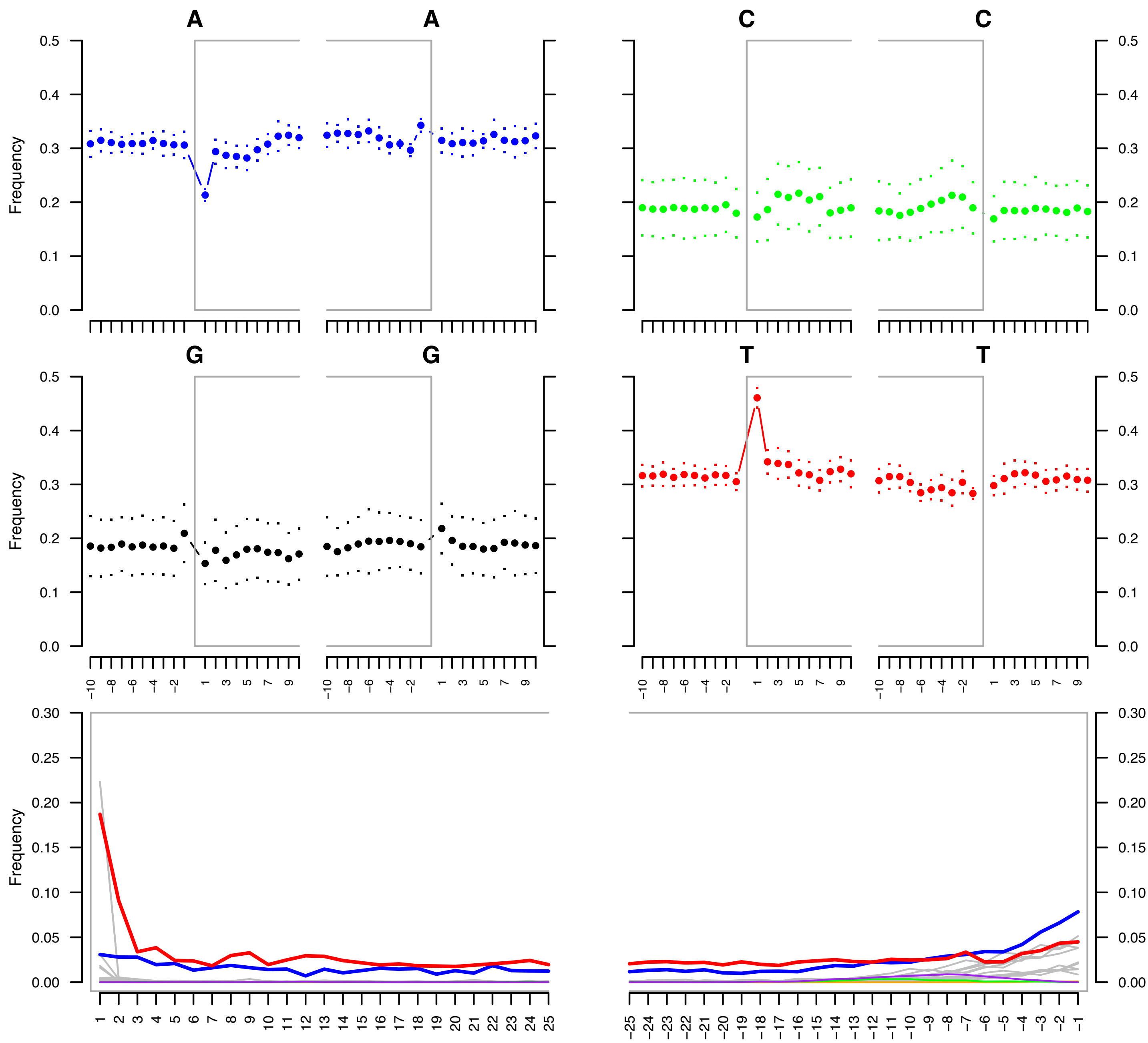

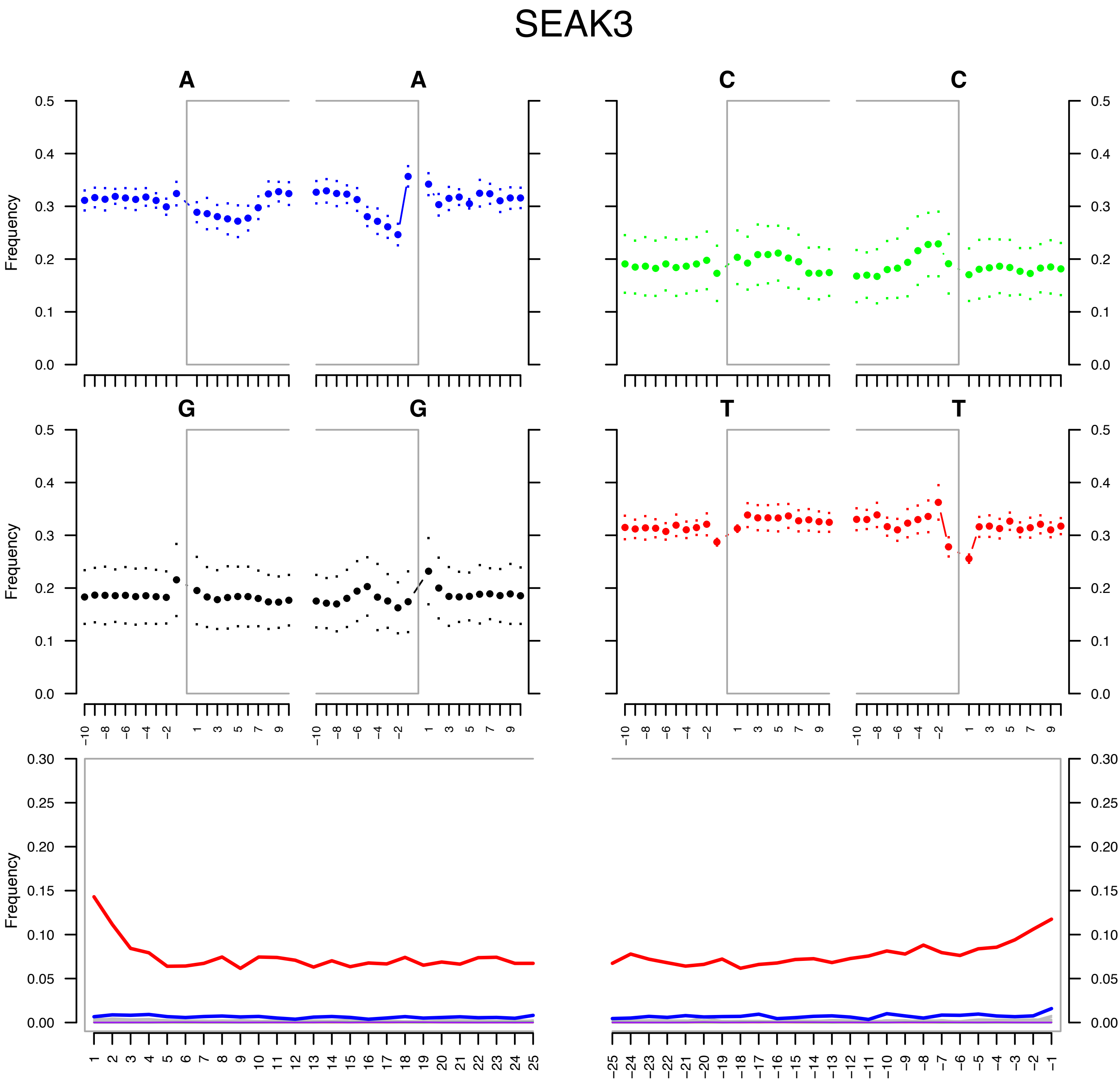
SEAK3
A
A
C
C
G
G
T
T
Frequency
Frequency
Frequency

VI-11

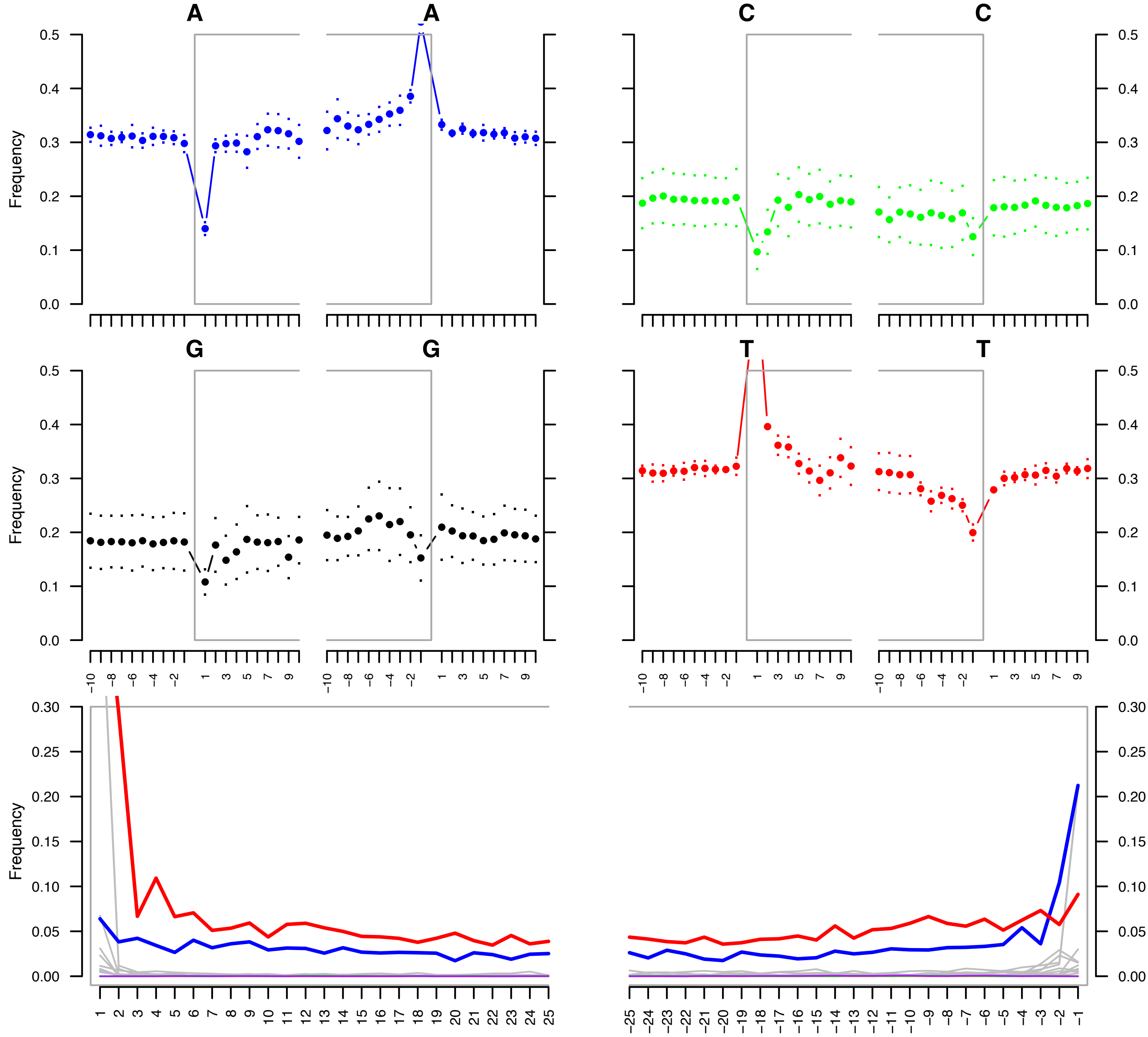

HG3

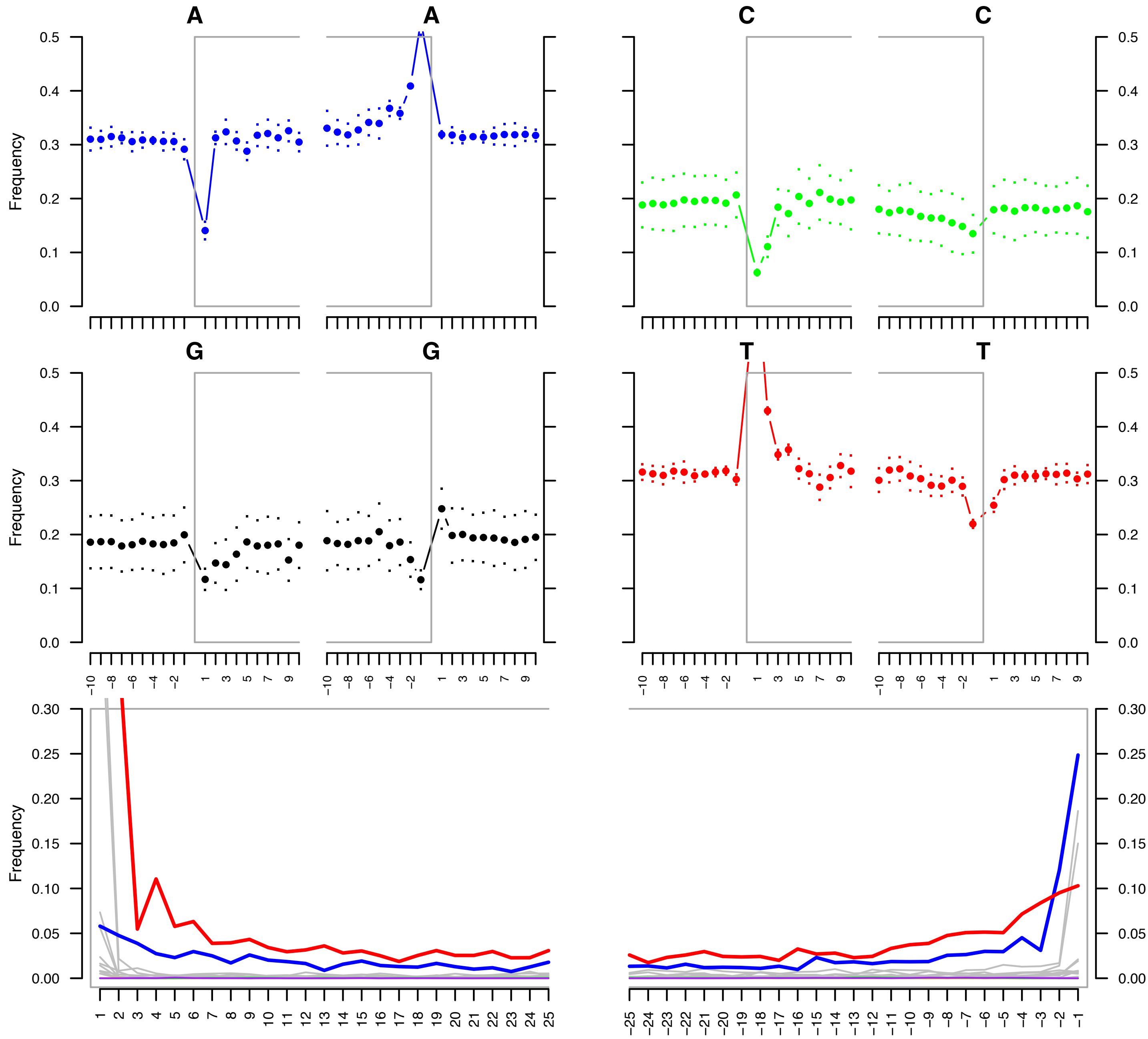

HG2

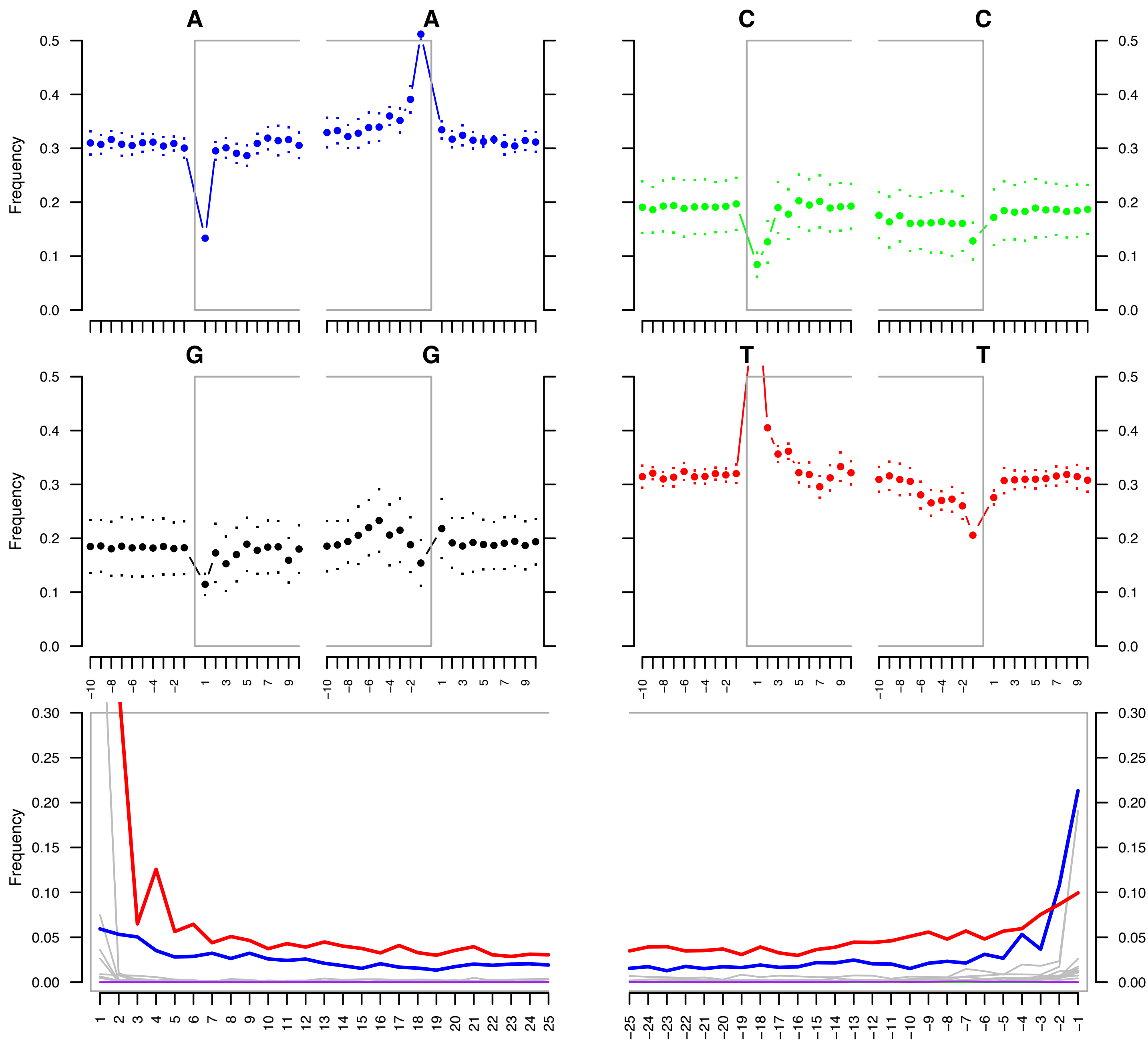

HG1

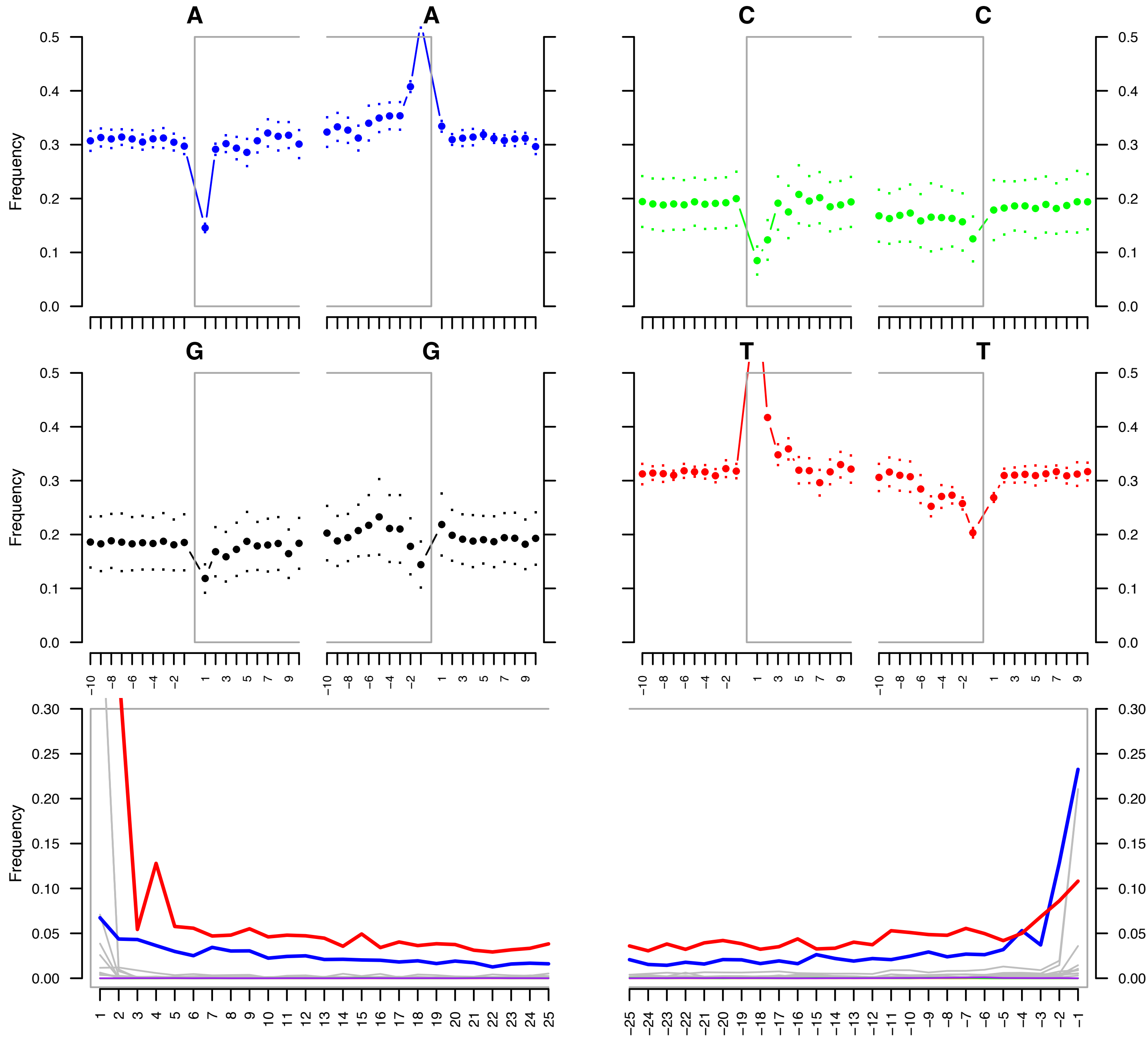

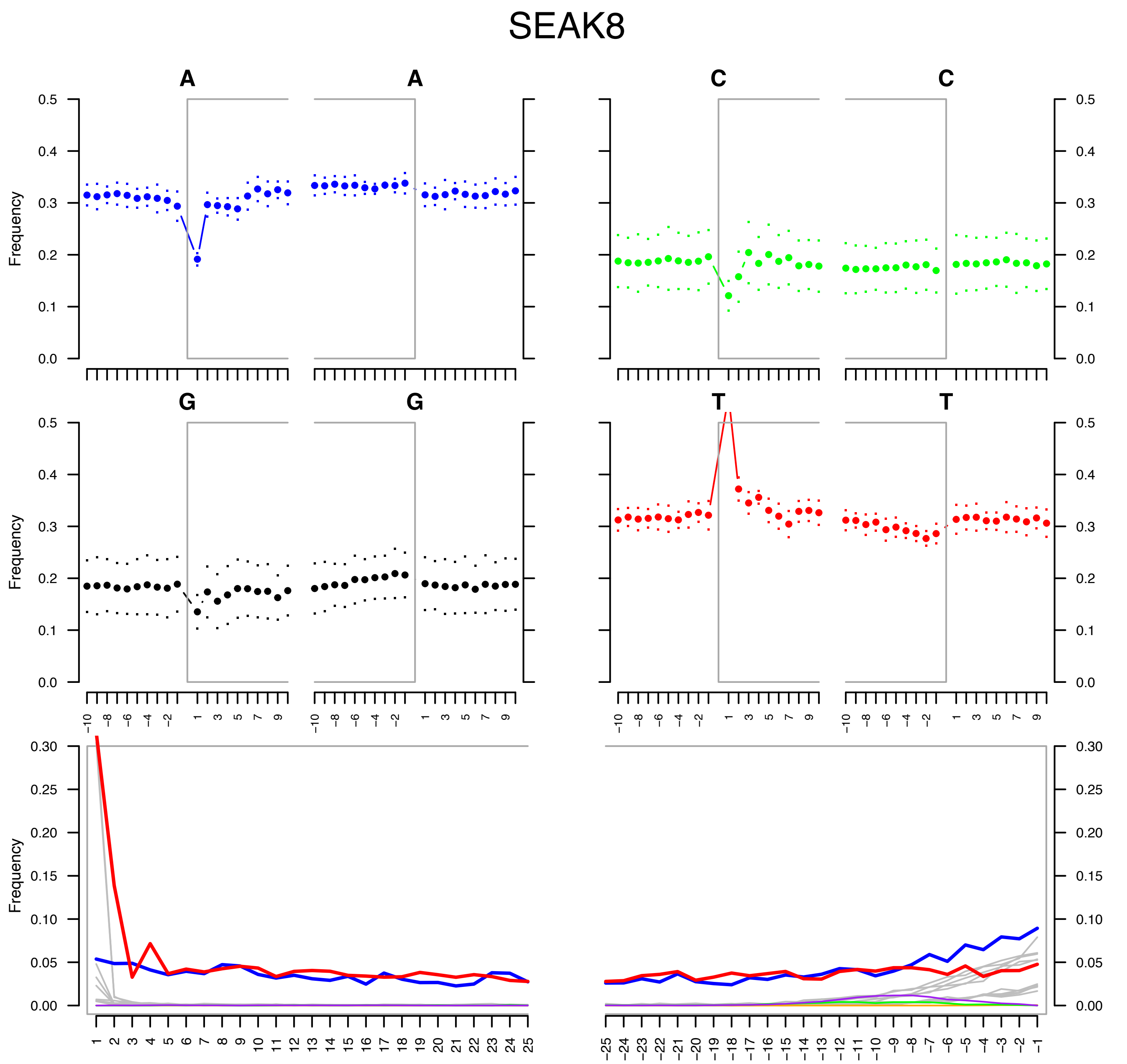
SEAK8
A
A
C
C
G
G
T
T
Frequency
Frequency
Frequency

SEAK2

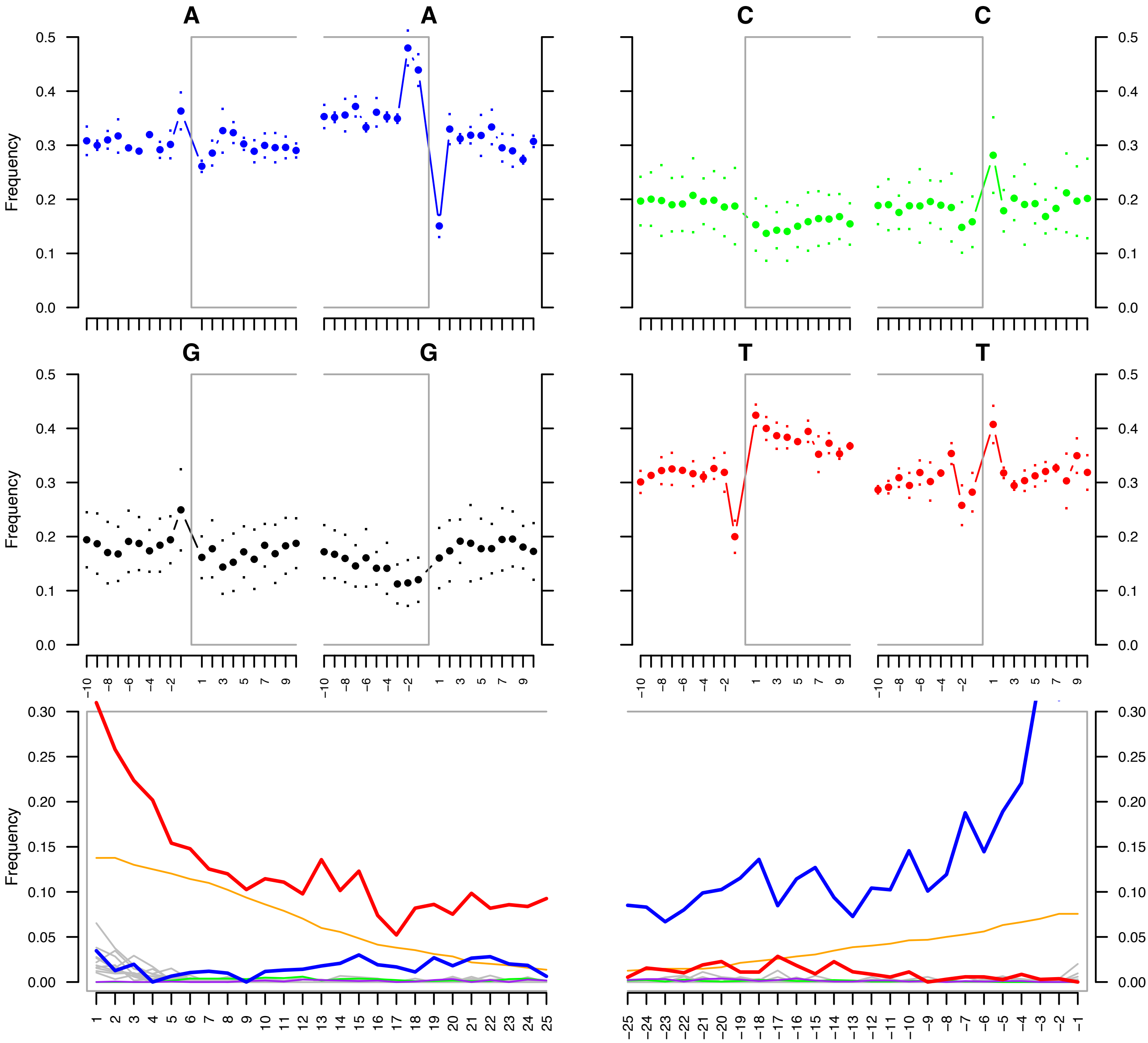

SEAK3

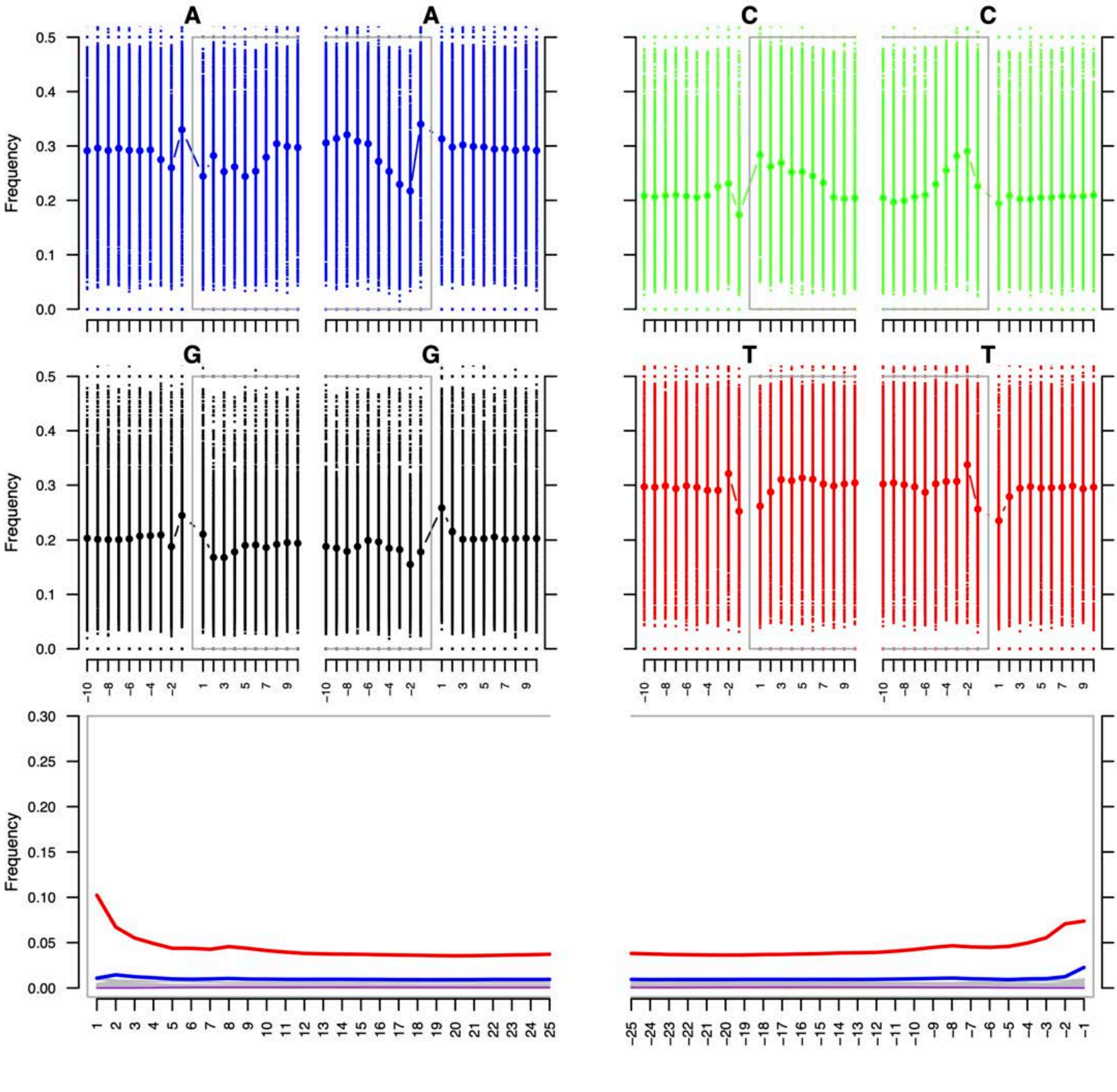
A
A
C
C
G
G
T
T
Frequency
Frequency
Frequency
0.0
0.1
0.2
0.3
0.4
0.5
0.00
0.05
0.10
0.15
0.20
0.25
0.30
−10
−8
−6
−4
−2
1
3
5
7
9
1
2
3
4
5
6
7
8
9
10
11
12
13
14
15
16
17
18
19
20
21
22
23
24
25
−25
−24
−23
−22
−21
−20
−19
−18
−17
−16
−15
−14
−13
−12
−11
−10
−9
−8
−7
−6
−5
−4
−3
−2
−1

SEAK4

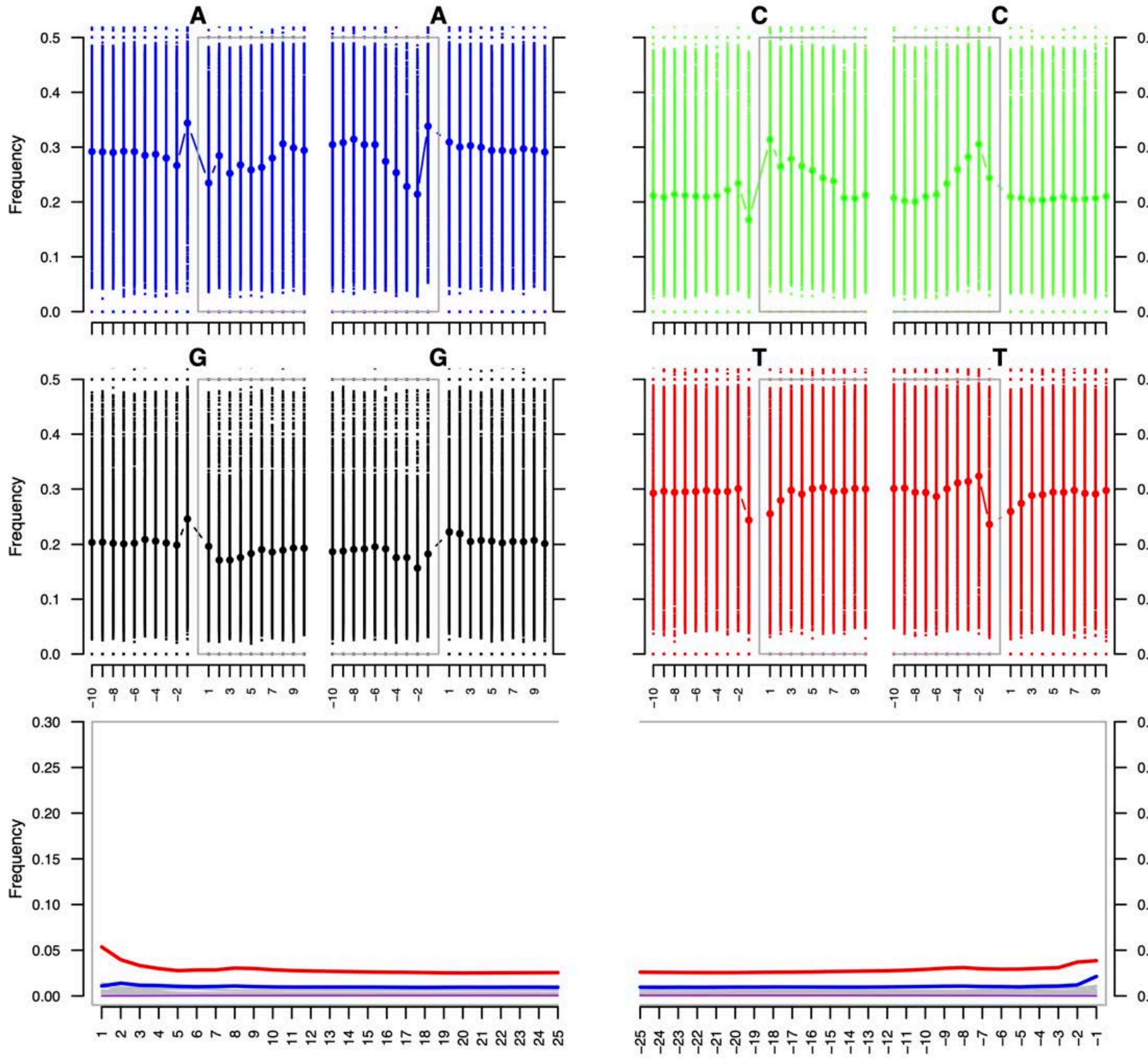

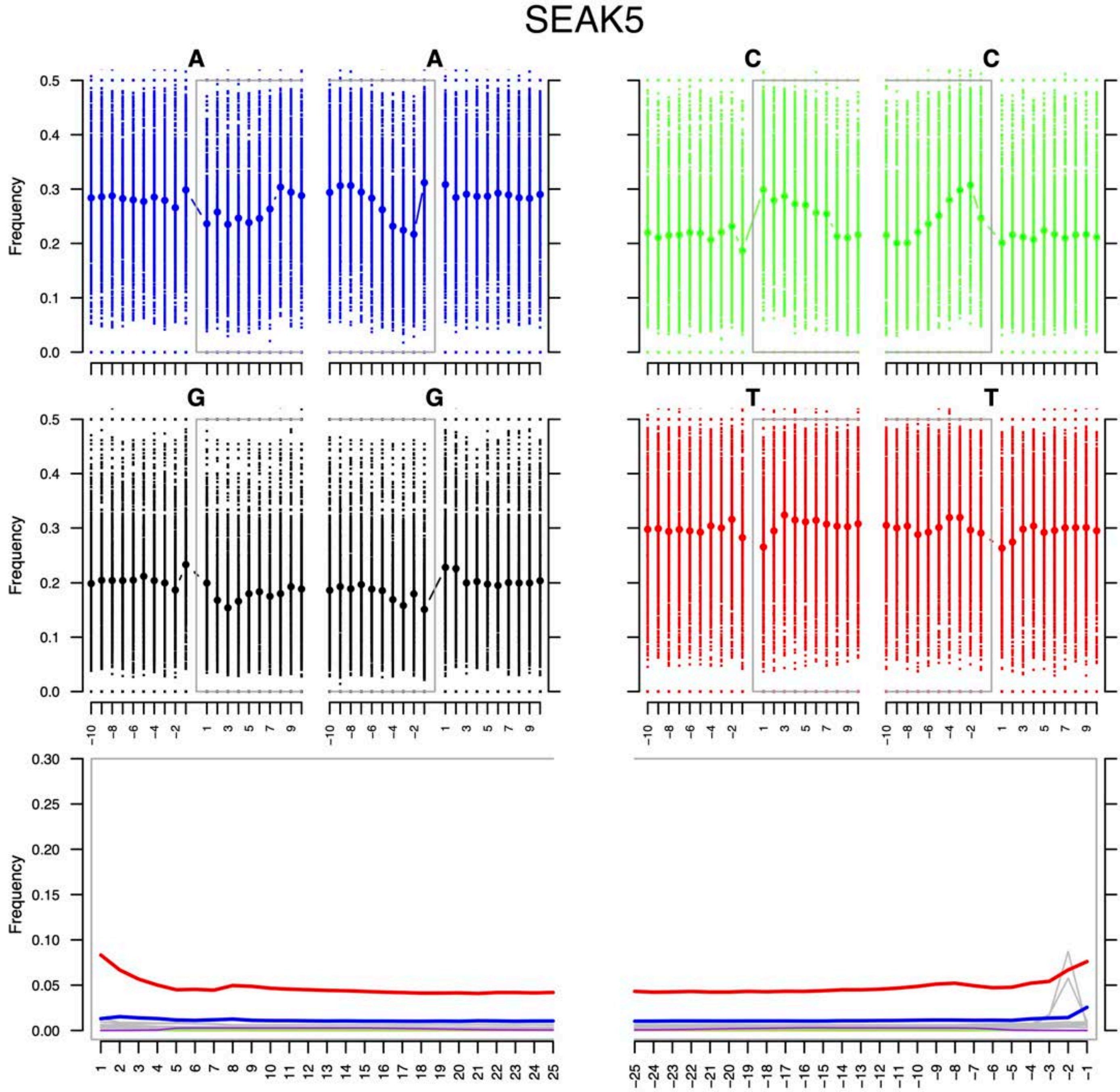
SEAK5
A
A
C
C
G
G
T
T
Frequency
Frequency
Frequency

SEAK6

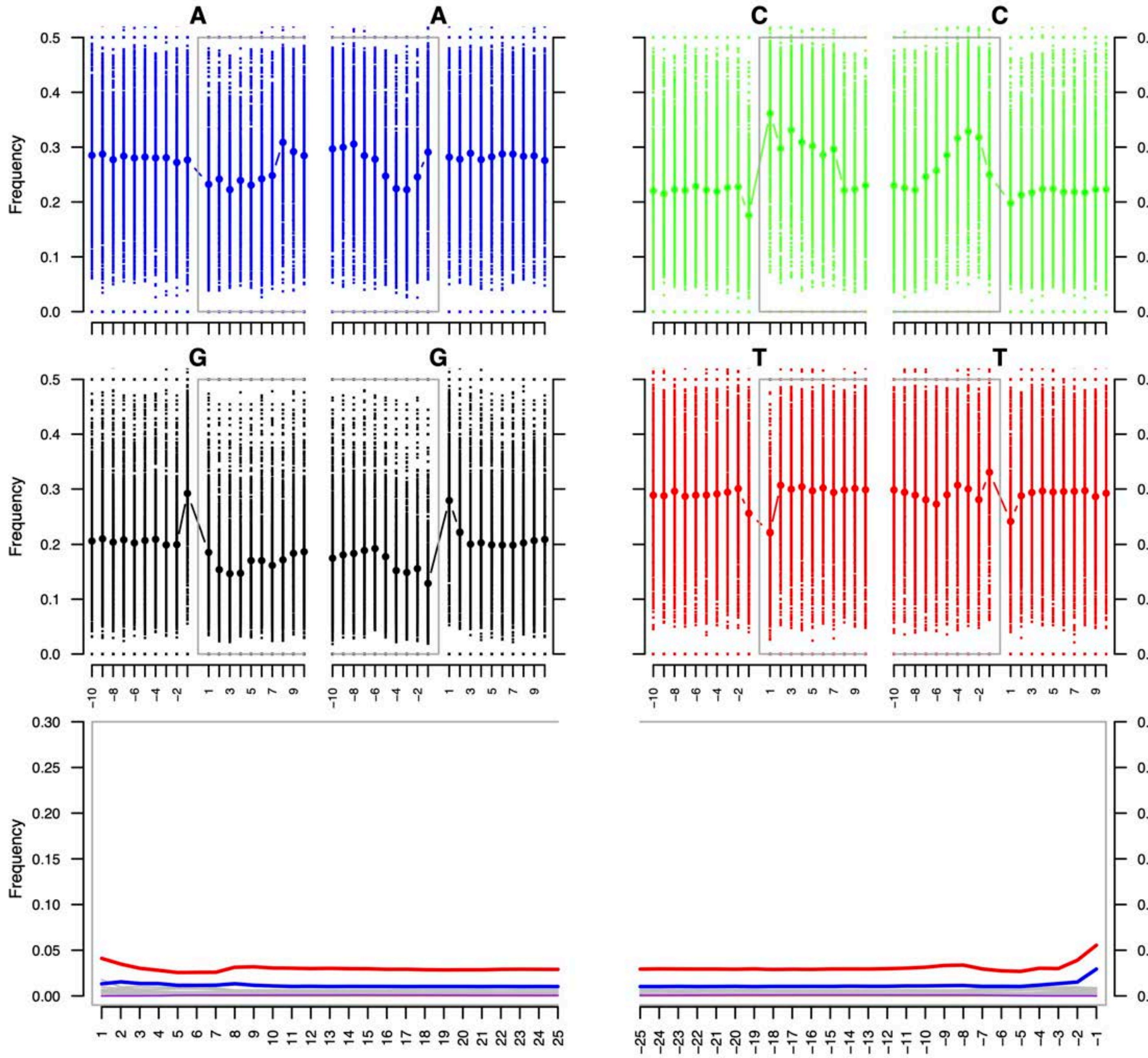

SEAK7

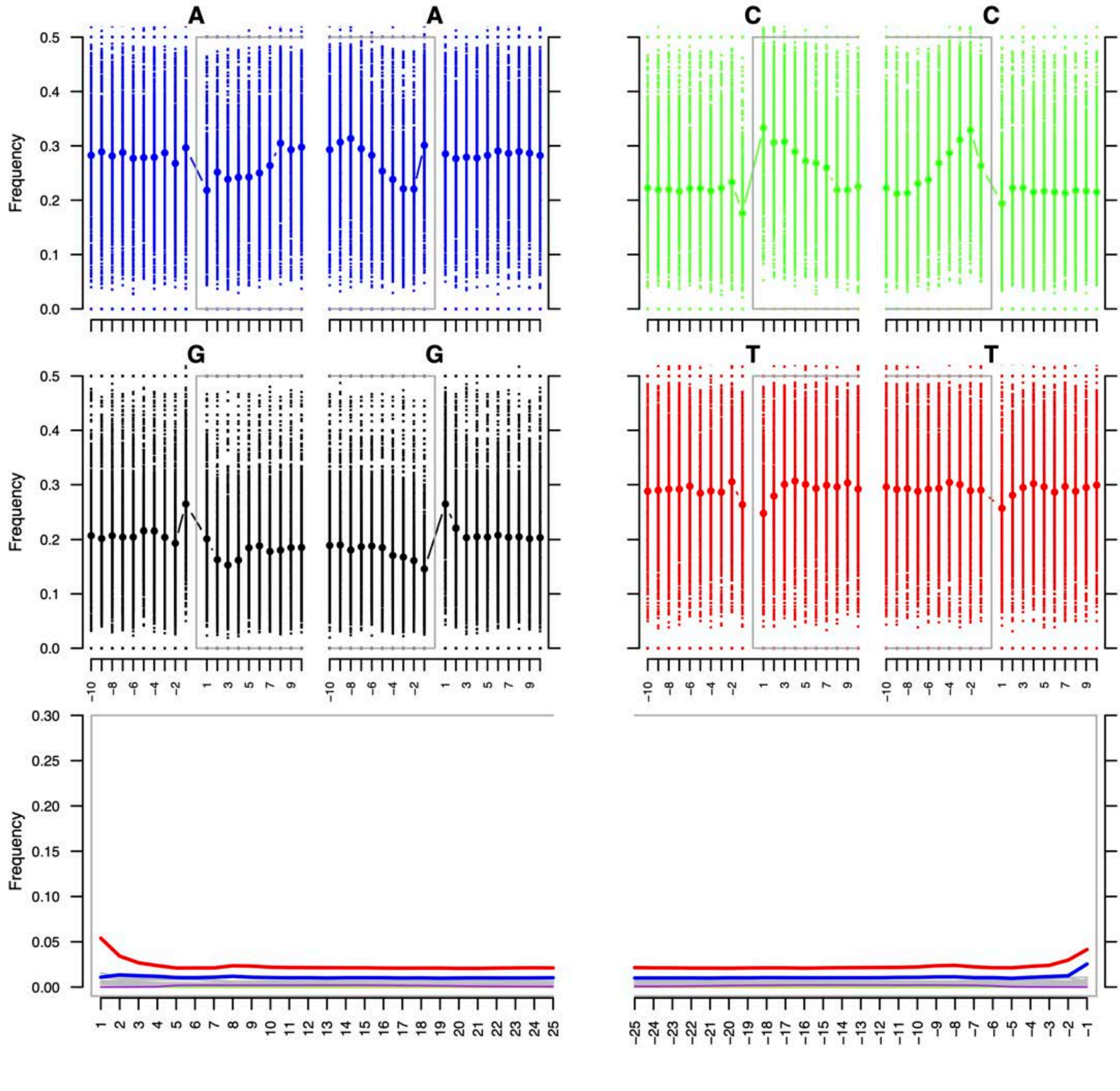

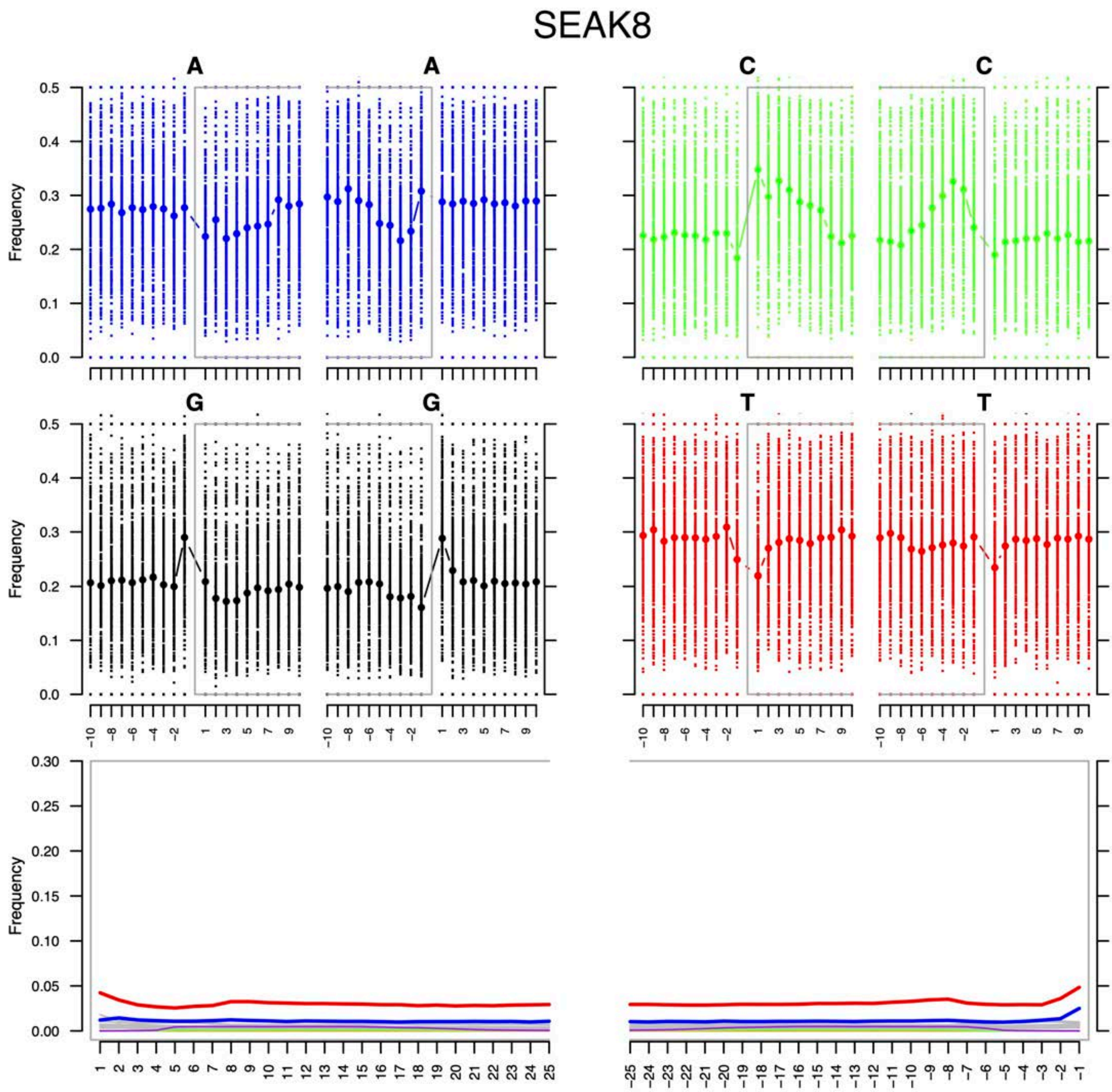
SEAK8
A
A
C
C
G
G
T
T
Frequency

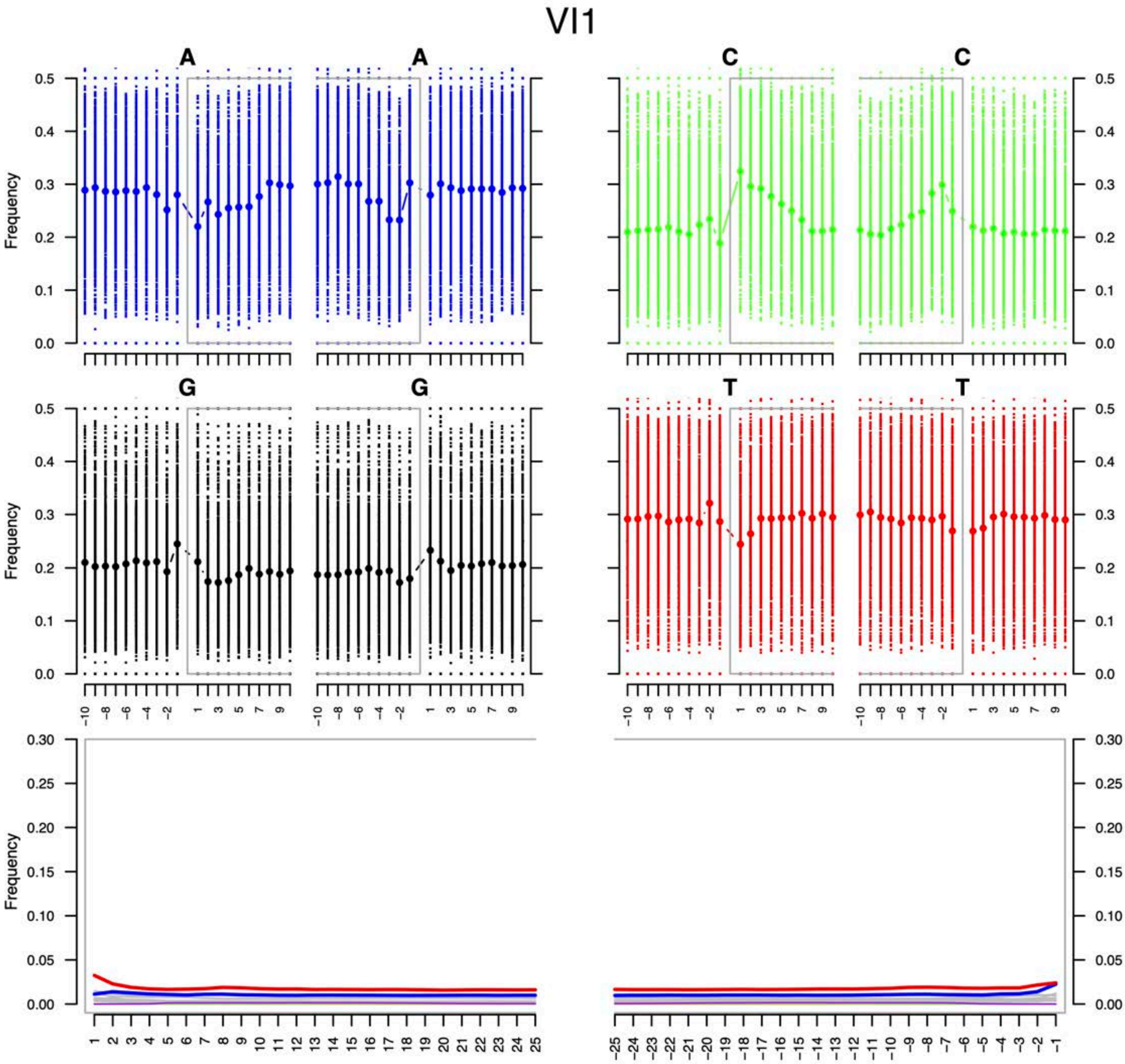
VI1
A
A
C
C
G
G
T
T
Frequency
Frequency
Frequency

VI2

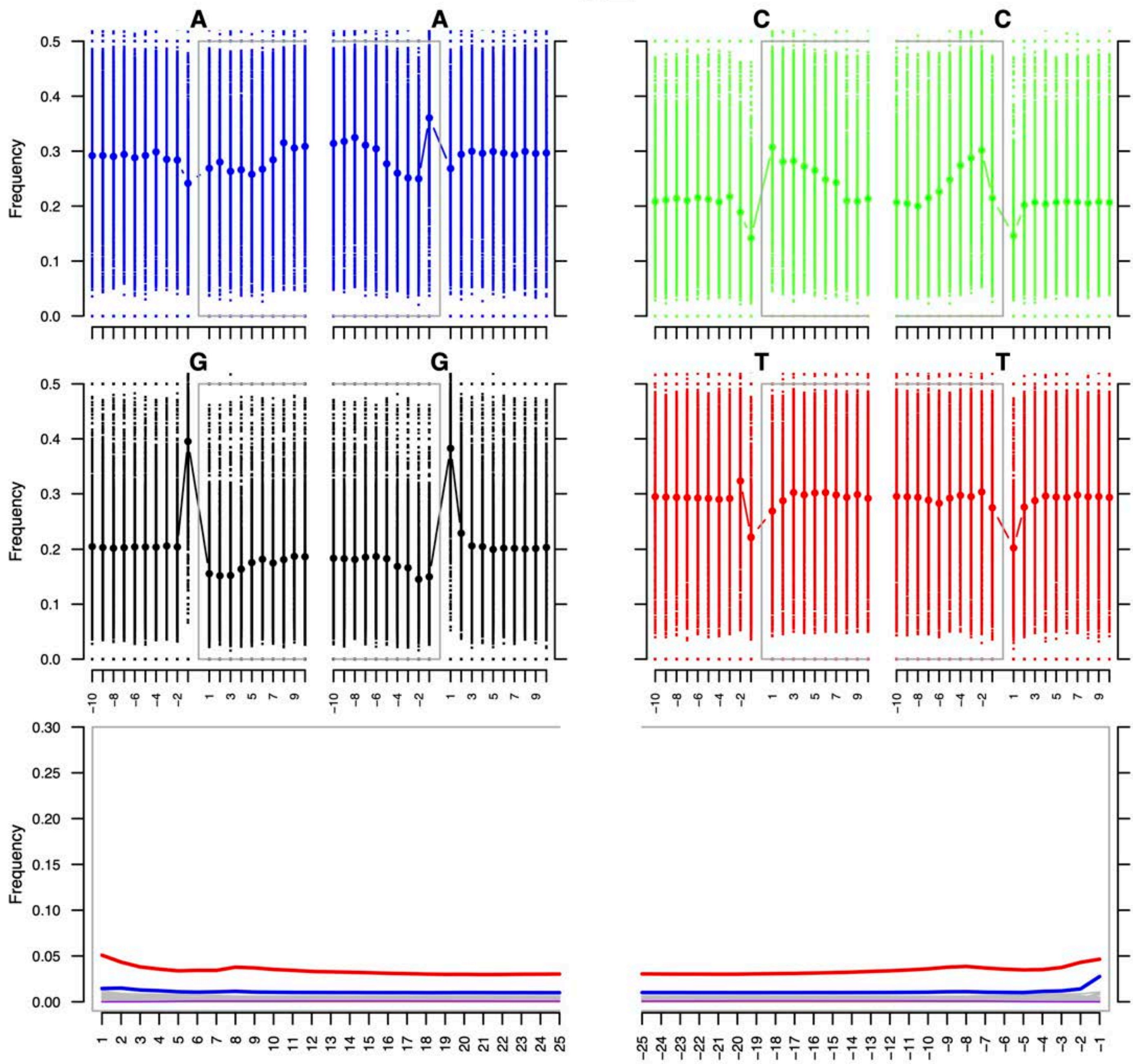

VI4

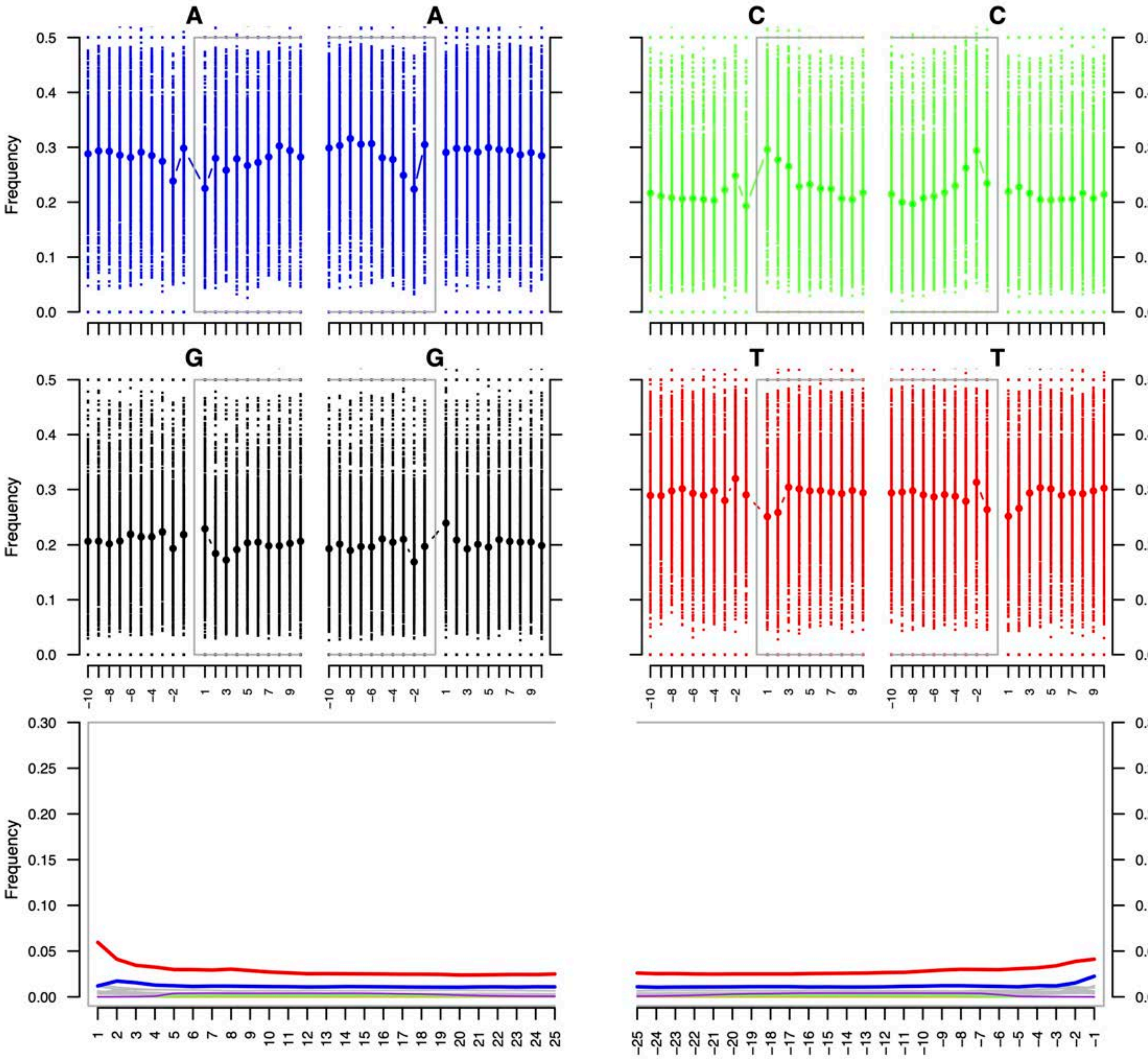

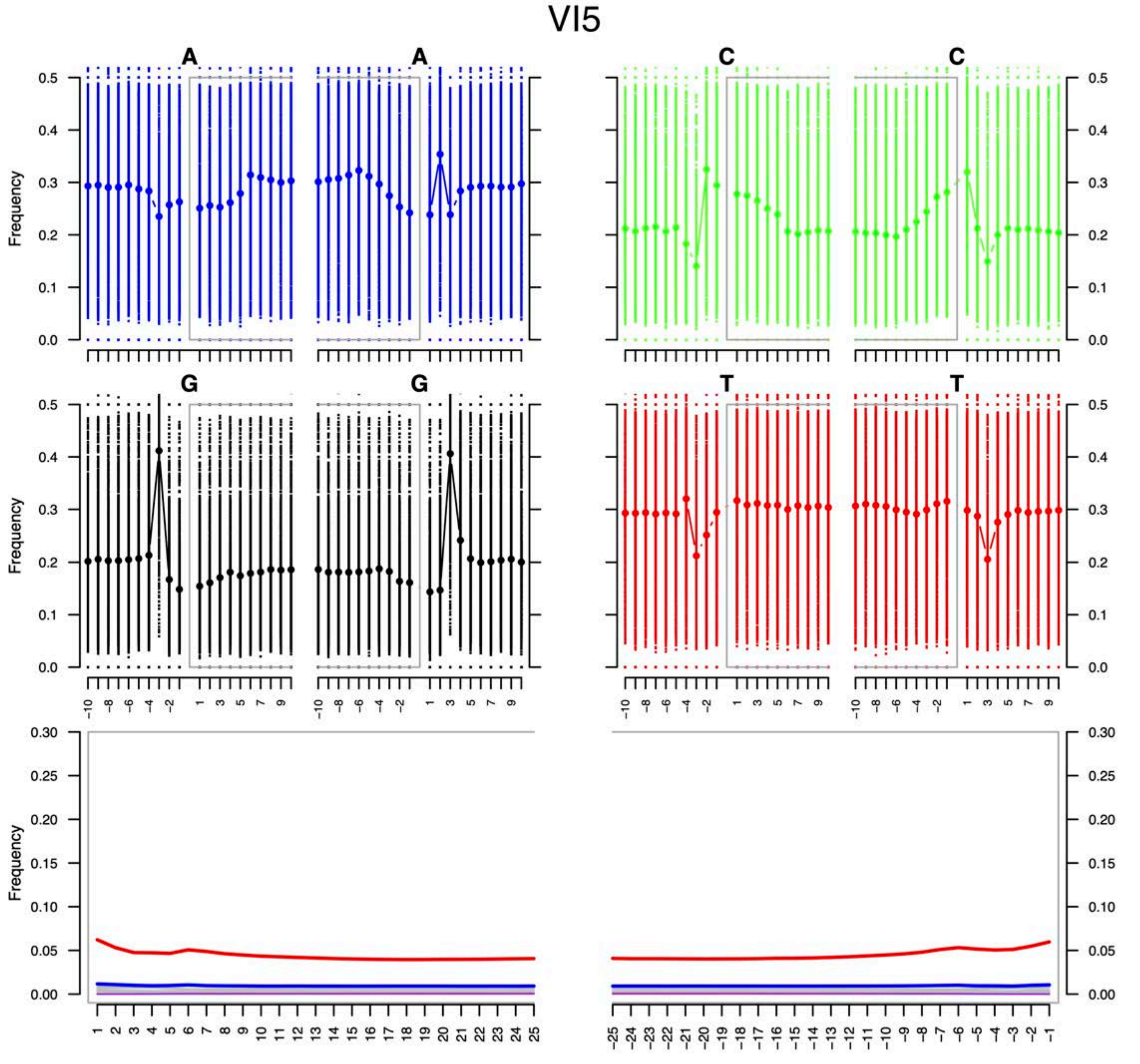

VI5
A
A
C
C
G
G
T
T
Frequency
Frequency
Frequency

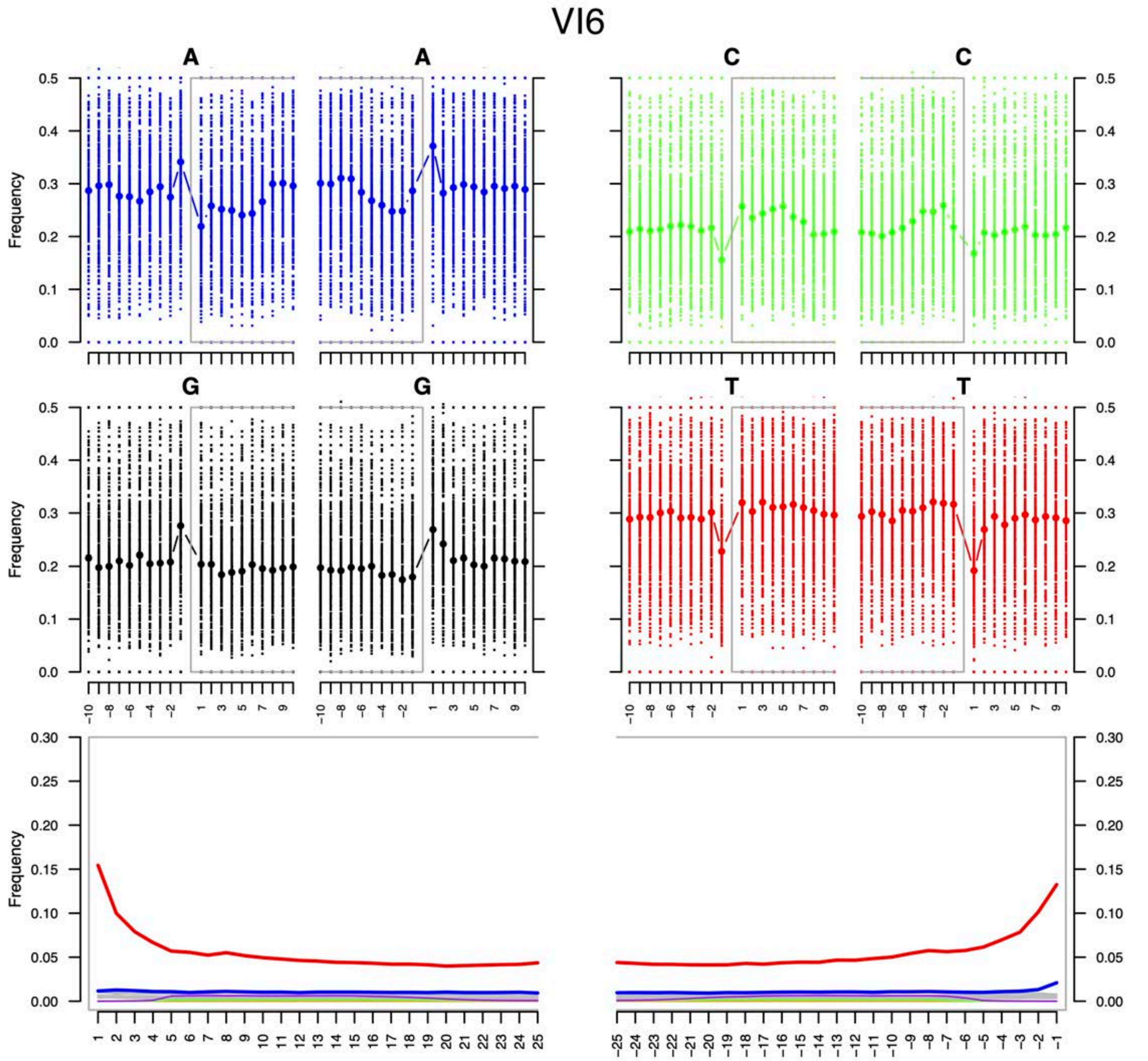

VI6
A
A
C
C
G
G
T
T
Frequency

VI7

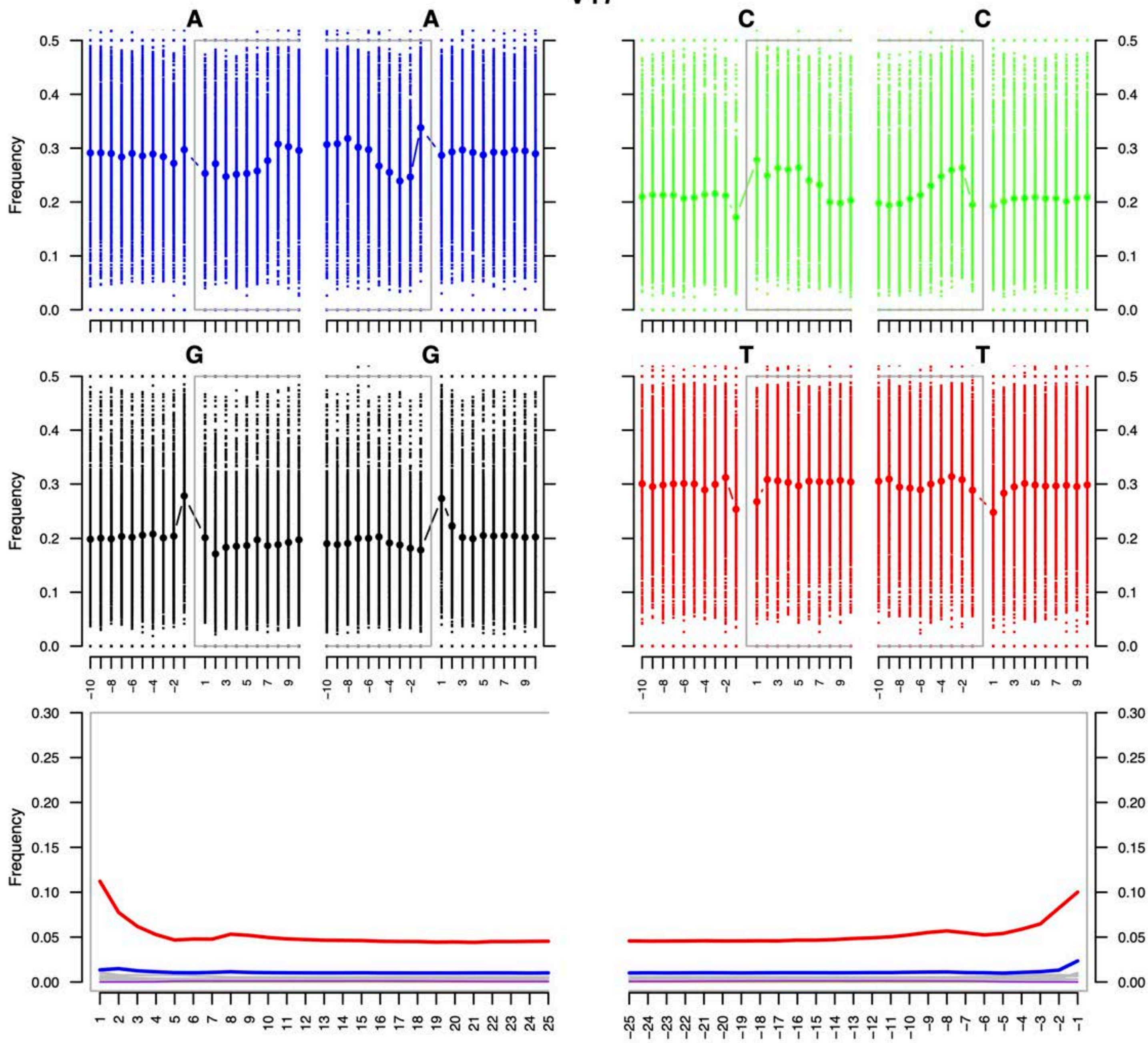

VI8

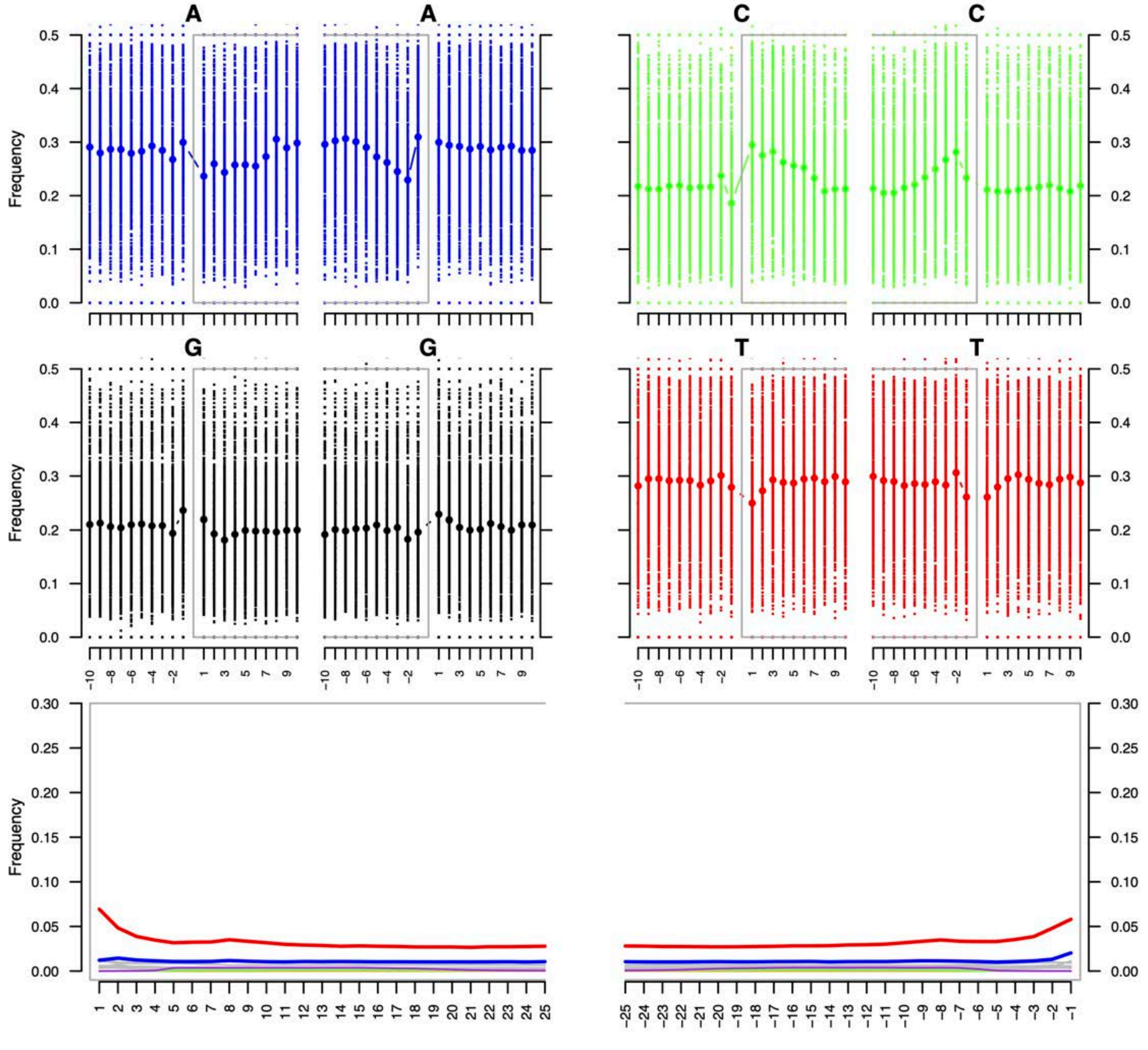

VI9

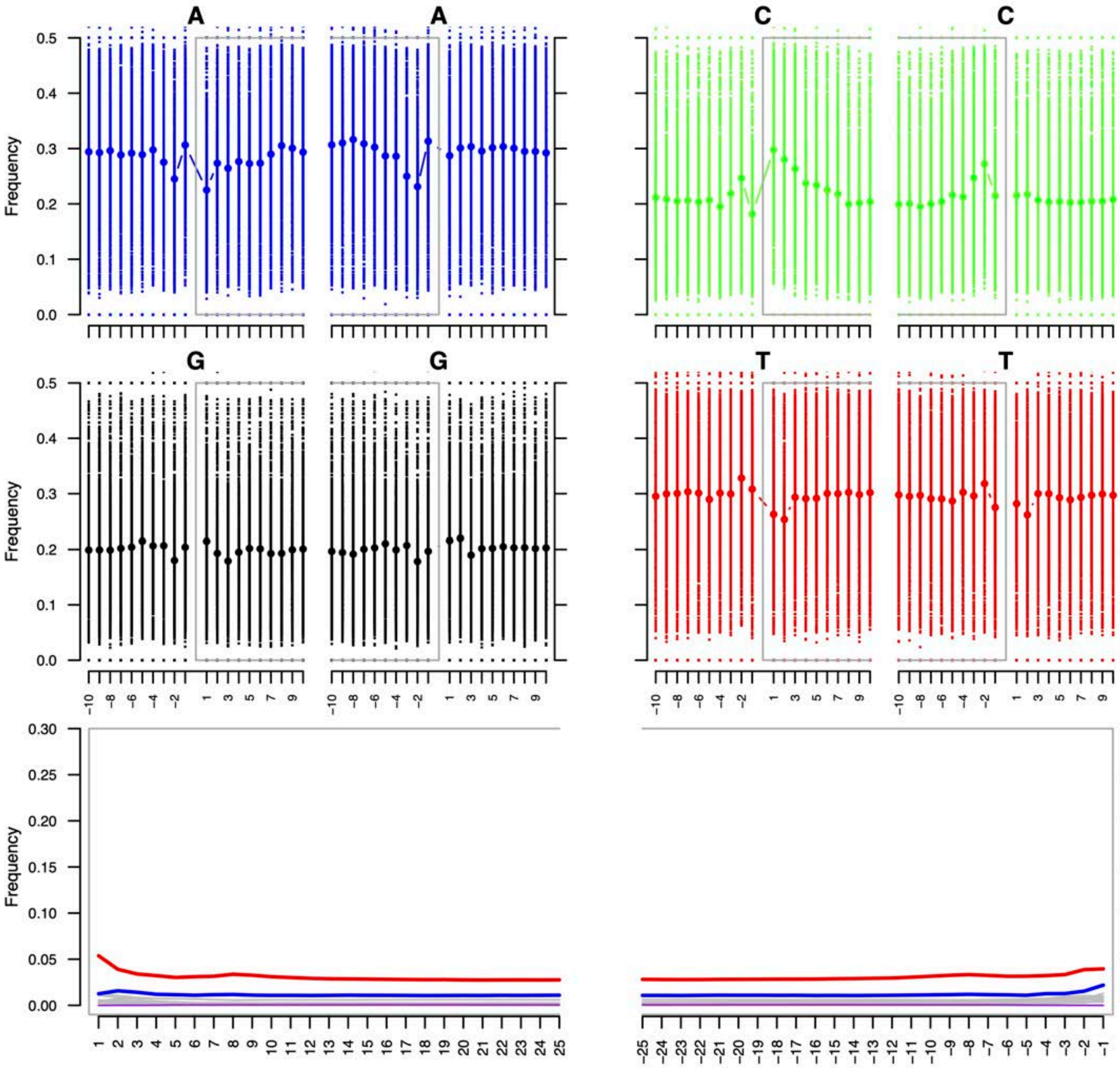

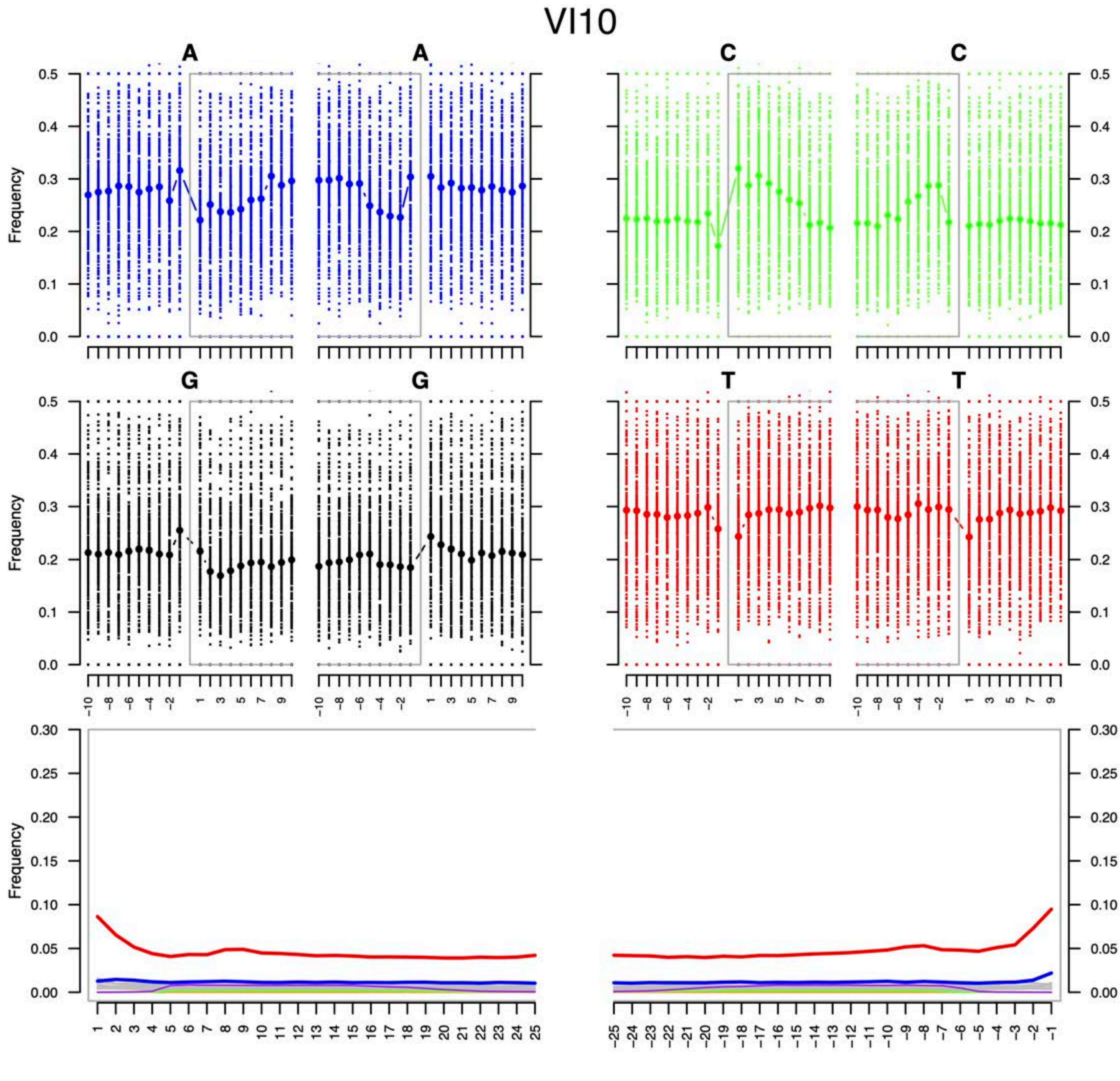
VI10
A
A
C
C
G
G
T
T
Frequency
Frequency
Frequency

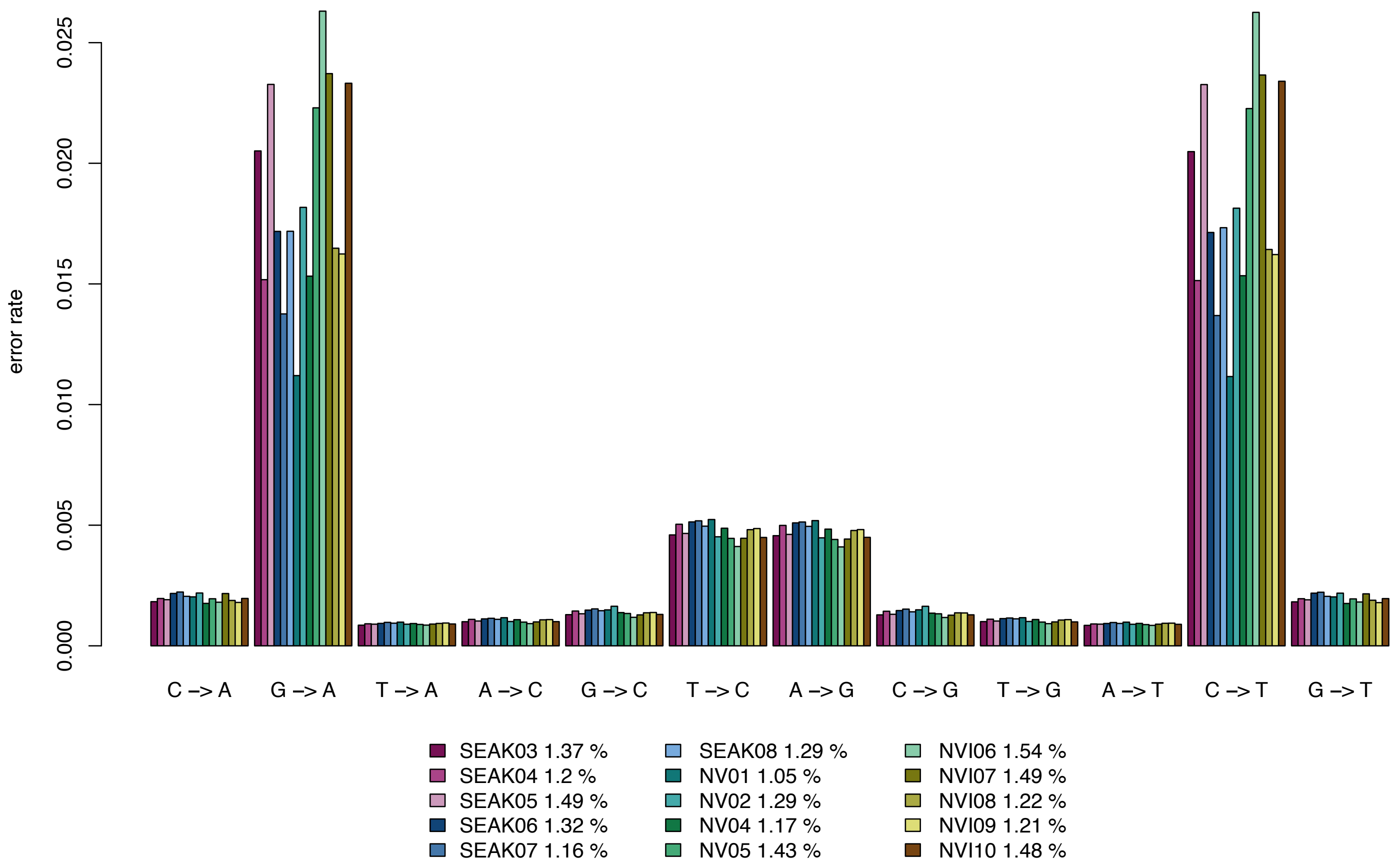
error rate
0.000
0.005
0.010
0.015
0.020
0.025
C –> A
G –> A
T –> A
A –> C
G –> C
T –> C
A –> G
C –> G
T –> G
A –> T
C –> T
G –> T
SEAK03 1.37 %
SEAK04 1.2 %
SEAK05 1.49 %
SEAK06 1.32 %
SEAK07 1.16 %
SEAK08 1.29 %
NV01 1.05 %
NV02 1.29 %
NV04 1.17 %
NV05 1.43 %
NVI06 1.54 %
NVI07 1.49 %
NVI08 1.22 %
NVI09 1.21 %
NVI10 1.48 %

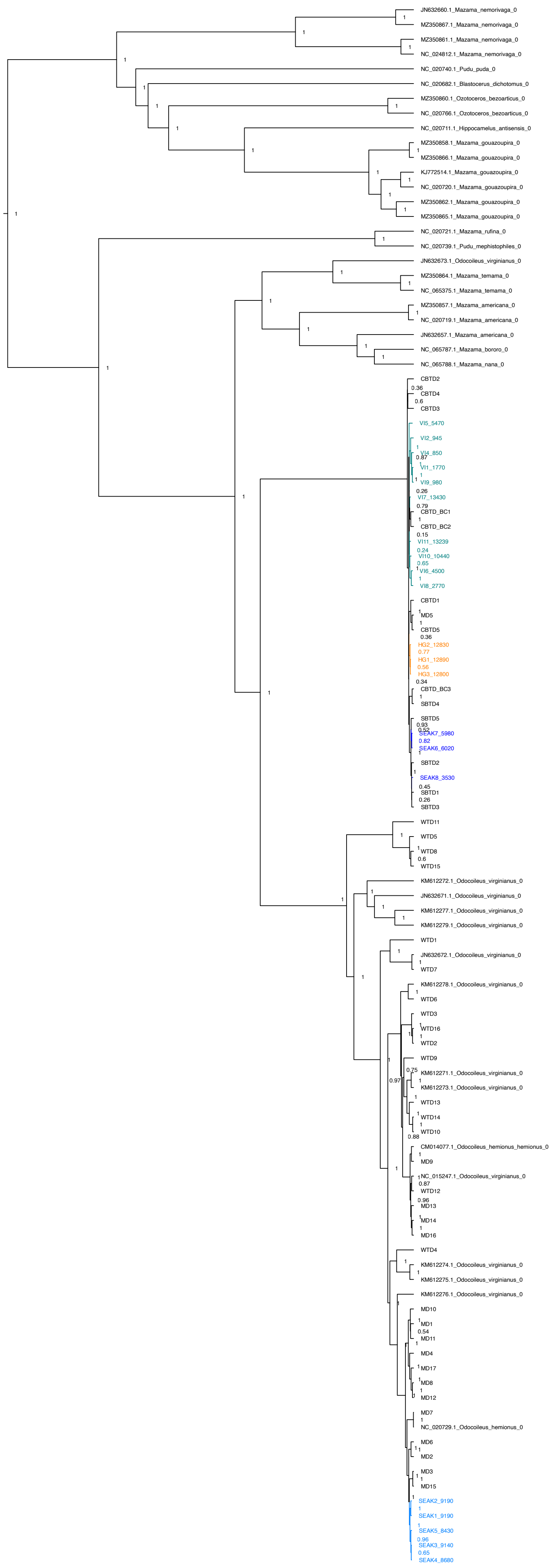

JN632660.1_Mazama_nemorivaga_0
MZ350867.1_Mazama_nemorivaga_0
MZ350861.1_Mazama_nemorivaga_0
NC_024812.1_Mazama_nemorivaga_0
NC_020740.1_Pudu_puda_0
NC_020682.1_Blastocerus_dichotomus_0
MZ350860.1_Ozotoceros_bezoarticus_0
NC_020766.1_Ozotoceros_bezoarticus_0
NC_020711.1_Hippocamelus_antisensis_0
MZ350858.1_Mazama_gouazoupira_0
MZ350866.1_Mazama_gouazoupira_0
KJ772514.1_Mazama_gouazoupira_0
NC_020720.1_Mazama_gouazoupira_0
MZ350862.1_Mazama_gouazoupira_0
MZ350865.1_Mazama_gouazoupira_0
NC_020721.1_Mazama_rufina_0
NC_020739.1_Pudu_mephistophiles_0
JN632673.1_Odocoileus_virginianus_0
MZ350864.1_Mazama_temama_0
NC_065375.1_Mazama_temama_0
MZ350857.1_Mazama_americana_0
NC_020719.1_Mazama_americana_0
JN632657.1_Mazama_americana_0
NC_065787.1_Mazama_bororo_0
NC_065788.1_Mazama_nana_0
CBTD2
CBTD4
CBTD3
VI5_5470
VI2_945
VI4_850
VI1_1770
VI9_980
VI7_13430
CBTD_BC1
CBTD_BC2
VI11_13239
VI10_10440
VI6_4500
VI8_2770
CBTD1
MD5
CBTD5
HG2_12830
HG1_12890
HG3_12800
CBTD_BC3
SBTD4
SBTD5
SEAK7_5980
SEAK6_6020
SBTD2
SEAK8_3530
SBTD1
SBTD3
WTD11
WTD5
WTD8
WTD15
KM612272.1_Odocoileus_virginianus_0
JN632671.1_Odocoileus_virginianus_0
KM612277.1_Odocoileus_virginianus_0
KM612279.1_Odocoileus_virginianus_0
WTD1
JN632672.1_Odocoileus_virginianus_0
WTD7
KM612278.1_Odocoileus_virginianus_0
WTD6
WTD3
WTD16
WTD2
WTD9
KM612271.1_Odocoileus_virginianus_0
KM612273.1_Odocoileus_virginianus_0
WTD13
WTD14
WTD10
CM014077.1_Odocoileus_hemionus_hemionus_0
MD9
NC_015247.1_Odocoileus_virginianus_0
WTD12
MD13
MD14
MD16
WTD4
KM612274.1_Odocoileus_virginianus_0
KM612275.1_Odocoileus_virginianus_0
KM612276.1_Odocoileus_virginianus_0
MD10
MD1
MD11
MD4
MD17
MD8
MD12
MD7
NC_020729.1_Odocoileus_hemionus_0
MD6
MD2
MD3
MD15
SEAK2_9190
SEAK1_9190
SEAK5_8430
SEAK3_9140
SEAK4_8680

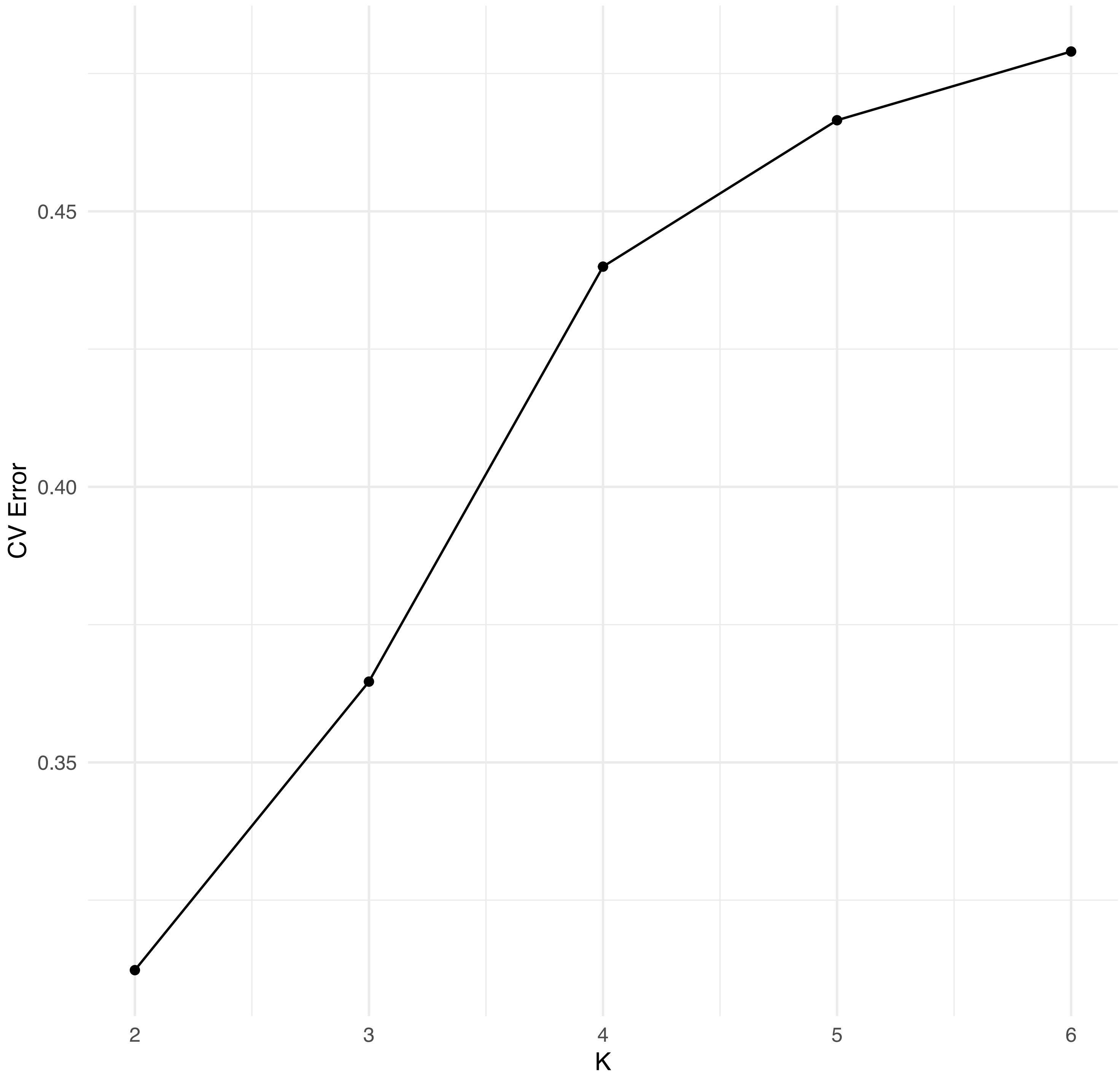
0.45
0.40
0.35
CV Error
2
3
4
5
6
K

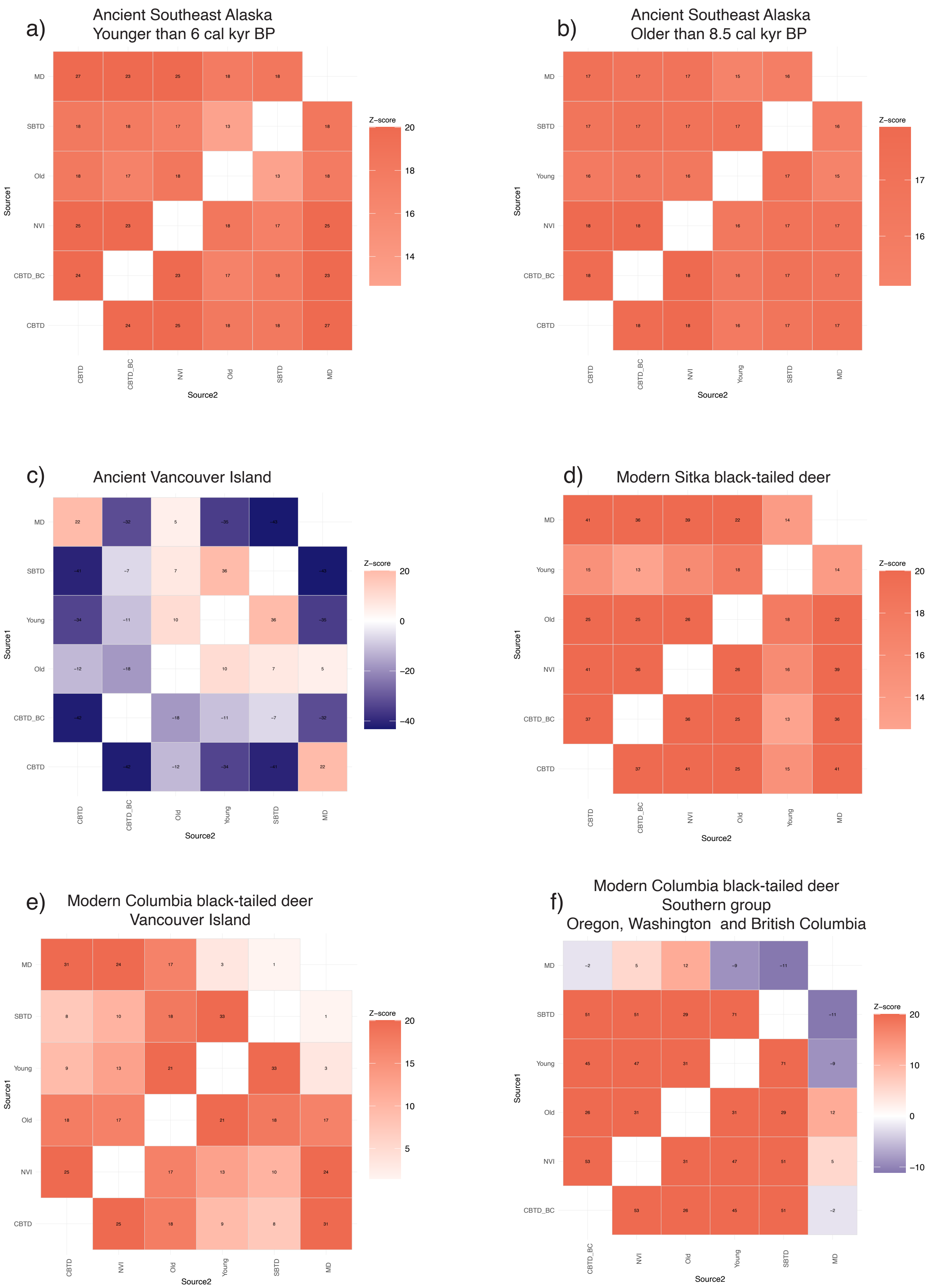

a)
Ancient Southeast Alaska
Younger than 6 cal kyr BP
MD
SBTD
Old
NVI
CBTD_BC
CBTD
Source1
Source2
Z-score
20
18
16
14
b)
Ancient Southeast Alaska
Older than 8.5 cal kyr BP
MD
SBTD
Young
NVI
CBTD_BC
CBTD
Source1
Source2
Z-score
17
16
c)
Ancient Vancouver Island
MD
SBTD
Young
Old
CBTD_BC
CBTD
Source1
Source2
Z-score
20
0
−20
−40
d)
Modern Sitka black-tailed deer
MD
Young
Old
NVI
CBTD_BC
CBTD
Source1
Source2
Z-score
20
18
16
14
e)
Modern Columbia black-tailed deer
Vancouver Island
MD
SBTD
Young
Old
NVI
CBTD
Source1
Source2
Z-score
20
15
10
5
f)
Modern Columbia black-tailed deer
Southern group
Oregon, Washington and British Columbia
MD
SBTD
Young
Old
NVI
CBTD_BC
Source1
Source2
Z-score
20
10
0
−10
